# MicroRNA Expression Is Down-Regulated and Reorganized in Prefrontal Cortex of Depressed Suicide Subjects

**DOI:** 10.1371/journal.pone.0033201

**Published:** 2012-03-09

**Authors:** Neil R. Smalheiser, Giovanni Lugli, Hooriyah S. Rizavi, Vetle I. Torvik, Gustavo Turecki, Yogesh Dwivedi

**Affiliations:** 1 Department of Psychiatry, Psychiatric Institute, University of Illinois at Chicago, Chicago, Illinois, United States of America; 2 Graduate School of Library and Information Science, University of Illinois at Urbana-Champaign, Champaign, Illinois, United States of America; 3 McGill Group for Suicide Studies, McGill University, Montreal, Quebec, Canada; Louisiana State University Health Sciences Center, United States of America

## Abstract

**Background:**

Recent studies suggest that alterations in expression of genes, including those which regulate neural and structural plasticity, may be crucial in the pathogenesis of depression. MicroRNAs (miRNAs) are newly discovered regulators of gene expression that have recently been implicated in a variety of human diseases, including neuropsychiatric diseases.

**Methodology/Principal Findings:**

The present study was undertaken to examine whether the miRNA network is altered in the brain of depressed suicide subjects. Expression of miRNAs was measured in prefrontal cortex (Brodmann Area 9) of antidepressant-free depressed suicide (n = 18) and well-matched non-psychiatric control subjects (n = 17) using multiplex RT-PCR plates. We found that overall miRNA expression was significantly and globally down-regulated in prefrontal cortex of depressed suicide subjects. Using individual tests of statistical significance, 21 miRNAs were significantly decreased at p = 0.05 or better. Many of the down-regulated miRNAs were encoded at nearby chromosomal loci, shared motifs within the 5′-seeds, and shared putative mRNA targets, several of which have been implicated in depression. In addition, a set of 29 miRNAs, whose expression was not pairwise correlated in the normal controls, showed a high degree of co-regulation across individuals in the depressed suicide group.

**Conclusions/Significance:**

The findings show widespread changes in miRNA expression that are likely to participate in pathogenesis of major depression and/or suicide. Further studies are needed to identify whether the miRNA changes lead to altered expression of prefrontal cortex mRNAs, either directly (by acting as miRNA targets) or indirectly (e.g., by affecting transcription factors).

## Introduction

Depression is a major public health concern. Despite many years of research, the molecular and cellular mechanisms associated with depression are not clear. Recent studies suggest that alterations in expression of genes, including those which regulate neural and structural plasticity, may be crucial in the pathogenesis of depression [1], [2]. In the past few years, it has become clear that besides the traditional transcriptional mechanisms (e.g., by transcription factors and alternative splicing), gene expression is also regulated by a variety of noncoding RNA transcripts that generate antisense RNAs, microRNAs (miRNAs), and other small RNAs, which are linked to several post-transcriptional and epigenetic mechanisms. Of these, the best investigated are the microRNAs, which were first identified as regulators of developmental timing and cell fate, and later were found to comprise a population of hundreds of distinct genes that are widely expressed in nearly all multicellular organisms and in many tissues, including adult mammalian brain.

The miRNAs are encoded within primary miRNA gene transcripts (pri-miRs) that may be intergenic (away from known protein-coding genes) or located within introns of protein-coding host genes [3], [4]. Pri-miRs are transcribed by pol II, may be spliced, and may acquire a poly-A+ tail; they are then processed further within the nucleus by Drosha and other co-factors to form one or a series of ∼70–110 nt. small hairpin miRNA precursors, or (pre-miRs) that fold into a stem-loop structure. The pre-miRs are transported out of the nucleus and processed by Dicer to form a double-stranded small RNA about 22 nt. long. Generally, one of these strands is preferentially incorporated into a complex with one or more Argonaute homolog proteins (isoforms of eIF2c). Thus, one has a single-stranded mature miRNA of ∼22 nt. in length, which, together with eIF2c and other associated proteins such as FMRP, comprises the so-called RISC complex. The RISC complex binds to specific “short seed” sequences located predominantly within the 3′-UTR region of mRNAs, which can interfere with translation and/or stability of the mRNA. Besides the direct sequence-specific interaction of RISC with mRNAs, other proteins that bind nearby sites within the 3′-UTR (e.g., FMRP homologues, HuB family members and other ARE binding proteins) may control the magnitude of miRNA effects. Indeed, in certain situations, miRNAs may actually enhance rather than inhibit translation [5]. MiRNAs can also inhibit or stimulate transcription of certain genes [6]. miRNAs tend to act in co-regulated groups, and to affect groups of targets as part of larger gene expression networks that contain feedback and feedforward regulatory loops [7].

There is emerging evidence that miRNAs can contribute to risk of neuropsychiatric disorders, particularly Huntington disease [8], [9], Parkinson disease [10], [11], and Tourette's syndrome [12]. Recently, several studies have reported altered levels of specific miRNAs in postmortem brain of subjects who died with a diagnosis of schizophrenia [13], [14], [15], [16]. These changes did not appear to be secondary to neuroleptic treatment [14]. As well, in a case-control study, single nucleotide polymorphic analysis identified two brain-expressed miRNAs as being linked to schizophrenia [17]. So far, there is no direct study examining the role of brain miRNA in mood disorders; however, it has been shown that stress, glucocorticoids and mood-stabilizers modulate expression of selective miRNAs [18]. In recent studies of mouse olfactory discrimination training [19] and the learned helpless model of behavioral depression [20], we observed that many miRNAs exhibited a wide range of expression values across individuals and were dynamically sensitive to environmental, sensory and contextual cues. The present study comprehensively examines miRNA expression analysis in depressed suicide subjects. The PFC plays a relevant role in mood regulation and has been associated with a number of neurobiological abnormalities implicated in the pathophysiology of depression and suicide [21], [22].

## Materials and Methods

### Tissue Preparation

The study was approved by the institutional review board of the University of Illinois at Chicago. Tissues were obtained from the Quebec Suicide Brain Bank. The activities of the Quebec Suicide Brain Bank are approved by the Douglas Hospital McGill University IRB. Family members/informants signed written informed consents. For identification and dissection of neuroanatomical regions, we relied on experienced histopathologists using reference neuroanatomical maps [23]. Gyri and sulci were used to landmark specific frontal cortical areas. More precisely, the frontal lobes were sliced into 1–1.5 mm thick coronal sections. The dorsomedial PFC (Brodmann's area 9) was taken just dorsal to the frontopolar area including the most polar portion of the superior and partly the middle superior gyrus between the superior and intermediate frontal sulci. In the sections of the dissected cortical area, the gray and white matters were separated and the gray matter was used in this study. All the tissues were screened for evidence of neuropathology by experienced neuropathologists. pH of the brain was measured in cerebellum in all cases as described by Harrison et al. [24].

### Diagnosis

Psychiatric diagnoses of subjects were made by means of the psychological autopsy method. This technique, which has been validated for Axis I and II diagnoses [25], [26] consists in part of selecting a family member who is best acquainted with the deceased to serve as an informant and undergo the interview process. Informants included primarily mother, father, siblings, and significant other friends, or relatives. In our previous studies, the type of informant made no significant difference in rating the specific disorders identified [27]. Families are recruited at the Montreal Morgue and are interviewed, on average, 4 months after the death. The activities of the Quebec Suicide Brain Bank are approved by the Douglas Hospital McGill University IRB. Family members/informants sign written informed consents. Psychiatric diagnoses are obtained using the SCID [28] interview for axis I DSM-IV diagnoses, as well as the SCID II. Family members are requested to give written permission for clinical records to be obtained from prior mental health treatment providers. An attempt is made to collect all available records on each case, and then the appropriate data are extracted from the records and collected. Information collected through SCID I and II interviews and from the Coroner's notes and medical records is used by interviewers to write a case history for each subject. These case histories are then reviewed by a clinical panel, in order to reach a consensus DSM-IV diagnosis for each subject. All normal controls are verified as being free of mental illness by the same diagnostic process. Inter-rater reliability is routinely checked and periodic interviewer retraining is performed to avoid drifting. Toxicology screening for alcohol, a comprehensive battery for illicit drug use, and screening for antidepressant or psychoactive drugs taken prior to death were performed in blood/urine of each subject. Subjects were negative for all the toxicology screens. Characteristics of normal controls and suicide subjects are provided in **Table 1**. There were no significant differences in age, PMI, or pH of the brain between depressed suicide and normal control subjects.

**Table 1:** Characteristics of subjects in the depressed suicide and normal control groups.

Subject No.	Age (y)	Sex	PMI (h)	Brain pH	RIN	Cause of Death
**Depressed Suicide Group**						
1	40	Male	24	6.2	9.1	Hanging
2	19	Male	29	6.3	9.2	Hanging
3	53	Male	29	6.3	9.0	Hanging
4	35	Male	30	6.7	8.9	Hanging
5	39	Male	15	6.6	9.1	Hanging
6	22	Male	11	6.4	9.3	Hanging
7	49	Male	30	6.6	9.1	Hanging
8	26	Male	34	6.7	8.88	Hanging
9	40	Male	22	6.7	9.2	Hanging
10	18	Male	27	6.8	9.2	CO poisoning
11	38	Male	20	6.6	8.7	Hanging
12	48	Male	15	6.6	9.2	Hanging
13	22	Male	24	6.8	8.8	Jumped
14	55	Female	36	6.5	9.3	Hanging
15	38	Male	40	6.4	8.8	Intoxication
16	65	Female	40	6.5	8.7	Jumped
17	51	Male	46	6.4	8.7	Intoxication
18	63	Male	50	6.4	8.5	Hanging
**Mean**	40.0		29	6.5	8.98	
**SD**	14.6		10.7	0.2	0.24	
**Control Group**						
29	31	Male	26	6.0	9.0	Cardiac arrest
30	19	Male	32	6.6	8.7	Car accident
31	47	Male	24	6.5	9.1	Cardiac arrest
32	30	Male	30	6.4	9.3	Cardiac arrest
33	28	Male	27	6.3	9.2	Car accident
34	41	Male	24	6.0	9.0	Cardiac arrest
35	31	Male	28	6.7	9.1	Car accident
36	32	Male	26	6.7	9.3	Drug overdose
37	46	Male	21	6.6	8.7	Cardiac arrest
38	21	Male	46	6.4	9.0	Cardiac arrest
39	27	Male	25	6.4	8.6	Cardiac arrest
40	32	Male	27	6.7	9.2	Cardiac arrest
41	45	Male	27	6.6	9.0	Cardiac arrest
42	15	Male	27	6.6	9.1	Accidental death
43	38	Male	30	6.8	8.6	Natural death
44	57	Male	25	6.7	9.1	Accidental death
45	63	Male	13	6.5	9.1	Accidental death
**Mean**	35.5		26.9	6.5	9.01	
**SD**	13.0		6.4	0.2	0.22	

### RNA Isolation

Total RNA was isolated in samples of prefrontal cortex (Brodmann Area 9) using a modified protocol designed to optimize recovery of small RNAs [29]. Total RNA was isolated with Trizol reagent (Invitrogen Life Technologies, Carlsbad, CA, USA) according to manufacturer's directions with a few modifications to maximize yield of small RNAs. Glycoblue 20 µg (Ambion) was added to the RNA precipitation step which was allowed to proceed overnight at −20°C. The RNA pellet was spun down at 20,000 g for 25 min at 4°C; rinsed with 80% ethanol in DEPC-treated water (Invitrogen Life Technologies, Carlsbad, CA, USA); resuspended and treated with RNAsecure (Ambion); and treated with DNase I using DNA-free (Ambion). RNA was treated with DNAse I and checked for purity by OD 260/280 ratio (NanoDrop 1000 spectrophotometer, Thermoscientific, Wilmington, DE, USA). The quality of total RNA was verified for all subjects by running on agarose gels. Quality of total RNA was also checked by determining RNA integrity number (RIN) and only samples showing RIN>8.5 were included.

### miRNA Analysis

Reverse transcription was performed with the TaqMan® miRNA Reverse Transcription kit (ABI: Applied Biosystems) and the multiplex RT for TaqMan® miRNA Assays (ABI) that consists of eight predefined reverse transcription primer pools (ABI) following the manufacturer's protocol. For each RT pool, 100 ng of total RNA was used and the product was diluted 1∶62.5 and 55 µl mixed with 55 µl of TaqMan® Universal PCR Master Mix, No AmpErase® UNG. 100 µl of each mix was dispensed in the appropriate well in the TaqMan® Human miRNA Array v1.0 (TLDA, ABI) and run to 50 cycles as per manufacturer's protocol on a ABI 7900HT. An equal number of control and depressed suicide samples were processed and assayed on the same day, by an individual who was unaware of group identity. A sample processed without reverse transcription showed no detectable miRNA values.

Using 4 samples run on duplicate plates to monitor inter-plate reliability, we observed that Ct values above 35 were markedly less reliable, and so Ct = 35 was set as the threshold of detectability. Four data points were removed as outliers (defined as measurements that were at least 3 SD different than all other measurements of the same miRNA; in all cases, there was no more than one outlier observed for a single miRNA); however, removal of outliers did not affect the results reported here. Ct values were normalized using U6 RNA, whose mean value did not vary across groups and showed low variability. In the first 15 samples in each group, exogenous synthetic mir-122a was spiked into samples at the time of RNA extraction as a further control to check for possible differences in sample yield, integrity or loading; results were similar whether normalizing to the endogenous (U6) or exogenous (mir-122a) RNA. Note that Ct values cannot be compared directly across different miRNAs, but when comparing the same miRNA in different samples, a higher Ct value indicates lower abundance, and a difference in Ct values of 1 indicates a two-fold difference in abundance.

### Western Blot Analysis of Target Proteins DNMT3b, BCL2, and VEGFA

Tissues were homogenized on ice with a glass-Teflon homogenizer in lysis buffer (50 mM Tris–hydrogen chloride, pH 7.5, 100 mM sodium chloride, 1% Triton X-100, 50 mM sodium fluoride, 10 mM sodium phosphate, 5 mM EDTA, 1 µg/mL aprotinin, 1 µg/mL leupeptin, 1 mM sodium orthovanadate, and 1 mM 4-[2-aminoethyl] benzenesulfonyl fluoride hydrochloride). The homogenates were centrifuged at 14,000 *g* for 10 minutes. The protein content in the supernatant was determined by the Bradford method (Bio-Rad, Hercules, CA).

Samples containing equal amount of protein (30 µg) were resolved onto 10% (wt/v) sodium dodecyl sulfate (SDS)–polyacrylamide gel and blotted on an enhanced chemiluminescence (ECL) membrane (Amersham) as described earlier (30). Membranes were incubated with human-specific DNMT3b (LifeSpan Bio, Seattle, WA), BCL2 (Abcam, San Francisco, CA), or VEGFA (LifeSpan Bio, Seattle, WA) antibody overnight at 4°C. The dilution for each antibody was as follows: DNMT3b, 1∶500; BCL2, 1∶1000; VEGFA 1∶1000. The ECL membranes were then incubated with horseradish peroxidase–linked secondary antibody (anti-mouse or anti-rabbit IgG, 1∶1000) for 5 hours at room temperature and exposed to ECL autoradiography film. Membranes were stripped and reprobed with endogenous protein β-actin antibody (Sigma Chemical Co, St. Louis, MO) as described earlier [30]. The optical densities of the bands were quantified using the Loats Image Analysis System (Loats Associates, Inc, Westminster, MD). Ratios of optical densities of DNMT3b, BCL2, or VEGFA to β-actin were calculated. DNMT3b, BCL2, and VEGFA migrated to 96, 26, and 22 kDa, respectively. Immunolabeling of β-actin was not significantly different in depressed subjects (1.1±0.2 AU) compared with normal controls (1.2±0.2 AU).

The specificity of DNMT3b, BCL2, and VEGFA antisera were checked by using a 100-fold excess of blocking peptide (relative to the molarity of the antisera) corresponding to the epitope used to generate DNMT3b, BCL2, and VEGFA. In addition, to validate our data, we initially determined the immunolabeling of DNMT3b, BCL2, and VEGFA in the PFC of depressed suicide and control subjects using 5 different concentrations of protein (5–50 µg). It was observed that the optical density of the band increased linearly with increased concentration of protein and that the curve shifted toward the right when a decrease in immunolabeling was observed (data not shown).

### Statistical Analysis

The non-parametric Wilcoxon sign-rank test, 2-tailed was utilized when making lists of miRNAs whose mean expression levels differed significantly across treatment groups, since this test is appropriate for all miRNAs whether or not they follow a normal distribution [31]. The Bonferroni correction of statistical significance values was not appropriate in this study because it assumes that the expression of the vast majority of genes is independent of each other. This situation does not apply in the case of miRNAs, which form extensive cross-correlation networks –in some cases due to co-transcription from the same primary gene transcripts [29], [32], and in some cases due to shared factors that co-regulate their expression within a given tissue or even within a disease condition (see Results). Instead, we performed SAM analysis (Significance Analysis of Microarrays software, version 3.08, Stanford University, http://www-stat.stanford.edu/~tibs/SAM/),which estimated statistical significance by subjecting the data to multiple random permutations; parameters were set for two-class unpaired analysis, Wilcoxon statistic and 5000 permutations.

To measure possible correlations between miRNAs and age, postmortem interval and pH, the latter factors were converted to log2 scores to be comparable with Ct values, and all miRNAs were tested for correlations with each factor across all 35 samples. Given the total sample size (n = 35), a correlation of r = 0.333 is significantly different from r = 0 at the p = 0.05 significance level. The protein levels of DNMT3b, BCL2, and VEGFA were compared between depressed suicide and normal controls using independent sample t-test. The correlations between protein levels of DNMT3b, BCL2, and VEGFA and corresponding miRNAs were calculated using Pearson product-moment correlation analysis.

### Target Prediction

Despite active research in the miRNA field, it is currently not possible to predict accurately which individual brain-expressed mRNAs are most likely to be targeted by a given miRNA [33]. However, we attempted to glean further insights by combining evidence from 11 miRNA target prediction servers using the miRecords meta-server [34]. For each down-regulated miRNA, the meta-server was queried to identify targets that are predicted by at least 3 different prediction servers, and the top 30 ranked targets were tabulated (this is a small fraction of the total number of predictions). We then asked whether certain targets of potential interest were common to many different miRNAs in the down-regulated set of 37 miRNAs listed in **Table 2**.

**Table 2:** microRNAs down-regulated in depressed suicide as determined by individual tests of statistical significance.

	% of control	p-value	chromosomal locus	5′ seed (2–8)	Validated targets (miRecords)
hsa-miR-142-5p	0.635	0.0086	17q22	auaaagu	
hsa-miR-137	0.744	0.0113	1p21.3	uauugcu	CDK6, E2F6, NCOA2
hsa-miR-489	0.496	0.0113	7q21.3	ugacauc	
hsa-miR-148b	0.735	0.0113	12q13.13	cagugca	DNMT3B
hsa-miR-101	0.723	0.0129	1p31.3 (101-1), 9p24.1 (101-2)	acaguac	EZH2, MYCN, ICOS
hsa-miR-324-5p	0.667	0.0129	17p13.1	gcauccc	
hsa-miR-301a	0.721	0.0129	17q22	agugcaa	
hsa-miR-146a	0.772	0.0129	5q33.3	gagaacu	
hsa-miR-335	0.764	0.0148	7q32.2	caagagc	SOX4, PTPRN2, MERTK
hsa-miR-494	0.535	0.0174	14q32.31	gaaacau	
hsa-miR-20b	0.704	0.0245	Xq26.2	aaagugc	VEGFA
hsa-miR-376a*	0.768	0.0277	14q32.31	uagauuc	SLC16A1, SFRS11, TTK
hsa-miR-190	0.715	0.0277	15q22.2	gauaugu	
hsa-miR-155	0.526	0.0277	21q21.3	uaaugcu	AGTR1, BACH1, LDOC1, MATR3, TM6SF1
hsa-miR-660	0.811	0.0277	Xp11.23	acccauu	
hsa-miR-130a	0.764	0.0352	11q12.1	agugcaa	TAC1, CSF1, MAFB, MEOX2, HOXA5
hsa-miR-27a	0.73	0.0352	19p13.12	ucacagu	SP1, SP3, SP4
hsa-miR-497	0.724	0.0395	17p13.1	agcagca	
hsa-miR-10a	0.512	0.0395	17q21.32	acccugu	HOXA1
hsa-miR-20a	0.768	0.0442	13q31.3	aaagugc	VEGFA, E2F1, RUNX1
hsa-miR-142-3p	0.642	0.0495	17q22	guagugu	
**microRNAs decreased by 30% or more (but p>0.05)**					
hsa-miR-211	0.595	0.2274	15q13.3	ucccuuu	
hsa-miR-511	0.598	0.3318	10p12.33	ugucuuu	
hsa-miR-424	0.604	0.1626	Xq26.3	agcagca	NFIA
hsa-miR-369-3p	0.619	0.0759	14q32.31	auaauac	
hsa-miR-597	0.628	0.0615	8p23.1	gugucac	
hsa-miR-496	0.649	0.2274	14q32.31	gaguauu	
hsa-miR-517c	0.658	0.1773	19q13.41	ucgugca	
hsa-miR-184	0.665	0.1626	15q25.1	ggacgga	
hsa-miR-34a	0.668	0.1359	1p36.23	ggcagug	NOTCH1, DLL1, BCL2, E2F3, VEGFA, MYCN
hsa-miR-34b-5p	0.672	0.0557	11q23.1	aggcagu	VEGFA
hsa-miR-24-1*	0.675	0.1089	9q22.32	gccuacu	
hsa-miR-594	0.681	0.554		derived from tRNA	
hsa-miR-34c-5p	0.682	0.2553	11q23.1	ggcagug	
hsa-miR-17*	0.686	0.2659	13q31.3	cugcagu	
hsa-miR-545	0.7	0.1337	Xq13.2	cagcaaa	
hsa-miR-565	0.7	0.1024		derived from tRNA	

### mRNA levels of Dicer, Drosha, DGCR8, and pri-miRs 155 and 494

Total RNA (1 µg) was reverse transcribed using 1.5 µl of a 10 µM mix solution of random hexanucleotides (experiment 1) or short specific RT primers (experiment 2), 10 mM dNTP mix, 0.1 M DTT, 40 units RNaseOUT and 200 units SuperScript™ III. Denaturation was performed at 90°C for 5 min. RT reaction was carried out by 5 min at 38°C, 30 min at 42°C, 30 min at 55°C, in a final reaction volume of 20 µl. Realtime PCR reaction was carried out in a final volume of 20 µl, containing 5 µl of using cDNA (diluted 1∶10), 20 µM each of specific primers, and 1× SYBR green PCR Master Mix (Applied Biosystems). To determine the linear range and sensitivity of each assay, a standard curve was generated using a serial 10-fold dilution of pooled cDNA derived from all subjects. All experiments are performed in duplicate and include a non-RT and no template control. Reactions were run on Mx3005p (Strategene, La Jolla, CA, USA) with cycling conditions: 95°C for 10 min, 40 cycles 95°C for 15 sec and 60°C for 1 min and followed by a melting curve cycle; 95°C for 1 min, 55°C for 30 sec, 95°C for 30 sec. Primer specificity was verified by a single peak in the melting curve analysis and a single band of the right size on gel electrophoresis for the all genes analyzed. **Short RT primers:** Dicer (TM 40.2 C) TTGGTGGACCAA; Drosha (TM 39 C) CGTCCAAATAACTG; DGCR8 (TM 43.6 C) ACGTCCACGGT; U6 (TM 36.2 C) TATGGAACGCTT; U44 (TM 37.2 C) CAGTTAGAGC TAATT; PRI-155 (TM 41.9 C) ACAGCCTACAGC; PRI-494 (TM 37.7 C) TCAAAACAGA ACTG. **qPCR Primers:** DICER (154 bp) Forward: CACATCAATAGATACTGTGCT, Reverse: TTGGTGGACCAACAATGGAGG. DROSHA (64 bp) Forward: AAGCGTTAATA GGAGCTGTTTACT, Reverse: CGTCCAAATAACTGCTTGGCT. DGCR8 (92 bp) Forward: GCTGAGGA AAGGGAGGAG, Reverse: ACGTCCACGGTGCACAG. U6 (95 bp) Forward: CTCGCTTCGGCAGCACA, Reverse: AACGCTTCACGAATTTGCGT. U44 (48 bp) Forward: TGATAGCAAATGCTGACTGA, Reverse: CAGTTAGAGCTA ATTAAGACC. Pri-155 (135 bp) Forward: CCAGCTTTATAACCGCATGT, Reverse: ACAGCCTACAGCAAGC CTTC. Pri-494 (134 bp) Forward: TGACTTCCCAA AAGCAACCT, Reverse: TCAAAACA GAACTGCCGAATC.

## Results

### Global analysis of miRNA expression

The multiplex RT-PCR plate assayed 367 miRNAs in parallel, of which 196 miRNAs exhibited mean expression values at least 2-fold greater than the threshold for detectability (i.e., mean Ct value ≤34 before normalization) in the control group. This set of 196 miRNAs was analyzed further. The complete set of normalized Ct values is included as Supporting Information (**File S1**).

First, miRNA expression values were normalized relative to the small RNA U6; this appears to be a suitable normalizer since U6 showed no significant mean difference across groups and exhibited low, equal variance in both groups. Twenty-one miRNAs were significantly down-regulated (none up-regulated) at p = 0.05. This is a large and consistent response; note that one would expect to observe only 5 miRNAs down-regulated and 5 miRNAs up-regulated by chance alone, at p = 0.05. Similarly, 24 miRNAs were down-regulated but none up-regulated by 30% or more (i.e., delta Ct>0.5). The results were even more striking when SAM analysis (Significance Analysis of Microarrays, Stanford University) was carried out: 100 miRNAs were identified as significantly decreased at p = 0.05 or better, with a false-discovery rate of 2.45% (i.e., fewer than 3 miRNAs would be expected to be significant by chance alone).

In large part, this down-regulation is due to a general, global down-regulation of miRNAs levels in the depressed suicide group. In almost every case, the mean abundance of a given miRNA in the depressed group was lower than its mean abundance in controls (17% decrease on average). This finding was highly significant (p = 4.5×10^−43^). Furthermore, this global down-regulation was also apparent when the data were normalized instead to the geometric mean of U6, U44 and U48 (p = 8.3×10^−21^). Finally, to make sure that the apparent finding was not due to an up-regulation of small RNAs in the depressed group, we examined the 15 samples in each group that had mir-122a spiked-into samples as an exogenous control (mir-122a levels showed no difference across groups). We still observed a highly significant global downregulation of miRNA levels in the depressed group (p = 1×10^−6^).

**Figure 1:**
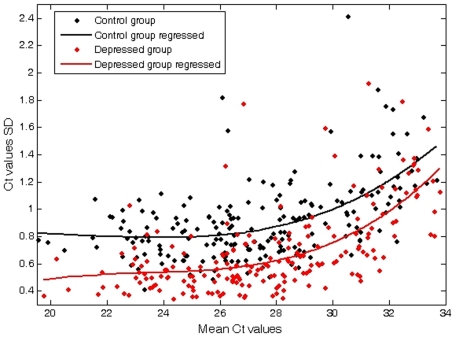
Plot of Mean vs standard deviation (SD) for normal control and depressed suicide groups. miRNA expression in the depressed suicide group is significantly *less* variable than in the control group.

Because the depressed group might be expected to be heterogeneous in terms of underlying etiologies or other variables, we hypothesized that the measured miRNA values in the depressed group might an overall increased extent of heterogeneity relative to the control group. However, by plotting the relation between the standard deviation and the mean expression value across all expressed miRNAs, we found that the miRNA expression in the depressed suicide group was significantly less variable than in the control group (**Fig. 1**).

### Analysis of individual down-regulated miRNAs

Using data normalized to U6, twenty-one miRNAs were significantly decreased (none were increased) when tested individually at p = 0.05 or better (**Table 2**). The majority of these were also significant when the data were normalized instead to the geometric mean of U6, U44 and U48 (data not shown). To examine whether confounding variables had any effect on the expression of individual miRNA, we performed correlational analysis using Pearson product-moment analysis. We did not find any effect of age, PMI, gender or pH of the brain on the expression of miRNAs (data not shown).

The 21 down-regulated miRNAs include many miRNAs that have been implicated in cellular growth and differentiation. The set comprises both very abundant and low-abundance miRNAs, and those with high and low synaptic enrichment as studied in mouse homologues [29]; the miRNAs arise from both intronic and intergenic loci, and are located on numerous chromosomes. An additional 16 miRNAs were decreased by 30% or more that failed to meet the criterion for significance (**Table 2**). This may be due, at least in part, to their much lower expression – only 3 of 16 miRNAs in the non-significant set were abundantly expressed (i.e., mean Ct value<30), vs. 18 of 21 miRNAs in the significant set. Nevertheless, because many of the non-significant miRNAs shared chromosomal loci, 5′-seeds, and predicted targets with the significant miRNAs, they were also included in the analysis.

Almost half of the combined down-regulated miRNAs are encoded at chromosomal loci near another miRNA on the list (**Table 2**) and are possibly transcribed by the same primary miRNA gene transcripts [32], [35]: a) mir-142-5p and 142-3p; b) mir-494, 376a*, 496, and 369-3p; c) mir-23b, 27b and 24-1*; d) mir-34b* and 34c; and e) mir-17* and 20a. In addition, three pairs of miRNAs are encoded at distances greater than 100 kb but still lie within the same chromosomal region: a) mir-424 and 20b at Xq26.2-3, 377 kb apart; b) mir-142 and 301a at 17q22, 820 kb apart; and c) mir-324-5p and 497 at 17p13.1, 205 kb apart. This suggests that at least some of the down-regulated miRNA expression is due to decreased transcription.

Many of the down-regulated miRNAs also shared 5′-seed sequences (particularly bases 2–8, **Table 2**) that are involved in target recognition [36]. For example, identical seed sequences are shared by a) mir-20a and 20b; b) mir-301a and 130a; and c) mir-424 and 497. As well, a 6-mer nucleotide motif is shared by mir-34a, 34b* and 34c, and strikingly, a 5-mer motif (AGUGC) within the 5′-seed is shared by 5 of the affected miRNAs (mir-148b, 301a, 130a, 20a, 20b) that is predicted to bind Alu sequences within the 3′-UTR region of target mRNAs [37]. This suggests that the down-regulated miRNAs should exhibit extensive overlap among their mRNA targets.

### Target Analysis

At the time that the analysis was carried out, 42 experimentally validated targets were currently known for miRNAs in the down-regulated set, according to curated data compiled at the miRecords server [38], http://mirecords.umn.edu/miRecords/, though it should be noted that validation experiments were generally conducted in non-neural systems. Many of these are transcription factors (e.g., E2F1, E2F6, BACH1, SP1, HOXA5, RUNX1) and other nuclear proteins, but include transmembrane and signaling proteins as well. Intriguingly, 4 different down-regulated miRNAs all target the same validated growth factor, VEGFA (mir-20b, 20a, 34a, 34b*), a molecule implicated in depression in both humans and animal models (see Discussion). Other validated targets include BCL2 (mir-34a), DNMT3B (mir-148b), and MYCN (mir-101, 34a).

Among predicted targets, estrogen receptor alpha, ESR1, was predicted to be targeted by 3 different down-regulated miRNAs (mir-148b, 301a, 496). Others targeted by 3 or more affected miRNAs include ubiquitin ligases (UBE2D1 and UBE2W), signal transduction mediators (CAMK2G, AKAP1), the splicing factor NOVA1 that regulates brain-specific alternative splicing; the GABA-A receptor sub-unit GABRA4; calcium channel CACNA1C; and brain-active transcription factors including SMAD5, MITF, BACH2, MYCN, and ARID4A. Several of these predicted targets interact with validated targets; for example, ARIA4A binds E2F1; SMAD5 binds RUNX1; and estradiol treatment decreases E2F1 levels in prefrontal cortex [39]. BACH2 transcription factor binding sites have been identified upstream of many brain-expressed miRNAs [40]. Retinoblastoma binding protein 1 (ARIA4A) is of interest because it recruits histone deacetylases and regulates gene expression via chromatin-based silencing.

**Figure 2:**
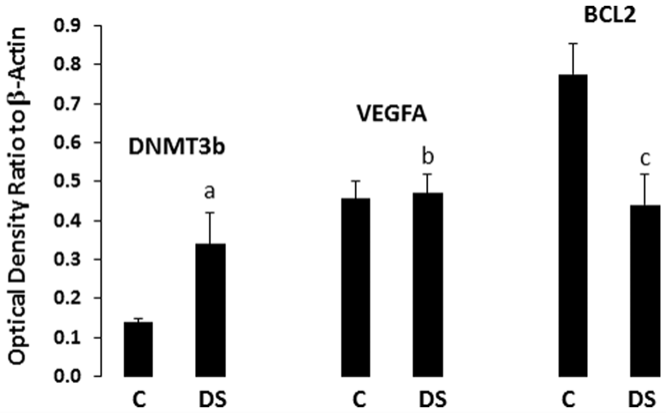
Protein levels of DNMT3b, BCL2, and VEGFA in PFC of depressed suicide (n = 18) and normal control (n = 17) subjects. Protein samples from tissue lysates were subjected to 10% polyacrylamide gel electrophoresis and transferred to enhanced chemiluminescence–nitrocellulose membranes, which were then incubated with primary antibody specific for DNMT3b, BCL2, VEGFA or β-actin and corresponding secondary antibody. The suicide group was compared with the control group. DMNT3b was strongly up-regulated (2.4-fold) in the depressed suicide group (^a^p = 1×10^−11^), VEGFA showed no significant change (1.03-fold, ^b^p = 0.4) and BCL2 was strongly down-regulated (0.57 of control, ^c^p = 2.4×10^−14^). C = Controls; DS = Depressed suicide.

We measured protein levels of several selected target proteins implicated in depression (DMNT3b, VEGFA, and BCL2) in all individuals by Western blotting (normalizing values using beta-acting immunoreactivity). Whereas mean levels of DMNT3b was strongly up-regulated (2.4-fold) in the depressed suicide group (p = 1×10^−11^), VEGFA showed no significant change (1.03-fold, p = 0.4) and BCL2 was strongly down-regulated (0.57 of control, p = 2.4×10^−14^) (**Fig. 2**). Changes in mean expression levels may reflect a variety of influences (e.g. transcription factor activity and differential turnover), as well as possible exerting regulatory effects by miRNAs listed in **Table 2** and others. When we calculated the correlations between miRNA abundance and protein abundance across individual samples within the control group and (separately) within the depressed suicide group, surprisingly, DNMT3b levels showed an extremely strong positive correlation with mir-148b across individuals (r = 0.91 in controls, r = 0.94 in the depressed suicide group). Similarly, BCL2 was strongly and positively correlated with mir-34a (r = 0.92 in controls, r = 0.82 in the depressed suicide group). These findings strongly suggest that these miRNAs are likely to be actively co-regulated with their targets, as often observed in brain [7]. In contrast, VEGFA showed a mix of smaller positive and inverse correlations: (mir-20a: r = 0.08 in controls and r = 0.26 in depressed; mir-20b: r = 0.34 in controls and r = 0.24 in depressed; mir-34a: r = 0.34 in controls and r = −0.39 in depressed; mir-34b-5p: r = −0.363 in controls and r = 0.08 in depressed). Of these, mir-34a is probably the most interesting finding, since the correlation was positive in controls but inverse in the depressed suicide group. This may reflect a reorganization of miRNA-target networks (see below).

### Quantile rank analysis of miRNA expression

Quantile rank analysis is a non-parametric technique that identifies miRNAs which change their expression significantly relative to the other miRNAs in the population. This is an important parameter since different miRNAs often compete for binding to RISC complexes, and therefore changes in quantile rank may be as (or more) relevant for miRNA functional effects as absolute changes in miRNA abundance across groups. The miRNAs in each group were rank-ordered according to their mean expression levels (i.e., were assigned quantile ranks), and we then looked for miRNAs that show significant changes in quantile rank across groups. Overall, changes in quantile rank were relatively small and normally distributed (mean change = 0, SD = 3.6). Although six miRNAs showed a relative increase in quantile ranks relative to other miRNAs in the population (**Table 3**), this finding is not different from what would be expected by chance alone (expected number at p<0.05 or better = 5.3, observed = 6). In contrast, ten miRNAs showed a relative decrease in quantile ranks in excess of what is expected by chance (expected number at p<0.05 or better = 5.3, observed = 10) (**Table 3**). Most of these miRNAs were also listed in **Table 2**, and may be regarded as especially likely to exert functional effects in the depressed suicide group.

**Table 3:** Quantile rank analysis of miRNA expression data.

P	Observed up	Observed down
<0.05	mir-99b, 375, 462, 639	mir-142-5p, 155, 211, 489, 494, 594, 597
<0.02	mir-429, 642	mir-142-3p, 324-5p, 515-3p

### miRNA pairwise correlation analysis

Another complementary method of analyzing the miRNA expression data is to identify pairs of miRNAs that are co-regulated in their expression, up or down, across individuals within a single group. The pairwise correlations for all miRNAs were computed separately within the control group and within the depression group. We were particularly interested in identifying miRNA pairs that showed no significant correlation in the control group, yet *did* exhibit a significant positive correlation in the depressed group. This phenomenon allows one to detect coordinated changes in the underlying driving forces for expression (e.g., due to changes in transcription factors or miRNA processing) in the depressed suicide group.

In this analysis, the list of miRNAs was first filtered to include only those miRNAs that had robust expression (i.e., whose Ct value <35 in all individuals in both control and depressed groups). The Ct values for each miRNA were normalized to the global mean Ct value of the same individual (averaged over all miRNAs), in order to remove correlations due to inter-individual differences in overall miRNA content. Next, all miRNAs were examined pair-wise and the Pearson correlation coefficient was computed for each group separately. (Note that for n = 17 subjects per group, only correlations of r = 0.5 or greater are significantly different from 0 at p = 0.05.) Finally, we identified pairs of miRNAs that satisfied the following criteria: 1. They exhibited a significant positive correlation in the depressed group (r-dep>0.5), but no significant correlation (positive or negative) in the control group (−0.5 <r-control≤0.2). 2. The correlation coefficients in the control group were much lower than in the depressed suicide group (r-dep minus r-control >0.75).

**Figure 3:**
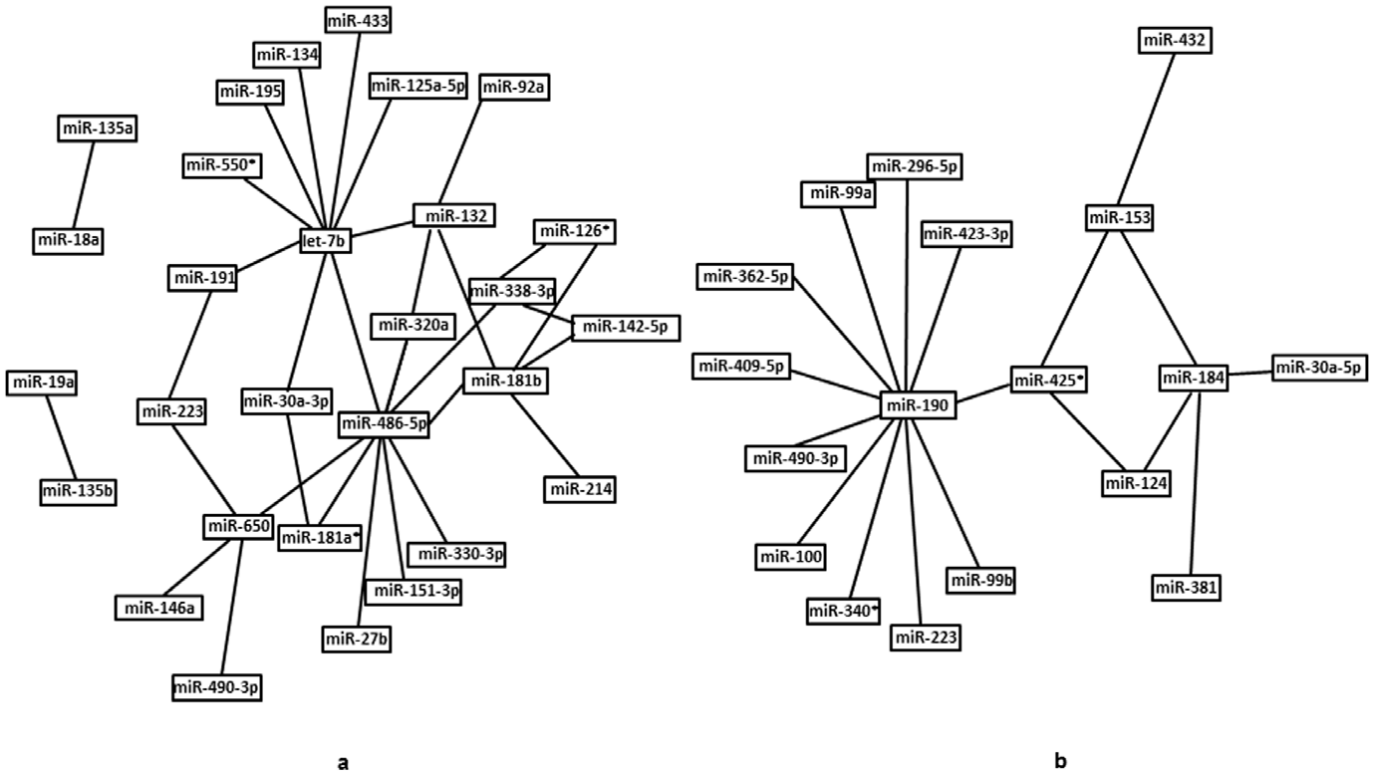
Network of microRNAs pairs in normal control and depressed groups. **A**. Network of microRNA pairs that showed no significant correlation in the normal control group yet *did* exhibit a significant positive correlation in the depressed suicide group. See text for details. **B**. Network of microRNA pairs that showed significant positive correlations in the normal control group yet were *not* correlated in the depressed suicide group. See text for details.

This procedure revealed a set of 29 miRNAs, none of whom were pairwise correlated in the normal control group, but which formed a very extensive inter-connected network in the depressed group (**Fig. 3a**). Several of the miRNAs (let-7b, mir-132, 181b, 338-3p, 486-5p, and 650) were “hubs” correlated with four to nine other miRNAs in the network. Only two of the miRNAs were significantly down-regulated in the depressed group (mir-142-5p and 146a, neither of them hubs). Of the network miRNAs, mir-181b was reported to be upregulated in schizophrenia [14], and mir-132 and 134 are known to be CREB-regulated and involved in neurite growth and dendritic spine growth, respectively [41], [42]. Thus, correlation analysis revealed the existence of factors that drive co-expression of a group of miRNAs across individuals specifically in the depressed group (**Fig. 3a**). A similar analysis also revealed a smaller set of miRNAs that were pairwise correlated in the control group but NOT in the depressed suicide group (**Fig. 3b**).

### Expression of dicer, drosha and DGCR8 mRNA and selected pri-miRs

Three genes involved in miRNA biogenesis (dicer, drosha and DGCR8) were measured in the entire cohort using RT-PCR, using the same total RNA employed for the miRNA plate measurements (**Table 4**). No significant difference was observed between the control and depressed groups for any of these mRNAs (**Table 4**). Two of the significantly down-regulated miRNAs were also examined, that are encoded by single primary transcripts at intergenic loci. Pri-miR-155 levels showed a 19% decrease in the depressed group, but the finding was not statistically significant (p = 0.30; **Table 4**). Pri-miR-494 showed a 18% increase in the depressed group, but this did not achieve significance either (p = 0.088).

**Table 4:** Levels of Dicer, Drosha and DGCR8 mRNA are similar in depressed suicide subjects and normal controls.

	Experiment 1		Experiment 2	
	% control mean	p-value	% control mean	p-value
**Dicer**	78	0.26	107	0.67
**Drosha**	90	0.62	88	0.18
**DGCR8**	106	0.75	97	0.76
**Pri-miR-155**		81	0.3	
**Pri-miR-494**		118	0.088	

Experiment 1: RT step carried out using random hexanucleotide primers. Experiment 2: RT step carried out using gene specific primers. Data were normalized using U44, and p-values were calculated using t-test, 2-tailed, unpaired. None of the comparisons were statistically significant at p = 0.05 (paired test gave similar results).

## Discussion

This study was designed to examine whether miRNA expression is altered in postmortem prefrontal cortex of depressed suicide subjects compared to matched control subjects who died from natural or accidental causes. We observed that miRNA expression was globally down-regulated in depressed suicide subjects, by 17% on average, a finding that has extremely high statistical significance. Global miRNA alterations have been reported in other neuropsychiatric conditions. Notably, Beveridge et al. [14], [15] reported that overall miRNA expression is *elevated* to a similar extent in samples of postmortem superior temporal and prefrontal cortex in patients with schizophrenia. The elevated miRNA expression was correlated with up-regulation of dicer, drosha, and especially DGCR8 mRNA levels [15], [43], although these findings have not yet been replicated by other groups. Sadikovic has emphasized that the RNAs commonly used to normalize miRNA data (U6, U44 and U48) are very sensitive to post-mortem decay and must be carefully matched across groups to avoid the artifactual appearance of global shifts in miRNA expression [44]. This potential artifact was ruled out in the present study, because U6 used as normalizer showed equal expression and low variance in both groups. Moreover, the use of spike-in synthetic RNAs in our samples confirmed the global down-regulation.

Such changes in miRNA levels are unlikely to be produced by alterations in tissue composition or changes in cellular compartments (e.g. loss of glial cells, neuronal shrinkage, or loss of dendritic spines). At the molecular level, these findings might be explained by one or more of the following responses: a) decreased transcriptional or epigenetic repression of primary miRNA gene transcripts, b) alterations in miRNA processing (e.g., by dicer, phospho-TRBP, drosha, DGCR8, etc.), c) alterations in level of Argonaute homolog proteins, or d) accelerated turnover of miRNAs. Altered miRNA processing has been described in various forms of cancer and in several cases, levels of dicer, drosha correlate with tumor type and clinical outcome [45], [46]. Changes in Argonaute protein levels can also cause global changes in miRNA expression [41]. Because our data do not show significant changes in mRNA levels of dicer, drosha or DGCR8, and we did not observe significant changes in several selected pri-miRs, further studies are needed to understand the molecular mechanisms involved in the current situation.

As well, the functional implications of global miRNA shifts have not been well studied and may vary depending on the biological context. For example, if RISC binding sites are limiting for gene targeting, then individual miRNAs will compete among themselves for RISC binding sites and hence for regulating their targets. A uniform decrease in abundance of the entire miRNA population may, if large enough, create a new situation in which RISC binding sites are now in excess. This might render each miRNA molecule more effective, despite their overall reduced numbers.

Both the global decrease of miRNA expression and its lower variability are consistent with hypo-activation of the frontal cortex, which has been reported in depressed subjects [47], [48], [49], [50]. In a previous study, we found a global increase in miRNA expression that accompanied learning in inbred C57BL/6J mice [19]. The miRNAs which showed the largest changes across groups tended to show high inter-individual variability within groups (both in the discrimination learning group and in the pseudo-training control group). This suggests that the inter-individual variability of miRNA expression values is not simply random noise, but reflects the fact that at least some miRNAs are biologically responsive to a variety of environmental, sensory and/or contextual cues even in the control animals. It is pertinent to mention that in a recent preliminary study, we found that the expression of miRNAs is downregulated in frontal cortex of rats treated with corticosterone (Dwivedi et al., unpublished data). Some of these miRNAs were similar to those observed in prefrontal cortex of depressed suicide subjects, suggesting the possibility that stress might be one of the factors regulating expression of miRNAs in depressed subjects.

Among targets that have been experimentally validated (**Table 2**) and that are co-expressed in prefrontal cortex, at least three (VEGFA, BCL2, and DNA methyltransferase 3B) have been previously implicated in depression or suicide [51], [52], [53]. Of miRNAs that showed the largest and most robust changes in depressed subjects, predicted targets included several brain-active transcription factors such as MITF, SMAD5, MYCN, estrogen receptor alpha (ESR1) and BACH2. MITF and BACH2 transcription factor binding sites have been identified upstream of many brain-expressed miRNAs [40]. The splicing factor NOVA1 that regulates brain-specific alternative splicing was predicted to be hit by 4 different down-regulated miRNAs. Several presynaptic and postsynaptic proteins important for neurotransmission or synaptic plasticity were also predicted targets: VEGF receptor Neuropilin-1 (NRP1), GAP43, SNAP25, synaptojanin-1, synaptotagmin-1, and LIM kinase-1. VEGFA is a growth factor implicated in depression in both humans and animal models. VEGFA treatment upregulates nuclear expression of E2F1, another validated target, among other cell cycle-related genes [54]. In addition, a variety of signaling proteins, ion channels, and ubiquitin ligases were also identified. Several of these predicted targets have been linked to affective disorders. As well, several of the down-regulated miRNAs may also interact with targets in a variety of unconventional ways [55], [56]: for example, mir-10a can enhance translation of 5′-TOP proteins responsible for regulating global translation of transcripts [57] and mir-324-5p lies upstream of the nearby PSD95 gene and may affect its transcription [58].

The second major finding of our study is that miRNA pathways are not simply down-regulated in the depressed group, but are also dramatically *reorganized* in a coordinated fashion. A set of 29 miRNAs was identified whose expression was not correlated at all with each other in the control group, but which formed a highly correlated network in the depressed group (**Fig. 3a**). Most of these miRNAs showed no significant change in mean expression levels across groups. Such a network is likely to reflect the influence of shared factor(s) which regulate the miRNAs in depressed suicide subjects, e.g., transcription factors or proteins involved in the miRNA processing pathway that regulate specific subsets of miRNAs [59].

The miRNA co-expression analysis complemented our separate analysis of how miRNA levels correlated with protein abundance of their predicted targets (DMNT3b, VEGFA BCL2) across individuals. There was no simple global change in target abundance – rather, DMNT3b was strongly up-regulated in the depressed suicide group, whereas BCL2 was down-regulated and VEGFA was unchanged. Several miRNAs showed a strong positive correlation with DMNT3b and BCL2, instead of the inverse correlation that would be expected if the miRNAs simply inhibited translation of their protein targets. These miRNAs appear to be co-regulated with their targets, a phenomenon that is commonly observed in brain [7] and that, again, emphasizes the importance of considering the entire miRNA – mRNA network and cellular context when interpreting the likely effects of changes in miRNA abundance.

Preliminary studies in our laboratory have measured miRNA expression in prefrontal cortex (BA10) postmortem samples provided by the Stanley Neuropathology Consortium, consisting of 15 controls, 15 depressed, 15 bipolar and 15 schizophrenic subjects. About half of the psychiatric patients died of suicide across the diagnostic categories. We observed a significant global down-regulation of miRNA expression when all suicide (n = 20) vs. all non-suicide (n = 40) subjects are compared regardless of diagnosis (ms., in preparation).

In conclusion, measurements of miRNAs can substantially contribute to our understanding of how gene expression networks are reorganized in depression and/or suicide. We do not know if specific miRNAs are independent risk factors or if the down-regulation of miRNAs precedes the onset of symptomatology, yet the network of down-regulated miRNAs certainly regulates genes that participate in pathogenesis. The present report is a discovery study that needs replication, and in the future, it will be interesting to examine the cell types and subcellular distribution of the affected miRNAs, additional brain regions, and other types of mood disorders.

## Supporting Information

File S1 **This file contains Ct values for all 384 measured miRNAs.** Ct values were U6-normalized, truncated at the detection threshold of Ct = 35, and placed in order of RT-PCR processing.
(PDF)

## References

[1] Dwivedi Y (2009). Brain-derived neurotrophic factor: role in depression and suicide.. Neuropsychiatr Dis Treat.

[2] Duman RS (2002). Pathophysiology of depression: the concept of synaptic plasticity.. Eur Psychiatry.

[3] Bartel DP (2009). MicroRNAs: target recognition and regulatory functions.. Cell.

[4] Bushati N, Cohen SM (2007). microRNA functions.. Annu Rev Cell Dev Biol.

[5] Vasudevan S, Tong Y, Steitz JA (2007). Switching from repression to activation: microRNAs can up-regulate translation.. Science.

[6] Kim DH, Saetrom P, Snøve O, Rossi JJ (2008). MicroRNA-directed transcriptional gene silencing in mammalian cells.. Proc Natl Acad Sci USA.

[7] Tsang J, Zhu J, van Oudenaarden A (2007). MicroRNA-mediated feedback and feedforward loops are recurrent network motifs in mammals.. Mol Cell.

[8] Packer AN, Xing Y, Harper SQ, Jones L, Davidson BL (2008). The bifunctional microRNA miR-9/miR-9* regulates REST and CoREST and is downregulated in Huntington's disease.. J Neurosci.

[9] Johnson R, Zuccato C, Belyaev ND, Guest DJ, Cattaneo E (2008). A microRNA-based gene dysregulation pathway in Huntington's disease.. Neurobiol Dis.

[10] Kim J, Inoue K, Ishii J, Vanti WB, Voronov SV (2007). A MicroRNA feedback circuit in midbrain dopamine neurons.. Science.

[11] Wang G, van der Walt JM, Mayhew G, Li YJ (2008). Variation in the miRNA-433 binding site of FGF20 confers risk for Parkinson disease by overexpression of alpha-synuclein.. Am J Hum Genet.

[12] Abelson JF, Kwan KY, O'Roak BJ, Baek DY, Stillman AA (2005). Sequence variants in SLITRK1 are associated with Tourette's syndrome.. Science.

[13] Perkins DO, Jeffries CD, Jarskog LF, Thomson JM, Woods K (2007). microRNA expression in the prefrontal cortex of individuals with schizophrenia and schizoaffective disorder.. Genome Biol.

[14] Beveridge NJ, Tooney PA, Carroll AP, Gardiner E, Bowden N (2008). Dysregulation of miRNA 181b in the temporal cortex in schizophrenia.. Hum Mol Genet.

[15] Beveridge NJ, Gardiner E, Carroll AP, Tooney PA, Cairns MJ (2010). Schizophrenia is associated with an increase in cortical microRNA biogenesis.. Mol Psychiatry.

[16] Kim AH, Reimers M, Maher B, Williamson V, McMichael O (2010). MicroRNA expression profiling in the prefrontal cortex of individuals affected with schizophrenia and bipolar disorders.. Schizophr Res.

[17] Hansen T, Olsen L, Lindow M, Jakobsen KD, Ullum H (2007). Brain expressed microRNAs implicated in schizophrenia etiology.. PLoS ONE.

[18] Hunsberger JG, Austin DR, Chen G, Manji HK (2009). MicroRNAs in Mental Health: From Biological Underpinnings to Potential Therapies.. Neuromolecular Med.

[19] Smalheiser NR, Lugli G, Lenon AL, Davis JM, Torvik VI (2010). Olfactory discrimination training up-regulates and reorganizes expression of microRNAs in adult mouse hippocampus.. ASN Neuro.

[20] Smalheiser NR, Lugli G, Rizavi HS, Zhang H, Torvik VI (2011). MicroRNA expression in rat brain exposed to repeated inescapable shock: differential alterations in learned helplessness vs. non-learned helplessness.. Int J Neuropsychopharmacol.

[21] Dwivedi Y, Pandey GN (2011). Elucidating biological risk factors in suicide: Role of protein kinase A.. Prog Neuropsychopharmacol Biol Psychiatry.

[22] Dwivedi Y (2006). The concept of dysregulated signal transduction and gene expression in the pathophysiology of mood disorders.. Current Psychiatry Reviews.

[23] Haines D (2000). Neuroanatomy: An Atlas of Structures, Sections, and Systems. 5th ed.

[24] Harrison PJ, Heath PR, Eastwood SL, Burnet PW, McDonald B (1995). The relative importance of premortem acidosis and postmortem interval for human brain gene expression studies: selective mRNA vulnerability and comparison with their encoded proteins.. Neurosci Lett.

[25] Conner KR, Conwell Y, Duberstein PR (2001). The validity of proxy-based data in suicide research: a study of patients 50 years of age and older who attempted suicide. II. Life events, social support and suicidal behavior.. Acta Psychiatr Scand.

[26] Kelly TM, Mann JJ (1996). Validity of DSM-III-R diagnosis by psychological autopsy: a comparison with clinican ante-mortem diagnosis.. Acta Psychiatr Scand.

[27] Lesage AD, Boyer R, Grunberg F, Vanier C, Morissette R (1994). Suicide and mental disorders: a case-control study of young men.. Am J Psychiatry.

[28] Spitzer RL, Williams JB, Gibbon M, First MB (1992). The structural clinical interview for DSM-III-R (SCID). I. History, rationale, and description.. Arch Gen Psychiatry.

[29] Lugli G, Torvik VI, Larson J, Smalheiser NR (2008). Expression of microRNAs and their precursors in synaptic fractions of adult mouse forebrain.. J Neurochem.

[30] Dwivedi Y, Rizavi HS, Zhang H, Roberts RC, Conley RR (2009). Aberrant extracellular signal-regulated kinase (ERK)1/2 signaling in suicide brain: role of ERK kinase 1 (MEK1).. Int J Neuropsychopharmacol.

[31] Hardin J, Wilson J (2009). A note on oligonucleotide expression values not being normally distributed.. Biostatistics.

[32] Landgraf P, Rusu M, Sheridan R, Sewer A, Iovino N (2007). A mammalian microRNA expression atlas based on small RNA library sequencing.. Cell.

[33] Watanabe Y, Tomita M, Kanai A (2007). Computational methods for microRNA target prediction.. Methods Enzymol.

[34] Zhu Y, Jin K, Greenberg DA (2003). Vascular endothelial growth factor promotes proliferation of cortical neuron precursors by regulating E2F expression.. FASEB J.

[35] Saini HK, Enright AJ, Griffiths-Jones S (2008). Annotation of Mammalian Primary microRNAs.. BMC Genomics.

[36] Friedman RC, Farh KK, Burge CB, Bartel DP (2009). Most mammalian mRNAs are conserved targets of microRNAs.. Genome Res.

[37] Smalheiser NR, Torvik VI (2006). Alu elements within human mRNAs are probable microRNA targets.. Trends in Genetics.

[38] Xiao F, Zuo Z, Cai G, Kang S, Gao X (2009). miRecords: an integrated resource for microRNA-target interactions.. Nucleic Acids Res.

[39] Wang J, Cheng CM, Zhou J, Smith A, Weickert CS (2004). Estradiol alters transcription factor gene expression in primate prefrontal cortex.. J Neurosci Res.

[40] Wu J, Xie X (2006). Comparative sequence analysis reveals an intricate network among REST, CREB and miRNA in mediating neuronal gene expression.. Genome Biol.

[41] Wayman GA, Davare M, Ando H, Fortin D, Varlamova O (2008). An activity-regulated microRNA controls dendritic plasticity by down-regulating p250GAP.. Proc Natl Acad Sci U S A.

[42] Schratt GM, Tuebing F, Nigh EA, Kane CG, Sabatini ME (2006). A brain-specific microRNA regulates dendritic spine development.. Nature.

[43] Santarelli DM, Beveridge NJ, Tooney PA, Cairns MJ (2011). Upregulation of dicer and microRNA expression in the dorsolateral prefrontal cortex Brodmann area 46 in schizophrenia.. Biol Psychiatry.

[44] Sadikovic B, Pearson R, Meng L, Beaudet A (2011). Identification of miRNAs and target mRNAs with degulated expression in schizophrenia and bipolar brains..

[45] Merritt WM, Lin YG, Han LY, Kamat AA, Spannuth WA (2008). Dicer, Drosha, and outcomes in patients with ovarian cancer.. N Engl J Med.

[46] O'Carroll D, Mecklenbrauker I, Das PP, Santana A, Koenig U (2007). A Slicer-independent role for Argonaute 2 in hematopoiesis and the microRNA pathway.. Genes Dev.

[47] Covington HE, Lobo MK, Maze I, Vialou V, Hyman JM (2010). Antidepressant effect of optogenetic stimulation of the medial prefrontal cortex.. J Neurosci.

[48] Flor-Henry P, Lind JC, Koles ZJ (2004). A source-imaging (low-resolution electromagnetic tomography) study of the EEGs from unmedicated males with depression.. Psychiatry Res.

[49] Werner NS, Meindl T, Materne J, Engel RR, Huber D (2009). Functional MRI study of memory-related brain regions in patients with depressive disorder.. J Affect Disorder.

[50] Townsend JD, Eberhart NK, Bookheimer SY, Eisenberger NI, Foland-Ross LC (2010). fMRI activation in the amygdala and the orbitofrontal cortex in unmedicated subjects with major depressive disorder.. Psychiatry Res.

[51] Takebayashi M, Hashimoto R, Hisaoka K, Tsuchioka M, Kunugi H (2010). Plasma levels of vascular endothelial growth factor and fibroblast growth factor 2 in patients with major depressive disorders.. J Neural Transm.

[52] Poulter MO, Du L, Weaver IC, Palkovits M, Faludi G (2008). GABAA receptor promoter hypermethylation in suicide brain: implications for the involvement of epigenetic processes.. Biol Psychiatry.

[53] Tohda M, Mingmalairak S, Murakami Y, Matsumoto K (2010). Enhanced expression of BCL2/adenovirus EIB 19-kDa-interacting protein 3 mRNA, a candidate for intrinsic depression-related factor, and effects of imipramine in the frontal cortex of stressed mice.. Biol Pharm Bull.

[54] Zhu Y, Jin K, Mao XO, Greenberg DA (2003). Vascular endothelial growth factor promotes proliferation of cortical neuron precursors by regulating E2F expression.. FASEB J.

[55] Filipowicz W, Bhattacharyya SN, Sonenberg N (2008). Mechanisms of post-transcriptional regulation by microRNAs: are the answers in sight?. Nat Rev Genet.

[56] Bartel DP (2009). MicroRNAs: target recognition and regulatory functions.. Cell.

[57] Ørom UA, Nielsen FC, Lund AH (2008). MicroRNA-10a binds the 5′UTR of ribosomal protein mRNAs and enhances their translation.. Mol Cell.

[58] Smalheiser NR (2003). EST analyses predict the existence of a population of chimeric microRNA precursor-mRNA transcripts expressed in normal human and mouse tissues.. Genome Biol.

[59] Trabucchi M, Briata P, Garcia-Mayoral M, Haase AD, Filipowicz W (2009). The RNA-binding protein KSRP promotes the biogenesis of a subset of microRNAs.. Nature.

