# Supplementary material for: MicroRNA Expression Is Down-Regulated and Reorganized in Prefrontal Cortex of Depressed Suicide Subjects
Source: PLoS One. 2012 Mar 9;7(3):e33201. doi: 10.1371/journal.pone.0033201 (PMC3302855; doi:10.1371/journal.pone.0033201)
Supplement: File S1 — This file contains Ct values for all 384 measured miRNAs. Ct values were U6-normalized, truncated at the detection threshold of Ct = 35, and placed in order of RT-PCR processing. (PDF) [file pone.0033201.s001.pdf]

Supplementary Table 1

Supplementary Table 1

U6-normalized and truncated at Ct=35, Depression Group placed in order of RT-PCR processing

(paired across groups)		1	2	3	4	5	6	7	8	9	10
1	hsa-let-7a-4373169	24.61	24.85	25.69	24.71	23.96	24.85	25.80	25.16	25.87	24.80
2	hsa-let-7b-4373168	22.99	23.58	23.53	22.99	21.97	23.25	24.16	24.39	24.34	23.13
3	hsa-let-7c-4373167	23.13	23.84	25.01	23.29	23.23	23.77	24.42	24.31	24.71	23.78
4	hsa-let-7d-4373166	26.09	26.45	27.61	26.19	25.97	26.91	27.53	27.41	27.86	27.11
5	hsa-let-7e-4373165	27.70	28.07	29.36	28.14	27.44	28.08	28.09	29.27	29.06	28.68
6	hsa-let-7g-4373163	23.34	23.52	24.59	23.27	22.77	23.67	23.94	23.92	24.37	23.39
7	hsa-miR-10a-4373153	29.33	31.91	32.81	32.02	33.11	31.97	31.78	33.92	33.65	33.63
8	hsa-miR-10b-4373152	35.00	35.00	35.00	35.00	35.00	35.00	35.00	35.00	35.00	35.00
9	hsa-miR-17-3p-4373120	30.71	31.04	31.03	31.56	30.25	31.96	31.27	33.21	32.67	32.66
10	hsa-miR-17-5p-4373119	29.37	29.47	30.38	29.81	29.75	30.02	29.93	29.57	30.41	28.84
12	RNU44-4373384	24.20	24.98	26.02	24.54	25.07	25.35	25.30	25.54	25.23	24.39
13	hsa-miR-18a-4373118	31.04	30.66	30.53	31.04	29.88	31.28	30.84	31.42	31.44	30.78
14	hsa-miR-19a-4373099	27.36	27.22	26.86	26.60	25.68	26.84	26.21	26.27	26.72	25.44
15	hsa-miR-19b-4373098	24.01	24.29	24.05	24.05	22.79	24.09	23.70	23.70	24.31	23.50
16	hsa-miR-21-4373090	26.55	26.38	26.26	26.59	25.17	26.56	26.80	27.16	27.69	26.76
17	hsa-miR-24-4373072	22.38	22.75	23.28	22.45	22.03	23.00	23.00	22.94	23.36	22.72
18	hsa-miR-34a-4373278	29.04	29.47	29.95	28.97	29.38	30.09	29.99	29.59	30.11	30.60
19	hsa-miR-34b-4373037	33.43	32.23	32.26	33.83	31.51	32.94	32.07	33.76	33.65	33.57
20	hsa-miR-34c-4373036	32.07	31.85	32.29	33.81	30.63	33.45	31.67	32.29	35.00	33.97
21	hsa-miR-92-4373013	23.77	24.00	23.86	24.08	22.62	24.22	24.06	24.80	25.22	23.68
22	hsa-miR-93-4373012	24.47	25.13	25.77	24.84	24.32	25.36	25.04	25.22	25.62	25.02
23	hsa-miR-107-4373154	27.40	28.40	29.86	27.62	27.72	28.32	28.30	28.26	28.79	28.82
24	hsa-miR-141-4373137	35.00	35.00	35.00	35.00	34.20	35.00	35.00	33.70	35.00	35.00
25	hsa-miR-142-3p-4373136	26.05	26.97	27.26	26.34	25.63	25.91	25.00	25.84	26.00	27.81
26	hsa-miR-142-5p-4373135	28.54	30.14	27.19	29.03	29.06	29.64	29.54	29.68	30.38	29.71
27	hsa-miR-146a-4373132	26.60	26.97	27.82	26.45	27.08	27.17	26.80	26.27	27.18	27.02
28	hsa-miR-155-4373124	28.75	30.75	30.91	30.29	29.85	30.20	29.39	29.01	30.10	35.00
29	hsa-miR-181c-4373115	28.48	29.11	29.55	29.52	28.24	29.37	28.42	29.43	30.33	29.29
30	hsa-miR-191-4373109	23.12	23.61	24.60	23.64	23.33	24.08	23.69	23.41	23.74	22.93
31	hsa-miR-200c-4373096	31.11	30.49	32.03	31.17	30.98	31.47	30.14	31.09	30.28	30.32
32	hsa-miR-215-4373084	35.00	35.00	35.00	35.00	33.64	35.00	35.00	35.00	35.00	35.00
33	hsa-miR-218-4373081	23.30	23.85	25.41	23.67	23.48	23.92	21.96	23.39	23.73	25.32
34	hsa-miR-221-4373077	23.71	24.12	25.77	23.50	23.81	24.15	24.73	24.75	25.50	25.41
35	hsa-miR-223-4373075	24.03	24.24	25.12	24.46	23.73	24.14	24.19	24.17	25.10	24.18
36	hsa-miR-301-4373064	26.56	27.08	27.52	26.62	26.00	26.84	26.40	26.25	26.88	28.06
37	hsa-miR-345-4373039	27.09	27.80	28.38	27.88	26.92	28.36	28.27	29.22	28.84	29.52
38	hsa-miR-372-4373029	30.63	35.00	35.00	35.00	35.00	35.00	35.00	35.00	35.00	35.00
39	hsa-miR-375-4373027	29.35	30.89	31.30	29.53	30.70	30.00	31.23	32.80	31.77	30.61
40	hsa-miR-378-4373024	29.86	30.38	31.79	30.56	30.17	30.91	31.98	31.76	31.95	33.43
41	hsa-miR-20a-4373286	25.09	25.22	25.48	25.47	24.12	25.45	24.84	25.13	25.84	25.05
42	hsa-miR-9-4373285	19.49	19.52	21.35	19.40	19.46	19.96	20.24	20.04	20.68	21.13
43	hsa-miR-137-4373174	22.12	22.40	24.31		22.20	22.14	21.96	21.88	22.40	23.35
44	hsa-miR-146b-4373178	24.59	24.97	26.74		25.04	25.09	24.61	24.45	25.07	25.12
45	hsa-miR-181b-4373116	23.13	23.21	22.87	23.61	22.03	23.65	23.59	23.65	24.19	22.74
46	hsa-miR-181d-4373180	24.38	24.38	25.17	24.34	23.92	24.98	24.65	24.87	25.41	25.12
47	hsa-miR-200a-4373273	35.00	35.00	35.00	33.60	35.00	32.55	32.17	35.00	35.00	35.00
48	hsa-miR-20b-4373263	29.58	29.23	30.41	28.13	28.53	28.91	29.08	28.51	29.68	29.34
49	hsa-miR-1-4373161	27.95	28.62	30.05	28.20	27.87	28.02	28.17	28.06	28.18	28.11
50	hsa-miR-26a-4373070	19.26	20.00	20.54	19.52	19.07	19.99	19.91	19.88	20.26	19.59
51	hsa-miR-26b-4373069	23.25	23.66	24.01	23.03	22.41	23.50	22.98	23.89	24.35	23.85

Supplementary Table 1

52	hsa-miR-27a-4373287	25.48	25.61	26.86	25.53	25.16	26.24	25.81	24.98	26.03	26.70
53	hsa-miR-27b-4373068	25.08	25.25	25.20	25.22	23.99	25.44	25.12	24.91	25.67	25.41
54	hsa-miR-103-4373158	23.21	23.70	25.08	23.45	23.24	23.79	23.99	23.71	24.73	24.62
55	hsa-miR-125a-4373149	21.98	22.47	23.50	22.29	22.28	23.06	22.89	22.39	23.09	22.77
56	hsa-miR-125b-4373148	19.98	20.53	21.54	20.48	20.09	20.97	21.23	20.75	21.16	20.38
57	hsa-miR-152-4373126	26.61	27.75	27.91	27.73	26.61	27.89	27.44	27.52	28.06	27.15
58	hsa-miR-183-4373114	35.00	34.65	35.00	35.00	35.00	35.00	35.00	34.36	35.00	35.00
61	hsa-miR-210-4373089	28.32	28.62	29.18	29.19	27.48	28.38	28.43	28.88	29.40	29.14
62	hsa-miR-222-4373076	21.59	22.35	24.02	22.02	22.14	22.36	23.12	22.97	23.39	23.30
63	hsa-miR-296-4373066	30.00	30.22	30.80	31.19	30.60	30.53	30.92	31.91	32.50	31.12
64	hsa-miR-302a-4373275	35.00	35.00	35.00	35.00	35.00	35.00	35.00	35.00	35.00	35.00
65	hsa-miR-302c-4373277	35.00	35.00	35.00	35.00	35.00	35.00	35.00	35.00	35.00	35.00
66	hsa-miR-302d-4373063	35.00	35.00	35.00	33.99	35.00	35.00	33.63	35.00	33.91	33.34
67	hsa-miR-324-5p-4373052	26.00	26.03	27.51	27.45	25.77	26.36	25.88	25.69	26.47	28.60
68	hsa-miR-367-4373034	33.54	33.09	34.94	35.00	35.00	35.00	35.00	32.89	35.00	35.00
69	hsa-miR-423-4373015	27.10	27.58	28.20	27.51	27.02	28.12	28.65	28.81	29.28	27.74
70	hsa-miR-324-3p-4373053	26.38	26.62	27.44	26.68	26.35	27.05	26.79	27.00	27.41	26.61
71	hsa-miR-369-5p-4373195	30.23	30.88	32.53	30.63	30.53	32.16	31.00	31.19	32.10	32.72
72	hsa-miR-449-4373207	31.26	31.37	34.94	32.03	32.74	32.86	32.91	33.53	32.80	35.00
73	hsa-miR-497-4373222	26.88	27.39	28.34	27.30	27.12	27.87	27.38	27.11	27.53	28.32
74	hsa-miR-501-4373226	26.21	29.32	29.67	30.04	27.46	30.28	30.74	30.48	28.21	31.03
75	hsa-miR-509-4373234	33.17	32.67	32.21	31.97	35.00	31.60	32.18	35.00	33.70	31.52
76	hsa-miR-510-4373235	33.99	34.51	35.00	35.00	35.00	35.00	35.00	35.00	35.00	35.00
77	hsa-miR-511-4373236	30.77	33.64	35.00	32.36	31.69	30.37	34.81	35.00	32.78	31.60
78	hsa-miR-514-4373240	34.29	35.00	35.00	33.49	35.00	35.00	35.00	33.60	35.00	35.00
79	hsa-miR-515-3p-4373241	34.89	33.67	35.00	35.00	33.72	35.00	34.56	35.00	33.91	35.00
80	hsa-miR-515-5p-4373242	35.00	35.00	35.00	35.00	35.00	35.00	35.00	35.00	35.00	35.00
81	hsa-miR-517a-4373243	31.91	35.00	35.00	35.00	35.00	35.00	30.18	34.75	35.00	35.00
82	hsa-miR-517b-4373244	35.00	35.00	35.00	35.00	35.00	35.00	35.00	35.00	35.00	35.00
83	hsa-miR-517c-4373264	31.66	32.24	32.97	31.71	32.45	31.79	31.69	30.33	32.75	32.79
84	hsa-miR-518a-4373186	35.00	35.00	35.00	35.00	35.00	35.00	35.00	35.00	35.00	35.00
85	hsa-miR-518b-4373246	32.82	34.31	34.14	33.99	33.11	33.86	34.18	33.42	34.58	35.00
86	hsa-miR-518c-4373247	35.00	34.13	35.00	35.00	35.00	35.00	33.55	35.00	35.00	35.00
87	hsa-miR-518d-4373248	33.54	33.41	35.00	32.49	32.74	34.53	35.00	34.72	35.00	34.70
88	hsa-miR-518e-4373265	35.00	35.00	35.00	35.00	35.00	35.00	35.00	35.00	35.00	34.23
89	hsa-miR-520a-4373268	35.00	35.00	35.00	35.00	35.00	35.00	35.00	35.00	35.00	35.00
90	hsa-miR-520b-4373252	35.00	35.00	35.00	34.65	35.00	35.00	35.00	35.00	35.00	35.00
91	hsa-miR-520c-4373253	35.00	35.00	35.00	35.00	35.00	35.00	34.42	35.00	35.00	35.00
92	hsa-miR-520d-4373254	35.00	35.00	35.00	35.00	35.00	35.00	35.00	35.00	35.00	35.00
93	hsa-miR-520e-4373255	35.00	35.00	35.00	35.00	35.00	35.00	35.00	35.00	35.00	35.00
94	hsa-miR-520f-4373256	34.42	35.00	35.00	35.00	35.00	35.00	34.69	35.00	35.00	35.00
95	hsa-miR-520g-4373257	32.78	35.00	35.00	35.00	34.60	35.00	35.00	35.00	34.10	35.00
96	hsa-miR-520h-4373258	35.00	34.09	35.00	34.40	35.00	35.00	35.00	35.00	35.00	35.00
97	hsa-miR-30a-3p-4373062	25.39	25.83	26.92	25.65	25.70	26.46	26.34	25.76	25.92	26.14
98	hsa-miR-30a-5p-4373061	22.75	23.02	24.18	22.79	22.48	23.09	22.58	22.06	22.83	24.25
99	hsa-miR-30c-4373060	22.05	22.27	23.57	22.11	22.13	22.46	23.37	22.82	23.51	24.30
100	hsa-miR-30d-4373059	24.24	24.67	25.72	24.73	24.21	24.99	24.35	24.18	24.57	25.69
101	hsa-miR-30e-3p-4373057	25.54	25.84	26.83	25.53	25.25	26.05	26.22	25.81	26.40	26.18
102	hsa-miR-95-4373011	25.48	25.98	27.30	25.59	25.63	25.95	25.87	25.19	26.02	26.02
103	hsa-miR-100-4373160	22.65	22.79	23.27	23.07	22.15	23.25	23.45	23.67	23.80	22.89
104	hsa-miR-101-4373159	26.69	27.57	27.75	26.96	26.13	26.83	26.59	26.55	26.89	27.82
105	hsa-miR-126-4373269	24.99	25.51	26.49	24.73	24.70	24.91	24.77	24.51	25.44	24.62
106	hsa-miR-127-4373147	22.73	23.33	24.60	22.94	23.19	23.49	23.92	23.74	23.90	23.98
109	hsa-miR-132-4373143	21.34	21.60	23.25	21.49	21.40	21.89	22.28	21.20	22.34	21.83
110	hsa-miR-133a-4373142	28.92	29.49	29.77	28.72	29.24	29.17	29.62	28.76	29.95	30.36

Supplementary Table 1

111	hsa-miR-135a-4373140	24.54	25.24	26.67	25.25	24.84	24.99	26.14	25.04	25.54	25.49
112	hsa-miR-135b-4373139	28.62	29.32	30.17	29.02	28.71	28.90	29.22	28.35	29.52	28.53
113	hsa-miR-148a-4373130	28.92	28.87	30.19	28.67	28.63	29.04	29.14	28.87	29.85	30.94
114	hsa-miR-148b-4373129	27.13	27.68	28.39	27.26	26.58	27.58	28.69	27.91	29.07	30.47
115	hsa-miR-184-4373113	30.41	30.04	32.16	29.66	30.32	31.25	30.48	34.67	30.89	30.16
116	hsa-miR-192-4373108	28.10	28.18	28.31	28.43	27.12	28.24	27.90	27.78	28.23	27.89
117	hsa-miR-194-4373106	29.13	29.38	29.56	28.97	28.27	29.79	28.73	28.77	30.00	29.05
118	hsa-miR-203-4373095	26.72	27.53	29.17	26.82	27.41	27.62	27.93	28.20	28.11	28.46
119	hsa-miR-204-4373094	24.48	24.49	25.67	24.84	24.49	25.38	24.93	24.47	25.89	25.41
120	hsa-miR-206-4373092	31.99	34.38	34.10	34.50	35.00	33.37	32.81	35.00	35.00	35.00
121	hsa-miR-211-4373088	29.80	30.80	31.59	33.67	33.54	33.72	33.62	32.62	29.81	29.48
122	hsa-miR-216-4373083	31.10	34.64	35.00	35.00	35.00	35.00	32.12	31.42	35.00	30.14
123	hsa-miR-217-4373082	35.00	34.37	35.00	35.00	35.00	35.00	35.00	35.00	35.00	35.00
124	hsa-miR-330-4373047	28.31	29.34	28.93	28.94	27.74	29.58	29.22	29.66	29.65	28.84
125	hsa-miR-335-4373045	25.83	26.42	27.56	25.67	25.75	26.13	26.14	25.60	26.49	26.75
126	hsa-miR-371-4373030	35.00	35.00	35.00	35.00	35.00	35.00	35.00	35.00	35.00	35.00
127	hsa-miR-379-4373023	26.41	26.73	28.28	26.01	26.16	26.56	26.60	26.30	26.66	27.00
128	hsa-miR-381-4373020	31.79	32.70	34.31	32.82	30.81	33.05	31.63	30.85	32.49	33.18
129	hsa-miR-383-4373018	26.25	26.61	28.17	26.10	26.60	27.02	26.80	26.04	26.93	26.88
130	hsa-miR-198-4373101	35.00	35.00	35.00	34.47	35.00	35.00	35.00	35.00	35.00	35.00
131	hsa-miR-30b-4373290	22.37	22.86	24.12	22.61	22.53	22.98	23.37	23.05	23.65	24.22
132	hsa-miR-133b-4373172	28.90	28.83	30.93	28.60	28.61	30.24	29.55	28.78	30.03	29.47
133	hsa-miR-224-4373187	32.52	33.63	35.00	31.90	35.00	32.98	34.68	33.29	35.00	32.79
134	hsa-miR-299-5p-4373188	27.61	28.44	29.99	27.85	28.16	28.45	29.37	28.94	29.42	30.83
135	hsa-miR-31-4373190	25.65	26.21	27.61	26.02	25.79	26.50	26.32	26.51	26.75	27.10
136	hsa-miR-409-5p-4373197	29.87	31.37	32.85	30.54	32.17	31.66	30.68	30.55	32.03	31.55
137	hsa-miR-432-4373280	25.90	26.58	27.91	25.96	25.95	26.49	26.59	26.52	27.21	26.48
138	hsa-miR-433-4373205	24.05	24.67	26.11	24.47	24.58	24.95	25.09	24.82	25.49	25.31
139	hsa-miR-485-5p-4373212	28.06	28.64	29.75	28.41	28.42	28.68	29.07	28.82	29.65	29.96
140	hsa-miR-489-4373214	31.42	33.41	34.28	34.25	32.80	31.78	33.12	31.43	34.20	32.70
141	hsa-miR-494-4373219	32.88	33.17	35.00	32.27	31.70	32.98	34.29	33.11	35.00	35.00
142	hsa-miR-506-4373231	35.00	35.00	35.00	35.00	35.00	35.00	35.00	35.00	35.00	35.00
143	hsa-miR-508-4373233	32.46	30.64	35.00	31.87	33.76	34.39	31.85	35.00	35.00	35.00
144	hsa-miR-521-4373259	35.00	35.00	35.00	35.00	35.00	35.00	35.00	35.00	35.00	35.00
145	hsa-miR-7-4373014	24.00	24.13	26.11	23.65	24.15	23.69	24.11	22.90	23.94	25.19
146	hsa-miR-23b-4373073	25.85	26.31	26.36	25.85	25.10	26.37	26.79	26.88	27.03	26.08
147	hsa-miR-28-4373067	27.91	28.30	29.01	27.80	27.48	28.30	28.36	27.94	28.87	28.41
148	hsa-miR-32-4373056	29.53	29.00	28.74	28.86	27.27	28.61	27.74	28.07	28.93	28.80
149	hsa-miR-134-4373141	27.96	28.33	29.64	27.79	27.71	28.23	29.27	27.88	28.65	28.32
150	hsa-miR-147-4373131	35.00	35.00	35.00	35.00	35.00	35.00	35.00	35.00	35.00	35.00
151	hsa-miR-149-4373128	23.06	23.72	25.26	23.47	23.79	23.95	25.13	24.47	25.17	24.52
152	hsa-miR-153-4373125	29.15	29.08	30.78	28.65	28.72	28.64	28.77	27.98	28.83	29.24
153	hsa-miR-186-4373112	25.67	25.92	27.00	25.84	25.33	26.06	26.27	25.80	26.70	26.43
154	hsa-miR-187-4373111	28.05	28.45	30.74	28.58	28.62	28.89	29.18	28.20	29.05	29.21
157	hsa-miR-190-4373110	29.58	29.66	30.22	29.06	28.10	28.76	28.96	28.51	29.05	29.24
158	hsa-miR-193a-4373107	31.58	31.26	32.13	31.56	30.61	31.48	31.63	31.36	32.04	33.20
159	hsa-miR-196a-4373104	35.00	35.00	35.00	35.00	35.00	35.00	35.00	35.00	35.00	35.00
160	hsa-miR-196b-4373103	35.00	35.00	35.00	35.00	35.00	33.76	35.00	35.00	35.00	35.00
161	hsa-miR-197-4373102	24.95	25.50	26.39	25.24	25.16	25.74	26.14	25.79	26.46	25.66
162	hsa-miR-205-4373093	35.00	35.00	35.00	34.54	35.00	35.00	35.00	35.00	35.00	35.00
163	hsa-miR-208-4373091	35.00	35.00	35.00	35.00	35.00	35.00	35.00	35.00	35.00	35.00
164	hsa-miR-213-4373086	28.42	28.39	28.68	28.62	27.77	28.88	28.63	28.20	29.04	27.68
165	hsa-miR-214-4373085	28.72	30.34	30.97	29.99	29.86	30.57	30.50	29.57	30.92	29.79
166	hsa-miR-220-4373078	35.00	35.00	35.00	35.00	35.00	35.00	35.00	35.00	35.00	35.00
167	hsa-miR-320-4373055	25.91	26.40	27.28	26.46	25.65	26.77	26.65	26.81	26.80	25.63

Supplementary Table 1

168	hsa-miR-325-4373051	35.00	35.00	35.00	35.00	35.00	35.00	35.00	35.00	35.00	35.00
169	hsa-miR-326-4373050	30.04	31.47	31.44	30.64	30.22	31.08	31.73	30.81	31.14	31.30
170	hsa-miR-328-4373049	23.23	24.15	25.17	23.93	23.74	23.84	25.57	25.39	26.05	25.55
171	hsa-miR-331-4373046	23.32	23.87	24.88	23.34	23.23	24.25	24.35	24.02	24.73	24.68
172	hsa-miR-337-4373044	34.32	35.00	35.00	35.00	34.01	35.00	35.00	35.00	35.00	35.00
173	hsa-miR-339-4373042	30.59	31.42	30.97	31.69	30.33	31.88	29.81	30.67	31.69	29.29
174	hsa-miR-340-4373041	26.35	27.26	27.85	26.89	26.30	27.40	27.20	27.20	27.44	26.61
175	hsa-miR-342-4373040	22.84	23.41	24.89	23.33	23.24	23.70	23.63	23.36	24.23	23.74
176	hsa-miR-361-4373035	25.97	26.90	27.69	26.44	26.46	27.07	27.13	26.85	27.34	26.84
177	hsa-miR-374-4373028	25.41	25.97	26.34	25.40	24.92	25.54	25.68	25.15	26.08	25.38
178	hsa-miR-380-3p-4373022	35.00	35.00	35.00	35.00	35.00	35.00	35.00	35.00	35.00	35.00
179	hsa-miR-422b-4373016	33.87	34.80	34.63	31.95	31.20	32.03	32.04	31.13	32.61	29.81
180	hsa-miR-98-4373009	26.89	27.71	28.78	26.95	26.65	27.14	27.90	27.65	28.35	27.41
181	hsa-miR-151-4373179	25.79	26.30	26.09	26.30	25.18	26.58	26.16	26.13	26.74	24.93
182	hsa-miR-182-4373271	31.59	33.12	33.14	33.66	30.82	32.03	31.61	31.67	34.22	32.13
183	hsa-miR-422a-4373200	34.43	34.51	35.00	33.26	32.78	34.06	34.66	33.20	34.44	34.15
184	hsa-miR-193b-4373185	31.51	31.45	31.62	31.55	30.72	32.03	33.40	32.64	33.65	31.54
185	hsa-miR-365-4373194	25.30	25.96	27.03	25.35	25.29	26.03	26.83	26.48	27.31	26.69
186	hsa-miR-425-4373202	27.52	27.61	29.04	27.93	27.80	28.62	28.41	28.06	28.73	27.74
187	hsa-miR-429-4373203	33.09	33.26	33.99	35.00	33.58	33.25	33.13	33.63	34.46	32.49
188	hsa-miR-491-4373216	26.12	26.68	28.18	26.47	26.55	27.02	26.87	26.60	26.85	27.30
189	hsa-miR-496-4373221	32.69	33.73	35.00	32.25	32.74	33.51	34.06	32.35	34.27	31.53
190	hsa-miR-500-4373225	31.38	31.97	33.81	31.86	33.62	33.41	31.92	35.00	33.68	33.78
191	hsa-miR-502-4373227	35.00	33.42	33.63	33.00	32.70	33.84	32.70	32.94	33.60	33.68
192	RNU6B-4373381	29.79	29.79	29.79	29.79	29.79	29.79	29.79	29.79	29.79	29.79
193	hsa-let-7f-4373164	26.65	27.40	28.38	26.68	26.02	26.64	26.88	27.42	27.67	26.54
194	hsa-miR-105-4373157	33.49	35.00	35.00	34.77	33.57	35.00	34.12	34.03	34.80	33.67
195	hsa-miR-106b-4373155	25.89	25.98	26.19	25.71	24.60	26.05	25.86	25.90	26.42	26.51
196	hsa-miR-122a-4373151	25.67	26.60	27.53	26.15	25.91	27.03	26.57	26.50	27.11	25.83
197	hsa-miR-124a-4373150	22.72	23.04	24.36	22.89	22.45	23.03	21.89	21.90	22.46	24.32
198	hsa-miR-126-4378064	21.71	22.36	23.64	22.18	22.08	22.46	22.43	21.90	22.55	22.43
199	hsa-miR-128b-4373170	28.57	29.16	31.25	28.88	29.67	29.34	30.73	29.93	31.66	30.07
200	hsa-miR-129-4373171	27.79	28.57	29.93	27.87	28.10	28.54	29.19	28.89	29.25	29.54
201	hsa-miR-130a-4373145	25.53	26.41	27.81	26.19	25.79	26.62	26.36	26.07	26.56	27.39
202	hsa-miR-130b-4373144	27.91	28.50	28.73	27.97	27.47	28.26	27.96	27.68	28.32	28.25
205	hsa-miR-139-4373176	26.91	27.13	29.04	26.70	27.52	27.35	28.80	27.92	29.36	28.32
206	hsa-miR-140-4373138	24.19	24.49	24.84	24.49	23.73	24.72	24.45	24.24	24.72	24.56
207	hsa-miR-143-4373134	28.98	30.26	30.90	29.45	29.74	30.12	29.06	29.02	29.80	30.39
208	hsa-miR-145-4373133	27.54	28.66	29.13	27.89	27.81	28.40	29.69	29.05	29.33	28.32
209	hsa-miR-16-4373121	21.71	21.94	22.35	21.64	21.11	21.98	21.76	21.65	22.45	21.41
210	hsa-miR-182-4378066	35.00	35.00	35.00	35.00	35.00	35.00	35.00	35.00	35.00	35.00
211	hsa-miR-185-4373181	34.21	33.67	33.94	35.00	35.00	33.79	34.51	35.00	33.36	33.30
212	hsa-miR-189-4378067	33.00	33.61	32.88	32.06	31.16	32.00	31.90	32.13	32.83	34.12
213	hsa-miR-18b-4373184	35.00	35.00	35.00	35.00	35.00	35.00	35.00	35.00	35.00	35.00
214	hsa-miR-195-4373105	23.57	23.99	25.08	23.74	23.54	24.07	23.96	23.89	24.08	23.76
215	hsa-miR-200a-4378069	32.41	31.54	31.50	31.72	30.27	31.62	31.64	31.92	32.56	31.93
216	hsa-miR-202-4378075	35.00	35.00	35.00	35.00	33.75	34.36		33.49	35.00	35.00
217	hsa-miR-202-4373274	35.00	35.00	35.00	35.00	35.00	35.00	35.00	35.00	35.00	35.00
218	hsa-miR-22-4373079	26.29	26.82	27.71	26.67	26.15	26.94	26.11	25.06	26.37	25.91
219	hsa-miR-23a-4373074	29.14	30.22	31.40	29.25	29.94	30.55	31.60	31.54	31.90	31.33
220	hsa-miR-299-3p-4373189	35.00	34.53	34.97	33.47	33.57	34.38	35.00	33.26	35.00	35.00
221	hsa-miR-29a-4373065	21.35	22.00	22.74	21.81	21.31	22.02	21.68	21.71	22.09	22.32
222	hsa-miR-29c-4373289	22.97	23.53	24.52	23.23	22.59	23.29	22.97	23.21	23.32	23.77
223	hsa-miR-302a-4378070	35.00	35.00	35.00	35.00	35.00	35.00	35.00	35.00	35.00	35.00
224	hsa-miR-302b-4378071	32.38	35.00	35.00	35.00	35.00	33.89	35.00	35.00	35.00	33.47

Supplementary Table 1

225	hsa-miR-302b-4373276	35.00	35.00	35.00	35.00	35.00	35.00	35.00	35.00	35.00	35.00
226	hsa-miR-302c-4378072	35.00	35.00	35.00	35.00	34.38	35.00	35.00	35.00	34.83	35.00
227	hsa-miR-329-4373191	34.08	33.11	34.46	32.38	31.87	32.99	34.90	32.98	33.75	35.00
228	hsa-miR-33-4373048	32.07	34.12	33.04	35.00	32.92	35.00	33.95	32.66	34.78	35.00
229	hsa-miR-362-4378092	30.21	30.25	31.43	30.39	30.33	31.14	31.23	31.07	31.71	30.51
230	hsa-miR-369-3p-4373032	33.78	33.11	33.75	32.37	32.81	33.39	31.65	32.23	34.28	35.00
231	hsa-miR-376a-4373026	28.31	28.52	29.83	28.17	28.10	28.37	28.45	27.80	28.07	28.73
232	hsa-miR-376b-4373196	35.00	35.00	35.00	35.00	35.00	35.00	34.39	35.00	35.00	35.00
233	hsa-miR-380-5p-4373021	30.37	31.71	32.11	31.55	30.08	31.35	32.28	30.98	30.95	31.53
234	hsa-miR-410-4378093	26.42	26.86	28.41	26.58	26.39	26.81	26.49	26.31	26.78	26.91
235	hsa-miR-412-4373199	35.00	35.00	35.00	35.00	35.00	34.91	35.00	34.25	35.00	35.00
236	hsa-miR-432-4378076	33.51	35.00	35.00	33.85	35.00	35.00	34.34	35.00	35.00	35.00
237	hsa-miR-512-5p-4373238	35.00	35.00	35.00	35.00	35.00	35.00	35.00	35.00	35.00	35.00
238	hsa-miR-9-4378074	23.05	23.08	24.23	22.54	22.37	22.57	22.86	22.82	23.61	24.60
239	hsa-miR-99a-4373008	22.94	23.23	23.53	22.96	22.61	23.48	23.46	23.65	24.03	22.42
240	hsa-miR-99b-4373007	25.85	26.24	27.20	25.94	26.22	26.31	27.02	26.77	27.36	26.08
241	hsa-miR-15a-4373123	27.50	28.29	28.30	28.46	26.83	28.75	27.01	26.85	27.75	27.54
242	hsa-miR-15b-4373122	26.23	26.49	27.07	26.63	25.73	26.90	28.44	27.11	28.49	28.29
243	hsa-miR-199a-4373272	31.70	34.68	34.56	33.74	34.11	34.72	35.00	33.97	35.00	35.00
244	hsa-miR-199a-4378068	29.70	30.50	31.92	30.43	30.16	31.00	31.36	31.75	31.62	31.26
245	hsa-miR-199b-4373100	33.03	33.25	35.00	35.00	32.93	34.40	33.74	33.93	33.48	35.00
246	hsa-miR-219-4373080	26.58	26.17	25.38	27.32	23.83	26.15	24.58	25.54	25.95	28.08
247	hsa-miR-25-4373071	26.80	27.13	27.39	27.73	26.29	27.95	28.27	28.11	28.47	27.11
248	hsa-miR-30e-5p-4373058	26.07	26.23	26.79	26.37	25.35	26.11	26.01	25.31	26.16	27.28
249	hsa-miR-323-4373054	27.02	27.33	29.32	27.19	27.31	27.58	29.46	28.23	29.16	29.69
250	hsa-miR-338-4373043	27.55	27.68	27.10	28.40	26.56	28.34	26.47	26.68	27.68	26.33
253	hsa-miR-368-4373033	34.68	35.00	35.00	35.00	35.00	35.00	35.00	35.00	35.00	35.00
254	hsa-miR-373-4378073	35.00	35.00	35.00	35.00	35.00	35.00	35.00	35.00	35.00	35.00
255	hsa-miR-373-4373279	35.00	35.00	35.00	35.00	35.00	35.00	35.00	35.00	35.00	35.00
256	hsa-miR-382-4373019	26.02	26.04	28.02	26.31	26.28	26.36	26.68	26.21	26.99	26.89
257	hsa-miR-424-4373201	30.94	32.16	33.03	32.26	31.71	32.56	31.07	31.40	32.38	35.00
258	hsa-miR-448-4373206	34.72	35.00	35.00	35.00	35.00	35.00	35.00	34.98	35.00	35.00
259	hsa-miR-450-4373208	35.00	35.00	35.00	35.00	35.00	35.00	34.84	35.00	35.00	35.00
260	hsa-miR-451-4373209	28.06	28.21	28.81	27.80	27.56	27.78	27.55	27.65	28.57	28.34
261	hsa-miR-452-4373281	34.47	35.00	35.00	35.00	35.00	35.00	35.00	35.00	35.00	34.71
262	hsa-miR-452-4378077	33.03	33.92	34.73	33.05	32.77	32.74	32.34	32.46	33.44	35.00
263	hsa-miR-453-4373210	33.45	34.36	35.00	35.00	35.00	34.51	35.00	35.00	34.59	35.00
264	hsa-miR-485-3p-4378095	25.61	26.00	27.78	25.77	26.16	26.09	27.32	27.01	27.63	28.09
265	hsa-miR-486-4378096	28.10	28.14	28.59	27.83	27.60	28.06	28.67	27.88	28.66	28.14
266	hsa-miR-488-4373213	30.09	30.81	32.80	30.62	30.70	30.94	30.70	30.63	31.90	32.81
267	hsa-miR-490-4373215	29.73	30.74	32.15	30.16	29.89	31.53	30.41	29.79	31.06	30.34
268	hsa-miR-492-4373217	35.00	35.00	35.00	35.00	35.00	35.00	35.00	35.00	35.00	35.00
269	hsa-miR-493-4373218	35.00	35.00	35.00	35.00	35.00	35.00	35.00	35.00	35.00	35.00
270	hsa-miR-503-4373228	33.09	35.00	35.00	35.00	34.06	35.00	34.10	33.65	35.00	35.00
271	hsa-miR-504-4373229	30.86	30.31	32.65	30.44	30.87	30.59	32.29	30.84	32.42	31.56
272	hsa-miR-505-4373230	34.73	35.00	35.00	35.00	35.00	35.00	35.00	35.00	35.00	35.00
273	hsa-miR-507-4373232	35.00	35.00	35.00	35.00	35.00	35.00	35.00	35.00	35.00	35.00
274	hsa-miR-513-4373239	35.00	35.00	35.00	35.00	35.00	35.00	35.00	35.00	35.00	35.00
275	hsa-miR-516-5p-4378099	35.00	35.00	35.00	35.00	35.00	35.00	35.00	35.00	35.00	35.00
276	hsa-miR-517-4378078	35.00	35.00	35.00	35.00	35.00	35.00	35.00	35.00	35.00	35.00
277	4343438-Blank	35.00	35.00	35.00	35.00	35.00	35.00	35.00	35.00	35.00	35.00
278	hsa-miR-518c-4378082	35.00	35.00	35.00	35.00	35.00	35.00	35.00	35.00	35.00	35.00
279	hsa-miR-518f-4378083	35.00	35.00	35.00	35.00	35.00	35.00	35.00	35.00	35.00	35.00
280	hsa-miR-519b-4373250	35.00	35.00	35.00	35.00	35.00	35.00	35.00	35.00	35.00	35.00
281	hsa-miR-519c-4373251	35.00	35.00	35.00	35.00	35.00	35.00	35.00	35.00	35.00	35.00

Supplementary Table 1

282	hsa-miR-519d-4373266	35.00	35.00	35.00	35.00	35.00	35.00	35.00	35.00	35.00	35.00
283	hsa-miR-519e-4373267	35.00	35.00	35.00	35.00	35.00	35.00	35.00	35.00	35.00	35.00
284	hsa-miR-522-4373245	35.00	35.00	35.00	34.84	35.00	35.00	35.00	35.00	35.00	35.00
285	hsa-miR-523-4373260	35.00	35.00	35.00	35.00	35.00	35.00	35.00	35.00	35.00	35.00
286	hsa-miR-524-4378087	34.82	34.27	35.00	34.47	34.81	35.00	35.00	34.24	35.00	33.44
287	hsa-miR-526b-4378080	35.00	35.00	35.00	35.00	35.00	35.00	35.00	35.00	35.00	35.00
288	hsa-miR-96-4373010	35.00	35.00	35.00	35.00	35.00	35.00	30.71	35.00	35.00	35.00
289	hsa-miR-651-4381007	35.00	35.00	35.00	35.00	35.00	35.00	35.00	35.00	35.00	35.00
290	hsa-miR-376a-4378104	30.08	30.39	32.10	30.21	29.80	30.06	30.05	29.86	30.89	30.05
291	hsa-miR-542-5p-4378105	33.87	34.56	35.00	33.83	33.13	34.83	33.40	33.59	35.00	35.00
292	hsa-miR-545-4380918	34.69	32.74	32.54	35.00	30.90	31.70	32.01	32.07	32.26	35.00
293	hsa-miR-544-4380919	35.00	35.00	35.00	35.00	35.00	35.00	35.00	35.00	35.00	35.00
294	hsa-miR-656-4380920	28.11	28.64	30.42	27.98	28.61	28.90	29.42	28.05	28.71	28.82
295	hsa-miR-549-4380921	35.00	35.00	35.00	35.00	35.00	35.00	35.00	35.00	35.00	34.81
296	hsa-miR-657-4380922	35.00	35.00	35.00	35.00	35.00	35.00	35.00	35.00	35.00	35.00
297	hsa-miR-658-4380923	35.00	35.00	35.00	35.00	35.00	35.00	35.00	35.00	35.00	35.00
298	hsa-miR-660-4380925	27.62	27.67	28.60	27.58	27.18	28.06	27.92	27.45	28.31	28.76
301	hsa-miR-425-5p-4380926	26.41	26.92	28.04	26.63	26.54	27.37	27.66	27.19	28.15	27.60
302	hsa-miR-652-4380927	35.00	35.00	35.00	35.00	35.00	35.00	35.00	35.00	35.00	35.00
303	hsa-miR-532-4380928	27.68	28.56	28.98	28.06	27.91	28.51	28.50	27.94	28.70	28.54
304	hsa-miR-551a-4380929	35.00	35.00	35.00	35.00	35.00	35.00	35.00	35.00	35.00	35.00
305	hsa-miR-552-4380930	35.00	35.00	35.00	35.00	35.00	35.00	35.00	35.00	35.00	35.00
306	hsa-miR-553-4380931	35.00	35.00	35.00	35.00	35.00	35.00	35.00	35.00	35.00	35.00
307	hsa-miR-554-4380932	35.00	35.00	35.00	35.00	35.00	35.00	35.00	35.00	35.00	35.00
308	hsa-miR-555-4380933	35.00	35.00	35.00	35.00	35.00	35.00	35.00	35.00	35.00	35.00
309	hsa-miR-562-4380939	35.00	35.00	35.00	35.00	35.00	35.00	35.00	35.00	35.00	35.00
310	hsa-miR-563-4380940	35.00	35.00	35.00	35.00	34.44	35.00	35.00	35.00	35.00	35.00
311	hsa-miR-564-4380941	35.00	34.11	35.00	34.66	33.14	35.00	33.14	31.66	35.00	33.16
312	hsa-miR-565-4380942	26.87	28.78	28.15	26.22	28.10	27.30	29.42	28.40	27.17	27.15
313	hsa-miR-566-4380943	35.00	33.93	35.00	35.00	32.37	32.36	34.61	32.95	35.00	35.00
314	hsa-miR-551b-4380945	32.36	31.24	34.01	31.81	31.07	33.06	31.24	31.35	32.21	35.00
315	hsa-miR-569-4380946	35.00	35.00	35.00	35.00	35.00	35.00	35.00	35.00	35.00	35.00
316	hsa-miR-570-4380947	35.00	35.00	35.00	35.00	35.00	35.00	35.00	35.00	35.00	35.00
317	hsa-miR-548a-4380948	35.00	35.00	35.00	35.00	35.00	35.00	35.00	35.00	35.00	35.00
318	hsa-miR-586-4380949	35.00	35.00	35.00	35.00	35.00	35.00	35.00	35.00	35.00	35.00
319	hsa-miR-587-4380950	35.00	35.00	35.00	35.00	35.00	35.00	35.00	35.00	35.00	35.00
320	hsa-miR-548b-4380951	35.00	35.00	35.00	35.00	35.00	35.00	35.00	35.00	35.00	35.00
321	hsa-miR-588-4380952	35.00	35.00	35.00	35.00	35.00	35.00	35.00	35.00	35.00	35.00
322	hsa-miR-589-4380953	33.73	35.00	35.00	35.00	33.75	35.00	35.00	35.00	34.63	35.00
323	hsa-miR-550-4380954	29.76	30.74	31.58	30.46	30.17	31.98	30.95	30.49	31.28	30.30
324	hsa-miR-591-4380955	35.00	35.00	35.00	35.00	35.00	35.00	35.00	35.00	35.00	35.00
325	hsa-miR-593-4380957	35.00	35.00	35.00	35.00	35.00	35.00	35.00	35.00	35.00	35.00
326	hsa-miR-594-4380958	24.12	26.99	27.45	23.71	26.16	25.90	28.99	29.93	26.00	24.76
327	hsa-miR-596-4380959	35.00	35.00	35.00	35.00	34.93	35.00	34.95	35.00	35.00	35.00
328	hsa-miR-597-4380960	31.02	31.00	32.06	30.83	30.55	32.14	31.01	30.69	30.84	33.14
329	hsa-miR-622-4380961	35.00	35.00	35.00	35.00	35.00	35.00	35.00	35.00	35.00	35.00
330	hsa-miR-599-4380962	35.00	35.00	35.00	35.00	35.00	35.00	35.00	35.00	35.00	35.00
331	hsa-miR-600-4380963	35.00	35.00	35.00	35.00	35.00	35.00	35.00	35.00	35.00	35.00
332	hsa-miR-624-4380964	35.00	35.00	35.00	35.00	35.00	35.00	35.00	34.72	35.00	35.00
333	hsa-miR-601-4380965	35.00	35.00	35.00	34.65	33.09	35.00	34.50	32.97	35.00	33.99
334	hsa-miR-626-4380966	35.00	35.00	35.00	35.00	35.00	35.00	34.66	35.00	35.00	35.00
335	hsa-miR-629-4380969	34.10	35.00	34.39	33.53	34.53	35.00	35.00	34.74	34.75	33.90
336	hsa-miR-548d-4381008	35.00	35.00	35.00	35.00	35.00	35.00	35.00	35.00	35.00	35.00
337	hsa-miR-639-4380987	33.81	33.63	34.86	33.64	31.34	32.81	31.62	34.44	31.67	33.12
338	hsa-miR-613-4380989	35.00	35.00	35.00	35.00	35.00	35.00	35.00	35.00	35.00	35.00

Supplementary Table 1

339	hsa-miR-614-4380990	35.00	35.00	35.00	35.00	35.00	35.00	35.00	35.00	35.00	35.00
340	hsa-miR-615-4380991	35.00	35.00	35.00	35.00	35.00	35.00	35.00	35.00	35.00	35.00
341	hsa-miR-616-4380992	35.00	35.00	35.00	35.00	35.00	35.00	35.00	35.00	35.00	33.26
342	hsa-miR-548c-4380993	35.00	35.00	35.00	35.00	35.00	35.00	35.00	35.00	35.00	35.00
343	hsa-miR-617-4380994	35.00	35.00	35.00	35.00	35.00	35.00	35.00	34.88	35.00	33.98
344	hsa-miR-642-4380995	31.71	31.88	29.69	31.62	29.35	31.78	31.48	32.44	32.29	29.31
345	hsa-miR-618-4380996	34.64	34.48	34.97	35.00	33.35	34.82	32.85	34.24	35.00	35.00
346	hsa-miR-644-4380999	35.00	35.00	35.00	35.00	35.00	35.00	35.00	34.13	35.00	35.00
349	hsa-miR-646-4381002	32.91	34.68	34.32	34.85	32.99	32.89	32.76	32.49	33.70	32.69
350	hsa-miR-647-4381003	35.00	35.00	35.00	35.00	35.00	35.00	35.00	35.00	35.00	35.00
351	hsa-miR-649-4381005	35.00	35.00	35.00	35.00	34.73	35.00	35.00	35.00	35.00	34.22
352	hsa-miR-650-4381006	30.24	30.16	30.66	30.34	29.18	30.21	30.08	29.48	30.45	29.76
353	hsa-miR-661-4381009	35.00	35.00	35.00	35.00	34.89	35.00	35.00	35.00	35.00	35.00
354	hsa-miR-662-4381010	35.00	35.00	35.00	35.00	35.00	35.00	35.00	35.00	35.00	35.00
355	hsa-miR-449b-4381011	33.04	34.93	35.00	32.71	34.32	34.17	33.35	35.00	34.71	35.00
356	hsa-miR-653-4381012	35.00	35.00	35.00	35.00	33.73	34.73	35.00	35.00	35.00	35.00
357	hsa-miR-411-4381013	26.52	26.81	28.31	26.52	26.35	26.82	26.60	26.07	26.42	27.12
358	hsa-miR-654-4381014	33.30	34.42	35.00	35.00	35.00	35.00	34.12	34.99	35.00	35.00
359	hsa-miR-572-4381017	32.20	32.06	32.93	32.04	31.18	32.33	32.03	30.62	31.79	31.15
360	4343438-Blank	35.00	35.00	35.00	35.00	35.00	35.00	35.00	35.00	35.00	35.00
361	hsa-miR-575-4381020	35.00	35.00	35.00	35.00	32.32	35.00	35.00	33.57	35.00	35.00
362	hsa-miR-576-4381021	33.44	33.85	34.90	33.27	34.75	34.16	35.00	35.00	35.00	35.00
363	hsa-miR-578-4381022	35.00	35.00	35.00	35.00	35.00	35.00	35.00	35.00	35.00	35.00
364	hsa-miR-579-4381023	35.00	35.00	35.00	33.59	32.85	35.00	33.67	35.00	35.00	35.00
365	hsa-miR-580-4381024	35.00	35.00	35.00	35.00	35.00	35.00	35.00	35.00	35.00	35.00
366	hsa-miR-585-4381027	35.00	35.00	35.00	35.00	35.00	35.00	35.00	35.00	35.00	35.00
367	hsa-miR-200b-4381028	35.00	34.30	33.81	35.00	32.85	34.78	35.00	35.00	35.00	34.89
368	hsa-miR-484-4381032	24.65	25.26	25.78	25.00	24.50	25.16	25.10	24.88	25.50	25.51
369	hsa-miR-512-3p-4381034	35.00	35.00	35.00	35.00	34.08	35.00	35.00	35.00	35.00	35.00
370	hsa-miR-631-4380971	35.00	35.00	35.00	35.00	35.00	35.00	35.00	35.00	35.00	35.00
371	hsa-miR-363-4380917	35.00	35.00	35.00	34.97	35.00	35.00	35.00	35.00	35.00	35.00
372	hsa-miR-487b-4378102	24.75	25.20	26.75	24.72	24.69	25.21	25.66	25.17	25.75	25.94
373	hsa-miR-645-4381000	35.00	35.00	35.00	35.00	35.00	35.00	35.00	35.00	35.00	35.00
374	hsa-miR-659-4380924	32.68	33.51	35.00	33.68	34.16	33.92	34.11	35.00	35.00	35.00
375	hsa-miR-556-4380934	34.93	35.00	35.00	35.00	35.00	35.00	34.74	35.00	35.00	35.00
376	hsa-miR-558-4380936	35.00	35.00	35.00	35.00	35.00	35.00	35.00	35.00	35.00	35.00
377	hsa-miR-627-4380967	35.00	35.00	34.55	35.00	34.14	35.00	35.00	33.92	35.00	32.20
378	hsa-miR-630-4380970	35.00	33.90	35.00	35.00	33.67	35.00	35.00	34.04	34.47	34.07
379	hsa-miR-603-4380972	35.00	35.00	35.00	35.00	35.00	35.00	35.00	35.00	35.00	35.00
380	hsa-miR-606-4380974	35.00	35.00	35.00	35.00	35.00	35.00	35.00	35.00	35.00	35.00
381	hsa-miR-607-4380975	35.00	35.00	35.00	35.00	35.00	35.00	35.00	35.00	35.00	35.00
382	hsa-miR-608-4380976	35.00	35.00	35.00	35.00	35.00	35.00	35.00	35.00	35.00	35.00
383	hsa-miR-609-4380978	35.00	35.00	35.00	35.00	35.00	35.00	35.00	35.00	35.00	35.00
384	hsa-miR-633-4380979	35.00	35.00	35.00	35.00	35.00	35.00	35.00	35.00	35.00	35.00

Supplementary Table 1

								Depression Means	Normal Control Group			
11	12	13	14	15	16	17	18		19	20	21	22
24.70	25.10	25.08	24.05	25.23	25.11	25.17	25.23	25.00	24.74	25.34	24.56	24.62
22.46	23.04	23.57	23.28	23.51	23.32	24.08	23.36	23.39	23.10	22.82	23.44	23.67
23.37	23.71	23.66	23.95	24.02	23.68	24.33	24.09	23.90	23.46	24.22	23.62	24.01
27.24	27.60	27.12	26.97	27.48	26.95	27.22	27.11	27.05	27.08	27.06	26.57	26.64
29.33	29.07	28.60	27.88	28.85	28.80	28.26	29.27	28.55	27.75	28.20	27.25	27.82
23.10	23.53	23.65	23.20	23.69	23.49	23.59	23.73	23.60	23.62	23.69	23.01	23.83
32.36	32.03	32.75	35.00	34.60	32.55	33.32	34.44	32.84	32.14	27.80	31.49	29.82
35.00	35.00	35.00	35.00	35.00	35.00	35.00	35.00	35.00	35.00	35.00	35.00	35.00
32.82	31.30	35.00	30.61	31.35	31.19	30.81	31.40	31.71	31.44	30.62	30.56	31.26
30.50	29.64	29.92	29.33	29.71	29.65	29.50	29.31	29.73	29.82	29.89	29.00	29.86
23.81	24.11	25.17	25.03	24.65	24.57	24.66	25.23	24.88	25.45	25.59	24.39	24.86
30.79	30.56	33.00	30.44	31.67	30.15	30.68	30.03	30.90	30.89	29.74	30.83	30.73
25.25	26.35	26.35	26.58	26.19	25.11	25.27	25.76	26.23	26.86	26.05	26.35	26.41
23.22	23.27	23.80	23.90	24.06	23.05	23.26	23.34	23.69	23.80	23.36	23.28	23.49
25.93	26.59	27.28	26.43	27.36	25.93	26.36	26.30	26.56	26.63	25.60	25.75	25.50
22.46	22.56	23.20	23.14	22.74	22.27	22.60	22.51	22.74	22.90	22.60	22.22	22.92
30.87	32.48	29.58	29.37	30.27	28.94	28.96	29.33	29.83	29.57	29.58	28.50	29.92
35.00	32.97	33.27	34.53	33.39	30.80	32.15	31.64	32.94	32.55	32.02	33.15	33.43
35.00	35.00	33.90	32.85	33.41	31.43	31.25	31.69	32.86	32.08	30.98	33.15	35.00
23.88	23.93	25.05	24.15	24.19	23.87	24.12	24.12	24.09	24.00	23.84	23.81	23.85
25.11	24.64	24.96	24.82	25.08	24.72	25.09	24.99	25.01	25.08	24.20	24.65	24.43
29.46	28.97	28.03	28.56	28.46	27.72	28.07	28.10	28.38	28.17	27.96	27.39	28.19
35.00	35.00	35.00	33.53	35.00	35.00	35.00	35.00	34.80	35.00	35.00	34.54	35.00
27.32	27.65	25.96	25.29	27.17	25.13	25.21	25.81	26.24	26.24	24.37	25.98	23.69
28.08	28.61	27.46	29.74	30.08	29.25	29.20	29.31	29.15	26.57	28.86	26.60	26.49
26.63	26.87	26.88	26.64	27.02	26.50	26.88	26.56	26.85	26.98	26.83	26.17	26.10
28.88	28.68	30.18	29.69	29.62	29.65	30.23	29.74	30.05	29.69	29.18	28.96	28.40
28.96	29.37	29.71	30.39	29.04	28.66	28.73	29.27	29.22	29.55	29.34	28.68	29.05
22.55	22.66	23.07	23.42	23.10	22.96	23.39	23.36	23.37	23.70	24.09	23.10	23.77
30.47	30.34	30.44	30.81	30.96	31.70	30.74	30.84	30.85	30.93	29.84	30.57	30.51
35.00	35.00	34.14	35.00	35.00	33.86	34.95	35.00	34.81	34.10	33.79	33.09	33.69
24.58	24.79	23.72	23.24	23.75	22.92	23.49	23.18	23.76	23.26	23.58	22.63	23.13
25.26	25.26	25.52	24.90	24.70	24.16	24.54	24.59	24.69	24.12	24.85	23.38	24.04
23.33	23.25	23.81	23.76	24.50	23.69	24.11	24.19	24.11	24.55	23.41	24.12	23.22
27.81	28.37	26.86	26.70	27.70	25.89	25.96	26.11	26.87	26.54	26.87	25.75	26.53
29.26	28.81	29.34	28.06	28.73	28.16	28.34	28.41	28.41	28.03	27.47	27.37	27.23
35.00	35.00	33.38	35.00	35.00	35.00	35.00	35.00	34.67	35.00	33.22	35.00	35.00
31.93	30.79	30.33	29.71	31.84	31.46	29.96	31.18	30.85	30.39	30.42	30.07	30.62
33.44	32.21	31.28	30.91	31.10	30.45	32.50	30.82	31.42	30.86	29.78	29.44	30.46
25.39	25.28	25.35	24.31	25.35	24.41	24.61	24.58	25.05	24.89	24.19	24.57	24.42
20.90	21.03	20.22	20.16	20.50	19.44	19.59	19.99	20.17	19.71	19.81	19.10	19.71
23.10	23.37	22.30	22.28	22.77	21.71	22.15	22.01	22.50	21.86	22.20	21.65	21.68
24.91	24.96	24.65	24.85	24.72	24.63	24.59	24.91	24.93	24.82	25.07	24.37	24.86
22.35	22.58	23.44	23.19	22.99	22.62	23.33	22.88	23.11	23.49	22.86	22.93	24.38
24.59	25.07	24.59	24.76	24.64	24.08	24.65	24.65	24.68	24.35	24.88	23.97	24.05
35.00	35.00	35.00	35.00	35.00	35.00	35.00	35.00	34.63	35.00	32.12	34.87	35.00
29.54	29.13	28.68	29.28	29.73	28.58	28.93	29.41	29.15	29.00	28.21	28.43	28.74
28.45	30.25	28.83	27.97	29.44	27.78	27.23	28.56	28.43	29.36	28.42	27.97	28.17
19.41	19.51	19.82	20.02	19.75	19.42	19.41	19.73	19.73	19.18	20.26	19.28	19.92
23.46	23.72	24.01	23.85	24.26	23.05	23.19	23.54	23.56	23.31	23.46	23.18	23.50

Supplementary Table 1

26.52	26.89	25.32	25.80	26.25	25.08	25.28	25.54	25.84	25.52	25.35	25.25	25.72
24.96	25.15	24.75	25.20	25.32	24.19	24.76	24.53	25.01	24.96	24.84	24.59	25.60
24.47	24.90	24.20	23.94	24.33	23.52	23.74	23.90	24.03	23.57	24.22	23.01	23.83
22.75	22.71	22.35	22.47	22.36	22.21	22.20	22.68	22.58	22.39	23.12	21.95	22.92
20.48	20.40	20.33	20.84	20.68	20.19	20.41	20.45	20.61	20.50	20.74	20.01	20.85
26.09	26.95	27.27	27.33	27.06	26.94	26.84	27.36	27.25	27.75	27.24	26.54	26.72
35.00	35.00	35.00	35.00	35.00	35.00	35.00	35.00	34.94	35.00	35.00	35.00	35.00
28.93	28.85	28.76	28.30	28.98	28.12	28.28	28.33	28.64	28.37	28.98	27.74	28.37
23.04	23.26	23.12	23.45	23.20	22.57	22.97	22.86	22.87	21.83	22.65	21.49	21.87
30.47	30.55	32.55	31.07	31.31	30.90	31.12	31.13	31.05	31.04	29.62	30.44	29.79
35.00	35.00	35.00	35.00	35.00	35.00	35.00	35.00	35.00	35.00	35.00	35.00	35.00
35.00	35.00	35.00	35.00	35.00	35.00	35.00	35.00	35.00	35.00	35.00	35.00	35.00
34.38	34.70	35.00	35.00	35.00	35.00	35.00	35.00	34.66	35.00	34.09	34.80	35.00
27.39	27.08	26.62	26.94	27.21	25.56	26.29	25.97	26.60	25.85	25.85	25.18	25.52
35.00	35.00	35.00	32.78	35.00	32.16	35.00	35.00	34.41	35.00	35.00	35.00	35.00
27.70	27.57	28.70	28.75	28.23	27.71	28.11	28.35	28.06	27.57	27.83	27.34	27.56
26.19	26.36	26.88	26.45	26.76	26.19	26.35	26.68	26.68	26.59	26.91	26.26	26.83
30.95	31.43	30.98	31.34	31.30	32.14	31.60	31.72	31.41	31.76	31.25	30.47	30.91
35.00	34.05	32.21	31.64	35.00	32.69	31.93	32.16	33.01	32.97	33.98	32.40	32.58
27.70	27.99	26.97	27.91	27.88	26.71	27.18	27.55	27.51	27.11	28.11	26.26	26.42
30.77	29.61	26.70	31.43	31.71	30.45	29.96	31.10	29.73	28.00	27.91	27.98	28.69
35.00	35.00	35.00	34.30	33.48	35.00	31.88	32.36	33.39	33.27	35.00	35.00	30.33
35.00	35.00	35.00	35.00	35.00	35.00	35.00	35.00	34.92	35.00	35.00	32.35	35.00
33.52	35.00	35.00	32.31	35.00	32.82	33.90	35.00	33.36	31.48	31.53	34.45	32.35
35.00	35.00	35.00	35.00	35.00	35.00	35.00	35.00	34.80	35.00	33.69	35.00	32.69
34.45	34.54	35.00	35.00	34.01	35.00	35.00	33.91	34.59	35.00	32.56	35.00	34.63
35.00	35.00	35.00	35.00	35.00	35.00	35.00	35.00	35.00	35.00	34.73	35.00	35.00
35.00	35.00	32.21	33.94	35.00	35.00	35.00	35.00	34.33	35.00	31.31	33.41	32.04
35.00	35.00	35.00	35.00	35.00	35.00	35.00	35.00	35.00	35.00	34.18	35.00	34.16
34.33	34.89	32.27	31.26	32.98	31.58	30.91	31.05	32.20	31.41	31.18	30.45	30.40
35.00	35.00	35.00	35.00	35.00	35.00	35.00	35.00	35.00	35.00	34.20	35.00	32.94
31.93	33.35	31.43	30.52	34.42	33.51	35.00	33.87	33.53	35.00	30.57	33.58	33.52
35.00	35.00	35.00	32.46	35.00	35.00	35.00	35.00	34.73	35.00	33.57	35.00	32.42
31.58	31.66	32.82	35.00	35.00	35.00	34.20	35.00	33.97	33.82	30.46	34.14	35.00
35.00	35.00	35.00	35.00	35.00	35.00	35.00	35.00	34.96	34.23	32.26	35.00	33.86
35.00	35.00	35.00	34.94	35.00	35.00	35.00	35.00	35.00	35.00	35.00	35.00	35.00
35.00	35.00	35.00	35.00	35.00	35.00	35.00	35.00	34.98	35.00	35.00	35.00	35.00
35.00	35.00	35.00	35.00	35.00	35.00	35.00	35.00	34.97	35.00	35.00	35.00	35.00
35.00	35.00	35.00	33.61	35.00	35.00	35.00	35.00	34.92	35.00	33.42	35.00	33.98
35.00	35.00	35.00	35.00	35.00	35.00	35.00	35.00	35.00	35.00	35.00	35.00	35.00
34.95	35.00	35.00	35.00	35.00	35.00	35.00	35.00	34.95	35.00	35.00	34.63	34.55
34.27	35.00	35.00	35.00	35.00	33.43	35.00	33.85	34.61	35.00	34.93	32.92	33.29
35.00	35.00	35.00	35.00	35.00	35.00	35.00	35.00	34.92	35.00	35.00	35.00	35.00
25.79	26.15	25.83	26.26	25.95	25.52	25.88	25.96	25.97	25.95	26.08	25.11	25.78
23.39	23.97	22.75	22.81	23.43	22.15	22.45	22.62	22.98	23.07	23.56	22.25	23.02
23.85	23.75	23.26	22.99	23.38	22.14	22.13	22.68	22.93	22.42	23.37	21.89	22.51
24.97	24.89	24.60	24.74	25.09	23.97	24.38	24.76	24.71	24.61	25.34	23.98	24.79
25.64	25.93	25.82	25.92	26.23	25.33	25.59	25.89	25.89	25.92	26.14	25.04	25.80
25.45	25.97	25.37	26.08	25.72	25.54	25.29	25.90	25.80	25.98	26.45	25.43	25.95
21.97	22.30	23.29	23.04	23.44	22.74	22.92	23.19	22.99	23.04	22.96	22.39	23.23
27.54	27.99	26.86	26.51	27.38	26.15	26.30	26.39	26.94	26.69	26.78	26.04	26.75
23.93	24.99	24.91	24.90	24.91	24.24	24.08	25.03	24.87	25.11	25.55	24.76	24.79
23.31	23.14	23.53	23.60	23.32	23.38	23.44	23.47	23.50	23.07	23.78	22.52	23.44
21.26	21.63	21.43	22.62	21.88	21.33	21.66	22.06	21.81	21.64	22.06	21.24	21.97
29.95	30.49	29.81	28.57	30.34	29.04	29.05	29.52	29.49	29.92	29.95	28.63	29.19

Supplementary Table 1

24.94	25.84	25.46	25.02	25.94	24.69	24.47	25.05	25.29	24.82	25.76	24.38	24.69
27.79	29.58	28.41	28.42	28.53	28.21	27.25	28.43	28.72	28.90	29.29	28.40	28.99
30.53	29.96	30.19	29.72	30.10	28.85	28.63	29.03	29.45	28.93	28.65	28.44	27.50
29.81	29.19	27.98	27.80	28.85	27.29	27.40	27.45	28.14	27.52	27.63	26.74	27.22
30.36	30.02	30.51	29.27	30.77	30.83	30.10	31.13	30.72	30.92	31.43	29.91	30.39
27.40	27.70	27.74	27.79	27.89	27.24	27.66	27.53	27.84	27.80	28.34	27.30	27.57
28.84	28.63	29.64	29.27	29.32	28.86	29.24	28.84	29.13	29.32	29.27	28.46	28.61
27.40	28.57	27.84	27.81	28.15	26.90	27.23	27.99	27.77	27.35	28.51	26.43	27.66
25.00	24.98	25.28	24.87	25.11	24.89	25.30	25.15	25.04	24.96	24.82	24.07	24.75
31.85	34.72	32.46	32.82	35.00	33.97	35.00	35.00	34.00	35.00	33.60	33.48	35.00
35.00	28.62	29.62	29.94	29.72	29.72	31.08	30.43	31.27	29.00	32.67	29.43	28.55
30.22	34.25	35.00	35.00	35.00	35.00	35.00	35.00	33.83	35.00	35.00	32.93	32.63
35.00	35.00	35.00	33.28	35.00	35.00	35.00	35.00	34.87	35.00	35.00	31.98	30.83
28.69	28.15	29.42	28.93	29.27	28.54	28.56	28.89	28.92	29.04	28.54	28.36	29.50
26.17	26.77	26.52	26.22	27.34	25.32	25.65	25.87	26.23	25.82	26.52	25.06	25.63
35.00	35.00	35.00	35.00	35.00	35.00	35.00	35.00	35.00	35.00	35.00	35.00	35.00
26.77	27.04	26.51	26.27	26.78	25.96	26.28	26.48	26.60	26.74	26.77	25.75	26.63
31.19	32.33	31.87	31.51	32.48	31.35	32.12	31.47	32.11	32.07	31.09	32.11	32.48
26.66	26.90	26.60	26.90	26.36	26.06	26.55	26.73	26.67	26.60	26.74	25.95	26.60
32.88	35.00	33.73	35.00	35.00	35.00	35.00	35.00	34.78	35.00	35.00	35.00	35.00
23.91	23.93	23.12	23.17	23.41	22.55	22.50	23.00	23.19	22.75	23.32	22.29	22.75
29.63	29.69	29.52	29.37	29.42	29.73	29.01	29.58	29.44	29.34	28.91	28.92	29.29
35.00	35.00	35.00	35.00	35.00	34.12	33.43	34.87	34.12	33.71	33.29	33.45	31.27
30.03	29.85	29.77	28.39	29.57	28.31	28.51	28.85	29.02	28.68	29.76	27.55	28.22
26.87	26.73	26.56	26.03	26.69	26.12	26.15	26.17	26.43	26.15	26.73	25.51	27.03
31.04	31.38	30.32	30.59	32.32	30.44	31.05	31.40	31.21	31.01	31.77	29.64	30.98
25.94	26.04	26.24	25.96	26.22	26.29	26.77	26.52	26.42	26.12	26.34	25.40	26.60
24.67	24.79	24.76	24.97	24.75	24.61	24.96	24.77	24.88	24.52	25.08	23.98	24.80
29.69	29.25	29.60	28.66	29.41	29.02	28.91	28.96	29.05	28.29	28.93	27.61	28.40
32.24	32.32	30.64	31.57	33.65	31.43	33.58	31.98	32.60	31.51	31.91	31.91	31.72
34.27	35.00	35.00	34.44	33.96	35.00	33.06	32.22	33.80	31.37	32.51	31.74	32.88
35.00	35.00	35.00	35.00	35.00	35.00	34.68	35.00	34.98	35.00	35.00	35.00	35.00
35.00	35.00	35.00	35.00	35.00	35.00	35.00	34.53	34.14	35.00	34.66	34.04	35.00
35.00	35.00	35.00	35.00	35.00	35.00	35.00	35.00	35.00	35.00	35.00	35.00	35.00
24.41	25.42	24.18	24.87	24.35	23.69	24.23	24.12	24.28	23.50	24.18	23.38	23.68
25.69	25.65	26.80	26.46	26.47	25.97	26.36	26.31	26.24	26.41	25.91	25.44	26.62
28.12	28.15	28.24	29.06	28.21	27.98	28.01	28.11	28.24	28.32	28.37	27.35	27.89
27.83	28.81	28.48	28.35	29.08	27.53	27.72	28.07	28.41	28.41	27.79	28.52	28.91
27.93	28.19	27.64	28.05	27.94	27.89	28.34	28.14	28.22	27.93	28.68	27.57	28.28
35.00	35.00	35.00	35.00	35.00	35.00	35.00	35.00	35.00	35.00	35.00	35.00	35.00
24.31	24.20	24.58	24.66	24.54	24.07	24.18	24.47	24.31	23.68	24.85	23.16	23.81
29.52	30.83	28.90	30.03	29.56	28.50	27.88	29.08	29.12	28.47	29.33	28.34	28.70
26.17	26.12	25.98	26.20	26.26	25.70	25.93	26.10	26.08	25.82	25.95	25.49	25.85
29.27	29.74	28.34	28.40	28.29	28.52	28.44	29.08	28.84	28.03	28.85	28.02	29.10
29.69	32.15	29.13	29.59	29.69	28.05	28.19	28.76	29.24	28.75	28.81	28.47	28.76
31.57	31.11	31.34	31.68	31.88	30.40	30.52	31.30	31.48	31.16	31.64	30.80	30.96
35.00	35.00	35.00	35.00	35.00	35.00	35.00	35.00	35.00	35.00	35.00	35.00	35.00
35.00	32.79	33.27	35.00	35.00	35.00	34.28	35.00	34.67	35.00	34.13	35.00	34.70
25.48	25.61	25.80	25.61	25.71	25.65	25.67	25.91	25.69	25.38	25.95	24.95	25.22
35.00	33.77	35.00	35.00	35.00	35.00	35.00	35.00	34.91	35.00	35.00	35.00	35.00
35.00	35.00	35.00	35.00	35.00	35.00	35.00	35.00	35.00	35.00	35.00	35.00	35.00
27.37	27.15	28.25	28.75	28.36	28.01	28.41	28.25	28.27	28.29	28.19	28.52	29.02
30.42	29.76	29.17	30.56	29.58	29.35	30.02	30.37	30.03	29.70	29.84	29.32	28.60
35.00	35.00	35.00	35.00	35.00	35.00	35.00	35.00	35.00	35.00	35.00	35.00	35.00
26.04	25.83	26.50	26.70	26.15	26.76	26.94	27.19	26.47	26.36	26.63	25.92	26.27

Supplementary Table 1

35.00	35.00	35.00	35.00	35.00	35.00	35.00	35.00	35.00	35.00	35.00	35.00	35.00
30.62	31.55	31.44	31.12	30.94	31.14	30.44	30.62	30.99	30.77	31.63	30.15	30.57
25.52	25.39	25.43	25.37	25.28	25.02	25.03	25.56	24.96	24.14	24.38	23.36	23.62
24.50	24.52	24.51	24.24	24.38	23.50	24.04	24.01	24.13	23.75	23.69	23.23	23.59
35.00	35.00	35.00	35.00	35.00	34.70	35.00	35.00	34.89	35.00	34.18	35.00	35.00
30.37	30.30	31.54	31.30	30.76	30.59	31.69	31.52	30.91	31.60	31.20	30.35	30.51
26.48	26.48	27.33	27.42	27.03	26.76	26.93	27.40	27.02	26.81	27.20	26.76	27.62
23.52	23.73	23.46	24.08	23.57	23.38	23.56	23.84	23.64	23.41	24.06	22.78	23.78
26.97	26.83	26.80	26.95	26.93	26.62	26.91	26.78	26.86	26.46	26.82	26.00	26.79
25.36	25.68	25.73	25.86	25.95	24.95	25.17	25.45	25.56	25.57	25.66	25.10	25.50
35.00	35.00	35.00	35.00	35.00	35.00	35.00	35.00	35.00	35.00	35.00	35.00	35.00
30.16	35.00	29.21	32.12	31.91	32.96	35.00	33.88	32.46	32.28	31.08	32.70	33.25
27.56	27.43	28.42	27.21	27.96	28.03	27.87	28.38	27.68	26.92	27.39	26.65	27.45
24.81	24.98	26.31	26.11	25.79	25.66	26.02	25.86	25.87	26.09	25.54	25.95	26.58
32.11	32.42	31.69	31.23	33.02	31.65	31.35	32.82	32.24	31.57	32.11	32.16	29.44
33.81	34.70	34.54	35.00	34.17	35.00	34.60	35.00	34.30	33.85	34.58	33.81	32.89
32.51	32.24	32.16	33.23	32.45	31.52	31.64	33.15	32.17	31.98	32.28	30.80	32.15
27.39	27.02	27.40	26.64	26.76	26.23	25.66	26.69	26.45	26.08	26.19	25.22	25.65
27.51	27.74	28.48	27.78	28.30	27.92	27.96	28.10	28.07	27.87	28.49	27.74	28.32
33.05	32.89	32.31	34.60	35.00	31.68	32.33	33.55	33.40	35.00	32.50	33.41	35.00
26.72	26.63	26.65	27.05	26.80	26.30	26.52	26.54	26.77	26.63	27.13	25.82	26.57
33.37	35.00	35.00	34.19	33.49	33.52	33.70	34.17	33.59	33.19	34.84	32.51	32.76
35.00	35.00	35.00	33.91	35.00	32.22	33.44	35.00	33.61	31.81	31.83	31.64	31.94
32.32	35.00	32.87	33.28	35.00	33.07	33.31	32.80	33.45	32.61	34.07	34.87	34.29
29.79	29.79	29.79	29.79	29.79	29.79	29.79	29.79	29.79	29.79	29.79	29.79	29.79
26.37	27.56	27.85	27.12	27.43	27.15	26.98	27.26	27.11	26.72	26.79	26.56	27.16
35.00	33.59	32.97	33.42	34.25	34.37	33.72	33.35	34.12	35.00	34.57	32.94	33.94
26.13	26.74	26.24	25.90	26.47	25.15	25.55	25.46	25.93	25.63	25.44	25.38	25.91
25.50	26.24	25.86	26.20	26.47				26.35	26.33	26.23	25.88	26.43
23.98	24.87	21.63	21.15	22.84	21.88	22.60	22.00	22.78	22.21	22.92	22.00	22.75
22.34	22.74	22.07	22.02	22.52	22.05	21.78	22.51	22.32	22.29	22.78	21.88	21.90
30.40	30.75	30.22	29.63	30.57	29.72	29.66	30.36	30.03	29.39	30.22	28.73	29.08
29.05	30.28	28.77	29.31	29.08	28.63	28.66	29.49	28.94	28.14	28.90	28.07	28.53
27.28	27.85	25.96	25.91	26.56	25.64	26.27	25.93	26.45	25.93	26.40	25.41	26.19
28.07	28.41	27.60	27.69	28.07	27.33	27.56	28.08	27.99	28.14	28.04	27.83	28.01
28.50	28.83	28.55	28.21	28.57	27.75	27.61	28.28	28.08	27.40	27.79	26.79	26.69
24.33	24.53	24.40	23.93	24.75	23.76	23.94	24.08	24.34	24.32	24.50	23.81	24.53
29.91	30.24	28.97	29.62	29.41	29.27	28.81	28.96	29.61	29.62	30.38	29.28	28.91
28.58	28.98	28.87	28.13	28.82	29.07	28.45	29.07	28.66	28.35	28.76	27.72	27.94
21.01	21.35	21.63	21.55	21.81	21.24	21.39	21.65	21.65	21.78	21.78	21.41	21.52
35.00	35.00	35.00	35.00	35.00	35.00	35.00	35.00	35.00	35.00	35.00	35.00	35.00
33.89	35.00	33.40	35.00	35.00	35.00	35.00	33.89	34.33	33.62	33.54	35.00	35.00
35.00	35.00	34.27	32.58	33.38	32.17	31.34	33.30	32.93	32.17	31.75	31.51	35.00
35.00	35.00	35.00	35.00	35.00	35.00	35.00	35.00	35.00	35.00	35.00	35.00	35.00
23.58	24.20	23.51	23.72	23.92	23.27	23.32	23.77	23.83	23.84	24.21	23.12	23.73
31.85	31.32	31.73	31.50	31.84	31.90	32.02	30.52	31.65	31.34	30.32	31.46	32.23
35.00	35.00	32.98	35.00	35.00	35.00	35.00	35.00	34.68	33.83	35.00	35.00	35.00
35.00	35.00	35.00	35.00	35.00	35.00	35.00	35.00	35.00	35.00	35.00	35.00	35.00
25.36	25.85	25.26	25.58	26.09	25.50	26.15	25.43	26.07	26.32	26.41	26.13	27.10
31.76	31.49	31.29	30.78	30.84	30.27	30.42	30.96	30.82	30.72	29.92	28.95	29.48
35.00	35.00	32.77	33.35	35.00	34.39	35.00	33.07	34.32	32.52	34.86	33.15	35.00
22.28	22.35	21.60	21.83	21.99	21.19	21.50	21.27	21.84	21.72	22.11	21.01	21.91
23.73	24.33	23.13	23.29	23.49	22.65	22.80	22.82	23.31	23.07	23.23	22.50	23.26
35.00	35.00	35.00	35.00	35.00	35.00	34.30	34.33	34.92	35.00	35.00	35.00	35.00
35.00	35.00	35.00	35.00	35.00	34.56	34.96	35.00	34.68	33.95	35.00	34.31	33.62

Supplementary Table 1

35.00	35.00	35.00	35.00	35.00	35.00	35.00	35.00	35.00	35.00	35.00	35.00	35.00
35.00	32.73	35.00	33.34	33.41	34.62	35.00	35.00	34.63	35.00	34.29	35.00	35.00
31.91	35.00	35.00	32.06	34.15	32.89	33.73	33.50	33.54	32.07	32.20	32.40	31.54
35.00	35.00	34.45	35.00	33.11	32.57	32.95	31.13	33.76	32.32	31.50	31.80	33.00
30.31	31.09	31.13	30.84	31.50	31.62	30.94	31.52	30.96	30.64	31.34	30.34	30.02
31.89	33.93	32.90	31.61	35.00	31.96	31.98	32.10	32.99	32.41	32.59	31.64	31.67
28.04	28.39	27.64	28.17	28.35	27.85	28.08	28.07	28.28	28.40	28.75	27.98	28.40
35.00	35.00	35.00	35.00	35.00	35.00	35.00	35.00	34.97	35.00	35.00	35.00	35.00
30.20	31.79	29.71	30.49	31.93	30.98	32.65	31.70	31.24	30.94	31.59	30.52	30.72
26.79	26.94	26.35	26.42	26.48	26.05	26.40	26.46	26.66	26.52	26.86	26.18	27.12
35.00	35.00	35.00	35.00	35.00	35.00	35.00	35.00	34.95	33.85	34.97	35.00	35.00
32.86	35.00	33.50	35.00	35.00	34.60	35.00	35.00	34.59	34.16	35.00	34.52	34.66
35.00	35.00	35.00	35.00	35.00	35.00	35.00	35.00	35.00	35.00	35.00	35.00	35.00
24.59	25.00	23.43	23.52	24.38	22.89	22.61	23.10	23.40	22.44	22.74	21.99	22.66
22.30	22.49	23.31	23.05	23.60	22.93	23.09	23.20	23.13	23.14	23.30	22.59	23.18
25.74	26.00	26.12	26.49	26.58	26.29	26.52	26.89	26.42	26.61	27.06	25.71	26.62
27.54	27.95	26.96	27.03	27.63	27.14	28.03	27.12	27.59	27.54	28.35	27.44	27.97
27.86	27.22	27.53	27.44	27.56	26.55	26.58	26.92	27.17	26.82	27.01	26.27	26.04
35.00	35.00	34.56	34.19	35.00	35.00	34.83	33.15	34.40	34.32	33.94	33.47	33.02
31.33	32.25	31.34	31.15	31.64	31.52	30.79	31.89	31.20	30.29	30.28	29.22	28.58
32.20	32.69	34.43	34.23	34.08	33.81	35.00	33.61	33.88	35.00	33.56	32.41	33.04
28.40	28.11	27.02	26.63	26.86	24.87	24.98	24.69	26.18	25.55	24.46	25.55	27.83
27.24	26.81	28.08	27.14	27.48	27.27	27.25	27.67	27.46	27.68	27.51	27.08	27.05
26.47	27.52	25.90	25.53	26.26	25.36	25.05	25.89	26.09	25.90	26.01	25.58	26.24
28.85	28.87	28.87	28.94	28.46	28.33	28.27	28.82	28.43	27.56	28.15	26.72	27.58
25.83	26.14	26.91	26.78	27.02	26.19	26.54	26.28	26.91	28.13	26.75	27.67	29.00
35.00	35.00	35.00	35.00	35.00	35.00	35.00	35.00	34.98	35.00	35.00	35.00	35.00
35.00	35.00	35.00	35.00	35.00	35.00	35.00	35.00	35.00	35.00	35.00	35.00	35.00
35.00	35.00	35.00	35.00	35.00	35.00	35.00	35.00	35.00	35.00	35.00	35.00	35.00
26.20	26.62	26.32	26.38	26.75	26.60	26.64	26.82	26.56	26.10	26.38	25.98	26.59
35.00	32.37	35.00	33.43	33.06	31.44	32.63	32.55	32.67	31.26	31.59	30.63	31.20
35.00	35.00	35.00	35.00	33.98	35.00	34.58	35.00	34.90	35.00	34.96	35.00	35.00
35.00	35.00	35.00	35.00	35.00	35.00	35.00	35.00	34.99	35.00	35.00	35.00	35.00
27.95	27.41	27.66	27.11	27.80	27.29	26.75	28.10	27.80	27.79	27.28	27.80	25.06
35.00	35.00	35.00	35.00	35.00	35.00	35.00	35.00	34.95	35.00	33.64	34.54	34.62
35.00	35.00	32.23	32.68	35.00	31.88	32.40	32.22	33.33	32.26	32.35	33.30	31.57
35.00	35.00	33.45	35.00	35.00	33.90	34.09	34.01	34.58	35.00	33.58	35.00	33.82
28.30	27.61	27.57	27.27	27.36	26.42	27.20	27.03	27.01	26.09	27.36	25.21	26.20
28.03	27.37	27.88	27.85	27.62	27.62	27.32	28.64	28.00	28.07	28.24	28.18	25.70
33.27	31.87	31.31	31.11	31.35	31.33	31.52	31.03	31.38	30.92	31.41	30.45	30.61
30.39	29.62	29.69	30.43	30.78	29.84	30.55	31.11	30.46	30.52	30.12	29.70	30.94
35.00	35.00	35.00	35.00	35.00	35.00	35.00	35.00	35.00	35.00	35.00	35.00	35.00
35.00	35.00	35.00	35.00	35.00	35.00	35.00	35.00	35.00	33.84	35.00	35.00	35.00
35.00	35.00	32.91	35.00	35.00	33.42	35.00	34.52	34.49	34.87	35.00	35.00	34.57
32.22	32.39	32.27	33.86	32.65	31.75	31.14	32.31	31.75	31.25	31.73	30.90	30.14
35.00	35.00	35.00	35.00	35.00	35.00	35.00	35.00	34.98	35.00	34.94	35.00	35.00
35.00	35.00	35.00	35.00	35.00	35.00	35.00	35.00	35.00	35.00	35.00	35.00	35.00
35.00	35.00	35.00	35.00	35.00	35.00	35.00	35.00	35.00	35.00	35.00	35.00	35.00
35.00	35.00	35.00	35.00	35.00	35.00	35.00	35.00	35.00	35.00	35.00	35.00	35.00
35.00	35.00	35.00	35.00	35.00	35.00	35.00	35.00	35.00	35.00	35.00	35.00	35.00
35.00	35.00	35.00	35.00	35.00	35.00	35.00	35.00	35.00	35.00	35.00	35.00	35.00
35.00	35.00	35.00	35.00	35.00	35.00	35.00	35.00	35.00	35.00	35.00	35.00	35.00
35.00	35.00	35.00	35.00	35.00	34.67	35.00	35.00	34.98	35.00	35.00	35.00	35.00
35.00	35.00	35.00	35.00	35.00	35.00	35.00	35.00	35.00	35.00	35.00	35.00	35.00
35.00	35.00	35.00	35.00	35.00	35.00	35.00	35.00	35.00	35.00	35.00	35.00	35.00

Supplementary Table 1

35.00	35.00	35.00	35.00	35.00	35.00	35.00	35.00	35.00	35.00	35.00	35.00	35.00
35.00	35.00	35.00	35.00	35.00	35.00	35.00	35.00	35.00	35.00	34.84	35.00	34.31
35.00	35.00	35.00	35.00	35.00	35.00	35.00	35.00	34.99	35.00	35.00	35.00	35.00
35.00	35.00	35.00	35.00	35.00	35.00	35.00	35.00	35.00	35.00	35.00	35.00	35.00
32.92	32.96	32.78	34.28	34.77	33.94	35.00	34.47	34.29	32.58	34.87	35.00	35.00
35.00	35.00	35.00	35.00	35.00	35.00	35.00	35.00	35.00	35.00	35.00	35.00	35.00
35.00	35.00	35.00	35.00	35.00	35.00	35.00	35.00	34.76	35.00	35.00	35.00	35.00
35.00	35.00	35.00	34.53	35.00	34.93	35.00	35.00	34.97	35.00	34.78	35.00	35.00
30.54	31.60	29.88	30.34	29.94	29.79	29.81	29.55	30.28	29.81	30.36	29.53	29.91
35.00	35.00	35.00	34.35	34.62	33.95	35.00	35.00	34.45	35.00	34.17	35.00	35.00
35.00	33.39	32.72	33.06	33.68	31.27	31.64	31.76	32.86	31.86	31.74	31.81	32.31
35.00	35.00	35.00	34.44	35.00	35.00	35.00	35.00	34.97	35.00	35.00	35.00	34.92
29.09	30.31	29.06	29.06	28.78	27.56	27.95	28.68	28.79	28.53	29.42	28.16	28.92
35.00	35.00	35.00	35.00	35.00	35.00	35.00	35.00	34.99	35.00	35.00	35.00	34.97
35.00	35.00	35.00	35.00	35.00	35.00	35.00	35.00	35.00	35.00	35.00	35.00	35.00
35.00	35.00	35.00	35.00	35.00	35.00	35.00	35.00	35.00	35.00	35.00	35.00	35.00
28.38	28.17	28.61	27.62	28.49	27.27	27.34	27.39	27.91	27.72	28.24	27.27	27.60
27.90	27.96	27.55	27.46	27.90	26.85	26.75	27.09	27.33	26.96	27.72	26.47	26.76
35.00	35.00	35.00	35.00	35.00	35.00	35.00	35.00	35.00	35.00	35.00	35.00	35.00
28.01	27.68	28.08	28.06	28.30	28.15	28.11	28.28	28.23	28.15	28.68	27.50	28.16
35.00	35.00	35.00	35.00	35.00	35.00	35.00	35.00	35.00	35.00	35.00	35.00	35.00
35.00	35.00	35.00	35.00	35.00	35.00	35.00	34.86	34.99	35.00	35.00	35.00	34.49
35.00	35.00	35.00	35.00	35.00	35.00	35.00	35.00	35.00	35.00	35.00	35.00	35.00
35.00	35.00	35.00	35.00	35.00	35.00	35.00	35.00	35.00	35.00	35.00	35.00	35.00
35.00	35.00	35.00	35.00	35.00	35.00	35.00	35.00	35.00	35.00	35.00	35.00	35.00
35.00	35.00	35.00	35.00	35.00	35.00	35.00	35.00	35.00	35.00	35.00	35.00	35.00
35.00	35.00	35.00	35.00	35.00	35.00	35.00	35.00	34.97	35.00	35.00	35.00	35.00
32.73	33.31	32.03	35.00	35.00	33.11	35.00	35.00	33.95	33.63	34.08	35.00	35.00
28.09	28.59	28.59	27.52	28.49	28.31	27.50	28.05	27.93	27.36	28.00	26.02	27.30
34.00	35.00	33.48	34.32	35.00	35.00	35.00	34.25	34.29	32.65	31.49	34.11	33.31
35.00	35.00	32.35	32.34	32.23	31.26	31.91	31.38	32.49	30.90	31.70	31.01	32.99
35.00	35.00	35.00	35.00	35.00	35.00	35.00	35.00	35.00	35.00	35.00	35.00	35.00
35.00	35.00	35.00	35.00	35.00	35.00	35.00	35.00	35.00	35.00	35.00	35.00	35.00
35.00	35.00	35.00	35.00	35.00	35.00	35.00	35.00	35.00	35.00	35.00	35.00	35.00
35.00	35.00	35.00	35.00	35.00	35.00	35.00	35.00	35.00	35.00	35.00	35.00	35.00
35.00	35.00	35.00	35.00	35.00	35.00	35.00	35.00	35.00	35.00	35.00	35.00	35.00
35.00	35.00	34.82	35.00	35.00	35.00	35.00	35.00	34.99	35.00	35.00	35.00	34.44
35.00	35.00	35.00	35.00	35.00	35.00	35.00	35.00	35.00	35.00	35.00	35.00	35.00
35.00	35.00	35.00	35.00	35.00	33.10	35.00	33.85	34.67	35.00	34.74	34.27	35.00
30.67	30.44	30.40	30.86	30.82	30.17	30.61	30.50	30.68	31.44	31.34	30.18	30.87
35.00	35.00	34.82	35.00	35.00	35.00	35.00	35.00	34.99	34.22	34.49	35.00	35.00
35.00	35.00	35.00	35.00	35.00	35.00	35.00	35.00	35.00	35.00	35.00	35.00	35.00
25.50	27.88	30.23	27.09	27.18	26.20	26.91	27.67	26.81	27.66	27.08	24.32	28.49
35.00	35.00	35.00	35.00	35.00	35.00	35.00	35.00	34.99	34.67	34.31	35.00	35.00
34.43	34.84	31.02	31.48	31.58	30.18	31.44	30.81	31.62	30.46	31.24	30.59	31.18
35.00	35.00	34.22	35.00	35.00	35.00	35.00	35.00	34.96	34.92	33.69	35.00	35.00
35.00	35.00	35.00	35.00	35.00	35.00	35.00	35.00	35.00	35.00	35.00	35.00	35.00
35.00	35.00	35.00	35.00	35.00	35.00	35.00	35.00	35.00	35.00	35.00	35.00	34.88
35.00	35.00	35.00	35.00	35.00	35.00	34.39	35.00	34.95	34.82	35.00	35.00	35.00
33.01	32.11	32.95	35.00	34.23	34.22	35.00	33.09	34.10	32.35	35.00	35.00	35.00
35.00	34.79	35.00	35.00	35.00	35.00	35.00	35.00	34.97	35.00	35.00	35.00	35.00
33.27	32.70	35.00	35.00	34.57	34.09	33.46	32.86	34.22	34.72	35.00	33.72	33.49
35.00	35.00	35.00	35.00	35.00	33.59	34.83	35.00	34.91	35.00	35.00	35.00	35.00
32.07	33.99	35.00	35.00	35.00	35.00	35.00	34.54	33.70	32.48	33.69	34.55	35.00
35.00	35.00	35.00	35.00	35.00	35.00	35.00	35.00	35.00	35.00	35.00	35.00	35.00

Supplementary Table 1

35.00	35.00	35.00	35.00	35.00	35.00	35.00	35.00	35.00	35.00	35.00	35.00	35.00
35.00	35.00	35.00	35.00	35.00	35.00	35.00	35.00	35.00	35.00	35.00	35.00	35.00
35.00	35.00	35.00	35.00	35.00	35.00	35.00	35.00	34.90	35.00	35.00	34.66	35.00
35.00	35.00	35.00	35.00	35.00	35.00	35.00	35.00	35.00	35.00	35.00	35.00	35.00
33.47	35.00	35.00	35.00	35.00	35.00	35.00	35.00	34.85	35.00	35.00	35.00	35.00
29.63	30.19	32.73	31.24	30.72	31.49	32.26	31.65	31.19	32.81	30.73	31.98	33.69
35.00	35.00	35.00	33.73	35.00	33.05	34.92	34.83	34.49	35.00	34.72	33.59	34.94
35.00	34.79	35.00	35.00	35.00	34.48	35.00	34.78	34.90	35.00	35.00	35.00	35.00
33.52	33.61	32.71	33.16	34.80	34.27	34.06	34.32	33.60	33.37	34.42	33.67	35.00
35.00	35.00	35.00	35.00	35.00	35.00	35.00	35.00	35.00	35.00	35.00	35.00	35.00
34.73	35.00	35.00	35.00	35.00	35.00	35.00	35.00	34.93	35.00	35.00	35.00	35.00
29.26	28.95	29.86	30.06	30.53	30.73	31.51	30.29	30.10	29.35	31.12	30.49	31.28
35.00	35.00	35.00	35.00	35.00	35.00	35.00	35.00	34.99	35.00	35.00	35.00	35.00
35.00	35.00	35.00	35.00	35.00	35.00	35.00	35.00	35.00	35.00	35.00	35.00	35.00
34.10	35.00	32.82	34.03	35.00	33.96	33.80	33.74	34.15	34.58	34.93	34.61	35.00
35.00	35.00	34.96	35.00	35.00	35.00	35.00	35.00	34.91	35.00	35.00	35.00	35.00
26.69	27.31	25.92	26.22	26.25	25.78	26.03	26.28	26.56	26.48	27.35	25.98	26.70
33.42	35.00	35.00	34.40	35.00	34.45	34.48	35.00	34.64	35.00	35.00	35.00	35.00
31.36	30.65	31.71	32.57	32.62	31.55	32.06	31.34	31.79	31.50	32.73	32.24	32.73
35.00	35.00	35.00	35.00	35.00	35.00	35.00	35.00	35.00	35.00	35.00	35.00	35.00
33.27	33.13	33.38	35.00	35.00	35.00	35.00	35.00	34.48	34.35	35.00	34.44	35.00
35.00	35.00	35.00	35.00	35.00	35.00	34.50	34.74	34.64	35.00	35.00	35.00	34.02
33.37	35.00	35.00	35.00	35.00	35.00	35.00	35.00	34.91	35.00	35.00	35.00	35.00
33.41	35.00	35.00	35.00	35.00	34.20	34.40	35.00	34.56	35.00	34.23	35.00	34.17
35.00	35.00	35.00	35.00	35.00	35.00	35.00	35.00	35.00	35.00	35.00	35.00	35.00
35.00	35.00	35.00	35.00	35.00	35.00	35.00	35.00	35.00	35.00	35.00	35.00	35.00
35.00	33.24	35.00	35.00	35.00	33.66	35.00	35.00	34.58	34.79	33.36	34.05	35.00
25.61	25.44	25.33	25.32	25.28	24.54	24.92	25.05	25.16	24.91	25.35	24.26	24.86
35.00	35.00	35.00	35.00	35.00	35.00	34.56	35.00	34.92	35.00	35.00	35.00	35.00
35.00	35.00	35.00	35.00	35.00	35.00	35.00	35.00	35.00	35.00	35.00	35.00	35.00
35.00	35.00	35.00	35.00	35.00	35.00	35.00	35.00	35.00	35.00	35.00	35.00	35.00
25.77	25.85	25.61	25.43	25.31	24.87	25.29	25.49	25.41	24.89	25.71	24.51	25.30
35.00	35.00	35.00	35.00	35.00	35.00	35.00	35.00	35.00	35.00	35.00	35.00	35.00
34.64	33.98	35.00	33.53	34.05	34.11	34.03	34.72	34.23	33.91	34.99	33.91	33.75
35.00	35.00	35.00	35.00	35.00	35.00	35.00	35.00	34.98	35.00	35.00	35.00	35.00
35.00	35.00	35.00	35.00	35.00	35.00	35.00	35.00	35.00	35.00	35.00	35.00	35.00
31.77	32.96	35.00	32.75	34.14	34.79	33.02	33.10	34.02	35.00	35.00	35.00	35.00
32.46	33.43	33.57	35.00	35.00	35.00	35.00	33.77	34.35	32.62	35.00	34.54	35.00
35.00	35.00	35.00	35.00	35.00	35.00	35.00	35.00	35.00	35.00	35.00	35.00	35.00
35.00	35.00	35.00	35.00	35.00	35.00	35.00	35.00	35.00	35.00	35.00	35.00	35.00
35.00	35.00	35.00	35.00	35.00	35.00	35.00	35.00	35.00	35.00	35.00	35.00	35.00
35.00	35.00	35.00	35.00	35.00	35.00	35.00	35.00	35.00	35.00	35.00	35.00	35.00
35.00	35.00	35.00	35.00	35.00	35.00	35.00	35.00	35.00	35.00	35.00	35.00	35.00
35.00	35.00	35.00	35.00	35.00	35.00	35.00	35.00	35.00	35.00	35.00	35.00	35.00

Supplementary Table 1

23	24	25	26	27	28	29	30	31	32	33	34	35	control mean	delta Ct
26.05	23.83	26.03	24.82	24.65	24.55	24.51	24.12	23.49	24.51	24.71	24.99	25.27	24.75	0.25
24.02	23.08	24.47	23.38	23.99	23.24	23.20	21.32	22.16	22.79	22.48	23.84	23.87	23.23	0.16
26.15	23.96	24.66	23.96	23.97	23.57	22.99	22.18	21.87	23.39	23.59	23.65	24.25	23.74	0.17
28.52	26.93	27.69	26.88	26.78	27.15	26.90	26.03	25.63	26.47	26.82	26.85	27.32	26.90	0.15
29.43	26.78	28.68	28.66	28.84	28.60	28.55	28.83	27.02	28.12	27.96	27.84	28.43	28.16	0.39
25.62	23.21	24.09	23.49	23.73	23.66	23.10	22.14	22.08	23.21	23.47	23.31	23.54	23.46	0.14
34.15	29.05	31.69	31.96	30.90	33.24	32.41	31.58	31.66	33.96	32.66	34.13	33.27	31.88	0.97
35.00	34.93	35.00	35.00	35.00	35.00	35.00	35.00	35.00	35.00	35.00	35.00	35.00	35.00	0.00
31.77	29.83	33.11	32.04	29.36	31.83	33.54	30.20	30.17	30.59	30.69	31.58	31.32	31.17	0.54
30.59	29.97	30.07	28.58	29.41	29.74	29.34	28.25	27.74	28.63	29.55	28.86	29.49	29.34	0.39
27.11	24.74	24.85	24.44	24.15	25.09	23.84	24.43	22.97	24.63	24.89	24.09	24.97	24.73	0.15
30.61	30.86	32.13	30.32	30.75	30.53	31.76	28.76	29.54	30.06	30.18	30.46	29.66	30.46	0.44
26.94	26.52	27.00	25.84	26.25	25.95	26.54	23.85	24.73	25.42	25.65	25.38	25.60	25.96	0.26
24.37	23.95	24.07	22.99	23.15	23.56	23.95	21.65	22.27	22.73	23.30	23.24	23.18	23.31	0.38
26.55	25.82	27.30	26.43	26.22	26.49	27.18	24.76	25.94	26.12	26.19	26.54	25.79	26.17	0.40
23.89	23.00	23.22	22.36	22.55	22.81	22.40	21.29	21.11	22.38	22.32	22.11	22.66	22.51	0.23
30.98	28.19	29.62	29.36	28.53	30.64	29.28	28.51	27.99	28.49	29.05	29.60	29.46	29.25	0.58
31.96	31.66	33.58	32.04	31.62	32.52	35.00	31.01	33.09	31.15	32.13	31.45	31.94	32.37	0.57
31.84	32.46	34.44	31.48	31.59	31.94	35.00	29.95	32.59	31.01	31.88	32.63	31.31	32.31	0.55
24.00	24.17	25.07	23.79	24.21	24.48	24.89	22.42	23.22	23.56	23.51	24.04	23.89	23.92	0.17
26.07	25.16	25.27	24.72	24.19	25.18	24.91	23.76	23.37	24.52	24.66	24.64	25.12	24.70	0.31
31.12	27.61	27.98	28.00	28.00	28.77	28.06	29.63	26.96	28.06	28.67	27.37	28.09	28.24	0.14
35.00	34.54	35.00	35.00	35.00	34.86	35.00	35.00	35.00	33.76	35.00	34.02	34.16	34.76	0.04
26.62	25.12	26.81	24.76	25.63	25.61	26.66	26.85	24.07	25.58	25.83	25.84	25.59	25.60	0.64
30.40	28.44	29.36	29.23	28.46	29.44	27.04	28.44	27.51	29.29	29.53	29.43	29.29	28.49	0.65
28.88	26.57	26.74	26.21	26.65	26.74	26.09	26.07	24.64	26.21	26.41	26.25	26.60	26.48	0.37
31.69	28.40	28.91	29.74	29.26	29.66	28.68	27.85	27.26	29.18	28.77	29.72	29.75	29.12	0.93
29.82	28.24	29.89	29.74	28.41	29.65	29.44	27.91	27.65	29.18	28.54	28.50	28.75	28.96	0.25
25.64	23.74	23.99	22.95	22.97	23.52	22.72	21.38	21.44	22.72	22.85	22.78	23.40	23.22	0.15
32.14	28.89	30.46	30.68	30.80	31.63	32.47	29.22	29.21	29.32	32.72	30.39	30.50	30.60	0.25
34.13	29.08	35.00	35.00	35.00	35.00	35.00	32.25	35.00	34.83	35.00	35.00	35.00	34.12	0.70
26.16	22.85	23.68	23.02	23.42	23.28	23.62	23.47	21.71	23.43	23.73	22.93	23.50	23.38	0.39
26.85	23.71	24.66	24.40	24.48	24.83	24.07	24.73	22.54	24.93	25.22	23.41	24.92	24.42	0.27
24.83	23.58	24.99	24.06	23.48	24.32	24.04	23.75	22.24	23.96	23.09	23.90	24.31	23.87	0.24
27.84	26.12	26.60	25.84	26.31	26.17	27.58	26.55	25.53	25.91	26.69	25.92	25.96	26.40	0.47
28.68	27.39	29.13	28.42	28.11	29.53	29.35	27.65	27.84	28.44	28.32	28.22	28.77	28.23	0.18
35.00	27.89	35.00	35.00	35.00	35.00	35.00	35.00	33.49	35.00	35.00	30.44	35.00	34.12	0.55
32.96	30.57	31.83	30.54	30.54	31.43	30.08	31.50	32.59	30.75	30.57	30.40	30.87	30.95	-0.10
32.52	29.48	30.93	30.40	30.75	31.43	30.06	35.00	29.50	32.04	31.90	30.34	31.53	30.97	0.45
25.78	25.25	25.48	24.55	24.57	25.03	25.55	23.61	23.51	24.16	24.58	24.55	24.73	24.67	0.38
22.01	19.25	20.07	19.59	20.05	20.44	19.76	19.74	18.45	20.07	20.60	19.19	20.12	19.86	0.31
25.24	21.93	22.13	21.42	21.92	22.07	22.40	22.05	20.46	22.04	22.68	21.39	22.04	22.07	0.43
27.26	25.03	24.88	24.37	24.05	24.91	24.42	23.90	23.22	24.41	24.65	23.96	25.01	24.66	0.28
23.27	23.80	23.98	22.59	23.00	23.04	23.90	21.42	21.92	22.10	22.13	23.05	23.09	23.00	0.12
25.49	24.77	24.85	23.80	24.26	24.51	25.40	23.70	23.04	23.90	24.22	24.41	24.78	24.38	0.31
35.00	29.42	35.00	35.00	33.77	35.00	35.00	35.00	35.00	35.00	35.00	35.00	35.00	34.42	0.21
30.42	28.16	28.80	28.87	28.95	28.98	28.97	27.88	26.73	28.67	28.73	28.70	28.68	28.64	0.51
30.17	28.11	28.47	28.31	27.23	28.25	26.89	27.76	27.44	28.40	28.98	27.25	27.26	28.14	0.29
21.58	19.92	20.08	19.16	19.65	19.88	19.22	18.40	18.04	19.22	19.31	19.27	19.67	19.53	0.20
24.33	23.39	23.96	22.98	23.12	23.59	23.45	22.95	22.60	22.87	23.45	23.09	23.17	23.32	0.24

Supplementary Table 1

27.00	25.32	25.81	25.11	25.40	25.56	26.07	25.07	23.95	24.85	25.26	25.03	25.24	25.38	0.45
25.76	25.38	25.76	24.52	24.98	24.91	25.46	23.68	23.52	24.15	24.43	24.62	24.72	24.82	0.19
25.85	23.59	24.16	23.64	23.97	24.26	23.72	24.29	22.35	23.85	24.11	22.93	23.95	23.84	0.19
24.96	22.89	22.84	22.21	22.47	22.96	21.91	22.10	20.75	22.16	22.29	21.66	22.19	22.46	0.12
22.51	20.84	20.90	20.29	20.66	20.78	20.01	19.92	19.08	20.31	20.28	19.99	20.24	20.47	0.14
28.78	28.12	27.82	27.03	27.05	27.43	26.61	25.12	25.63	26.91	27.28	27.10	27.34	27.09	0.17
35.00	35.00	35.00	34.98	35.00	35.00	35.00	35.00	35.00	35.00	35.00	34.16	35.00	34.95	0.00
29.89	28.62	28.51	28.34	27.87	28.81	29.71	28.04	27.78	28.45	28.80	28.39	28.15	28.52	0.12
25.18	22.32	23.28	22.52	22.41	23.27	22.20	22.57	21.12	22.73	23.11	21.90	23.04	22.56	0.31
31.64	30.38	32.28	30.35	29.66	32.43	31.54	31.06	31.42	31.19	31.42	31.00	31.30	30.97	0.08
35.00	35.00	35.00	35.00	35.00	35.00	35.00	35.00	35.00	35.00	35.00	35.00	35.00	35.00	0.00
35.00	35.00	35.00	35.00	35.00	35.00	35.00	35.00	35.00	35.00	35.00	35.00	35.00	35.00	0.00
35.00	34.49	34.96	35.00	33.35	35.00	33.99	35.00	35.00	34.76	35.00	32.76	35.00	34.60	0.06
28.10	25.02	26.01	25.41	25.33	26.10	27.22	26.28	25.29	26.13	26.72	26.17	26.14	26.02	0.58
35.00	33.86	35.00	35.00	34.22	35.00	33.03	35.00	31.84	35.00	35.00	35.00	35.00	34.59	-0.17
28.98	27.02	28.61	27.83	27.37	27.98	27.52	27.23	27.45	27.69	28.02	27.43	28.22	27.74	0.32
28.41	26.04	27.05	26.18	26.50	27.14	26.64	25.05	25.19	26.25	26.54	26.34	26.77	26.51	0.16
34.32	30.09	31.25	30.66	30.24	32.22	31.08	32.63	30.24	30.92	32.88	31.12	31.34	31.37	0.04
35.00	31.14	31.90	32.87	32.01	35.00	32.68	31.23	30.60	33.22	32.15	33.11	34.51	32.79	0.22
29.04	26.50	27.21	27.27	26.87	27.20	26.92	26.34	26.10	27.03	27.33	27.09	26.91	27.04	0.47
32.02	28.23	30.83	29.65	28.73	31.60	32.29	29.28	29.27	30.58	32.53	30.73	30.03	29.90	-0.17
33.11	30.87	35.00	31.35	35.00	31.57	30.36	31.31	30.39	33.75	35.00	35.00	33.34	32.92	0.47
35.00	30.26	35.00	35.00	35.00	35.00	35.00	35.00	35.00	35.00	35.00	35.00	35.00	34.57	0.35
33.80	30.47	33.14	34.47	34.10	34.36	34.24	30.65	29.69	31.70	32.50	31.82	33.81	32.62	0.74
34.07	31.63	35.00	35.00	35.00	35.00	35.00	35.00	35.00	35.00	33.57	35.00	35.00	34.45	0.35
35.00	29.37	35.00	33.41	33.46	35.00	24.63	32.59	34.66	30.07	35.00	34.56	35.00	33.23	1.36
35.00	35.00	35.00	35.00	35.00	35.00	35.00	35.00	35.00	35.00	35.00	35.00	35.00	34.98	0.02
35.00	32.23	35.00	32.32	33.48	33.37	35.00	35.00	32.49	35.00	31.07	35.00	34.74	33.61	0.72
35.00	35.00	35.00	35.00	35.00	35.00	35.00	35.00	35.00	35.00	34.17	35.00	35.00	34.85	0.15
34.19	29.17	31.29	31.13	30.17	31.71	35.00	34.71	27.92	32.71	30.69	32.59	32.43	31.60	0.60
35.00	35.00	35.00	35.00	35.00	35.00	35.00	35.00	35.00	35.00	35.00	35.00	35.00	34.83	0.17
35.00	31.38	35.00	34.96	31.37	32.10	34.91	31.30	30.74	35.00	32.73	34.25	33.05	33.20	0.32
35.00	32.79	35.00	34.58	35.00	35.00	35.00	35.00	35.00	35.00	35.00	35.00	35.00	34.61	0.12
35.00	34.45	35.00	35.00	34.71	33.02	35.00	29.94	31.88	35.00	35.00	35.00	34.78	33.95	0.01
35.00	32.82	35.00	34.30	35.00	35.00	35.00	35.00	35.00	35.00	35.00	35.00	35.00	34.56	0.40
35.00	35.00	35.00	35.00	35.00	35.00	35.00	35.00	35.00	35.00	35.00	35.00	35.00	35.00	0.00
35.00	31.63	35.00	35.00	32.86	35.00	35.00	35.00	35.00	35.00	35.00	35.00	35.00	34.68	0.30
35.00	29.01	35.00	35.00	30.63	35.00	35.00	35.00	35.00	35.00	35.00	35.00	35.00	34.39	0.58
35.00	35.00	35.00	35.00	35.00	35.00	35.00	35.00	35.00	35.00	35.00	35.00	35.00	34.85	0.08
35.00	31.30	35.00	35.00	35.00	35.00	35.00	35.00	35.00	35.00	35.00	35.00	35.00	34.78	0.22
35.00	31.06	34.81	35.00	35.00	35.00	35.00	35.00	35.00	35.00	35.00	35.00	35.00	34.71	0.24
35.00	33.06	34.24	35.00	35.00	35.00	35.00	35.00	35.00	35.00	35.00	33.55	35.00	34.53	0.08
35.00	35.00	35.00	35.00	35.00	35.00	35.00	31.35	35.00	35.00	35.00	35.00	35.00	34.79	0.13
28.40	26.14	25.99	25.58	25.35	25.96	25.40	26.07	23.67	25.12	25.86	25.35	26.19	25.76	0.20
25.29	22.79	22.79	22.02	22.47	23.01	23.09	22.04	21.27	22.31	22.89	22.61	22.57	22.77	0.21
24.83	22.28	23.34	22.74	22.86	22.90	22.92	22.89	21.27	22.57	22.97	21.57	22.36	22.69	0.24
27.18	24.73	24.43	24.02	23.60	24.91	24.57	23.45	23.07	24.10	24.59	24.36	24.32	24.47	0.24
27.81	25.49	26.33	25.45	25.20	25.86	25.42	26.23	24.15	25.36	25.97	25.60	25.95	25.75	0.14
28.46	25.66	25.56	25.33	25.27	25.89	25.15	24.64	23.81	25.37	25.66	24.90	25.77	25.60	0.19
23.89	23.21	23.61	22.72	22.60	23.31	22.64	21.52	21.73	22.76	22.85	22.78	22.73	22.82	0.17
28.61	26.08	26.85	25.89	25.96	26.79	27.10	25.50	25.56	26.08	26.99	26.17	26.14	26.47	0.47
27.53	24.67	24.73	24.56	24.71	25.34	24.15	22.98	22.81	24.88	24.88	23.79	24.73	24.70	0.17
25.84	23.30	23.52	23.23	23.09	23.91	23.06	22.43	22.09	23.38	23.57	22.81	23.48	23.33	0.18
24.64	21.67	22.04	21.22	21.13	21.48	20.74	20.67	19.34	21.75	21.99	20.69	22.30	21.56	0.24
31.70	26.90	29.87	29.07	28.58	29.47	28.12	29.85	28.52	29.04	30.24	28.95	28.55	29.21	0.28

Supplementary Table 1

28.29	24.59	25.42	24.77	24.23	25.22	24.01	23.68	23.51	25.37	25.50	24.16	25.33	24.92	0.36
32.05	28.58	29.05	28.40	28.23	28.33	27.14	27.26	26.47	28.60	28.52	27.42	28.23	28.46	0.26
31.35	27.98	28.93	28.77	28.64	29.66	28.93	29.68	27.59	28.97	30.38	29.15	28.87	28.97	0.48
28.76	27.09	27.78	27.33	27.77	29.10	28.29	28.71	26.71	27.61	28.72	26.61	27.26	27.70	0.44
33.18	29.60	30.77	29.28	29.48	30.20	30.80	29.38	28.81	28.97	30.02	29.69	29.51	30.14	0.59
28.95	27.85	27.74	27.13	27.19	28.01	28.11	26.22	26.08	26.84	27.35	27.27	27.87	27.51	0.33
30.28	27.65	29.32	28.67	29.29	28.89	28.88	27.60	27.65	28.54	28.78	29.33	28.94	28.79	0.33
30.46	26.81	27.63	27.08	27.40	28.39	26.40	26.91	26.73	26.58	27.80	27.01	28.34	27.50	0.27
27.05	24.72	24.82	24.59	24.94	25.76	24.86	24.26	23.11	24.73	25.27	25.06	24.74	24.85	0.18
35.00	34.32	35.00	35.00	35.00	32.24	33.40	35.00	35.00	32.00	34.50	34.60	34.15	34.25	-0.25
31.25	29.80	31.39	29.21	29.04	35.00	33.75	25.58	33.06	33.44	28.55	29.44	29.61	30.52	0.75
33.05	33.04	33.61	32.20	35.00	31.01	35.00	35.00	30.25	35.00	35.00	34.01	35.00	33.69	0.14
34.83	34.37	33.95	35.00	35.00	35.00	35.00	35.00	35.00	35.00	35.00	35.00	35.00	34.47	0.40
29.38	27.28	29.51	28.99	28.41	29.52	28.80	27.30	28.41	28.62	28.72	28.37	28.87	28.68	0.24
28.32	25.93	25.90	25.44	25.63	26.32	25.40	24.71	24.90	25.99	26.99	24.97	25.83	25.84	0.39
35.00	35.00	35.00	35.00	35.00	35.00	35.00	35.00	35.00	35.00	35.00	35.00	35.00	35.00	0.00
29.54	25.95	26.43	25.84	25.92	26.50	26.14	25.84	24.86	26.61	26.46	25.60	26.05	26.33	0.27
35.00	31.61	31.57	32.03	31.28	31.54	34.13	32.04	30.38	31.56	31.24	32.15	32.11	32.02	0.09
29.07	26.38	26.58	26.45	26.26	26.80	25.61	25.74	24.23	26.68	26.44	25.82	26.34	26.37	0.30
35.00	35.00	35.00	35.00	35.00	35.00	35.00	35.00	35.00	35.00	34.88	35.00	35.00	34.99	-0.21
25.05	22.58	23.23	22.73	22.55	23.39	23.24	23.12	21.35	22.68	23.31	22.07	22.88	22.90	0.29
30.79	28.63	29.42	29.03	29.13	30.07	28.86	28.80	28.06	29.36	30.08	28.68	28.72	29.18	0.26
35.00	32.55	33.58	32.64	34.33	33.69	32.77	35.00	31.47	35.00	35.00	33.93	35.00	33.63	0.49
31.81	27.58	29.24	28.45	28.53	29.69	28.47	28.66	27.98	29.70	29.59	27.76	28.64	28.84	0.17
29.04	25.65	26.50	25.88	25.92	26.96	26.25	25.94	25.07	26.10	26.40	25.92	26.28	26.31	0.12
33.93	30.43	31.74	30.69	30.40	30.98	30.17	32.52	28.99	31.32	32.15	30.50	30.69	31.05	0.16
29.33	25.86	26.47	26.40	26.18	26.55	25.79	25.00	24.69	26.03	26.18	25.85	27.03	26.22	0.20
27.80	24.70	24.81	24.49	24.68	25.35	24.71	24.26	23.22	24.69	25.03	24.13	24.91	24.77	0.10
31.38	28.19	28.88	28.96	28.68	29.39	29.11	31.39	27.55	29.32	29.70	28.43	28.64	28.99	0.06
35.00	30.86	31.85	31.38	31.26	31.14	31.47	32.23	29.62	30.39	30.86	32.67	31.24	31.59	1.01
34.36	31.34	32.90	32.17	32.17	33.99	34.08	35.00	32.09	32.73	35.00	32.14	32.76	32.90	0.90
35.00	35.00	35.00	35.00	35.00	35.00	35.00	35.00	35.00	35.00	35.00	35.00	35.00	35.00	-0.02
35.00	30.16	33.33	31.92	34.65	35.00	35.00	35.00	32.52	35.00	35.00	34.89	35.00	34.19	-0.05
35.00	35.00	35.00	35.00	35.00	35.00	35.00	35.00	35.00	35.00	34.61	35.00	35.00	34.98	0.02
27.48	23.67	23.80	23.52	23.83	23.91	23.33	23.63	21.95	24.17	25.10	23.09	24.11	23.90	0.38
26.95	26.30	27.44	25.94	26.77	26.63	25.96	24.91	25.05	25.98	25.90	26.16	26.30	26.16	0.08
29.51	28.54	28.09	27.68	28.31	28.91	27.99	26.85	26.72	28.08	28.59	27.77	28.05	28.06	0.18
28.83	28.58	29.14	27.41	27.98	28.44	29.41	26.38	27.05	27.68	28.40	28.45	27.67	28.18	0.23
31.60	28.42	28.13	27.81	27.51	28.50	27.87	26.89	26.20	28.40	28.37	27.30	28.28	28.10	0.12
35.00	35.00	35.00	35.00	35.00	35.00	35.00	35.00	35.00	35.00	35.00	35.00	35.00	35.00	0.00
26.61	23.85	24.57	24.67	24.51	25.15	24.15	23.57	23.02	24.91	24.73	23.61	24.51	24.31	-0.01
32.11	28.74	28.68	28.74	28.21	29.10	28.80	28.36	27.77	28.90	29.09	27.98	28.47	28.81	0.31
27.01	25.80	26.20	25.93	25.73	26.34	25.73	25.13	24.32	25.77	25.99	25.49	25.95	25.79	0.29
32.64	29.08	28.97	28.14	28.42	29.00	28.77	28.08	26.33	28.34	29.66	27.48	28.02	28.64	0.19
29.59	28.73	29.14	28.66	28.34	28.72	29.83	28.79	27.62	29.13	29.35	27.67	28.57	28.76	0.48
32.57	32.12	31.64	31.93	31.28	30.70	33.58	32.44	30.14	29.90	31.47	32.96	30.45	31.51	-0.03
35.00	34.17	35.00	35.00	34.58	35.00	35.00	35.00	35.00	35.00	35.00	33.10	35.00	34.81	0.19
35.00	35.00	35.00	35.00	33.59	35.00	33.41	35.00	35.00	34.75	35.00	35.00	35.00	34.74	-0.07
27.19	25.36	25.60	25.24	25.40	26.68	25.80	24.21	24.19	25.70	25.86	25.26	25.80	25.52	0.17
35.00	35.00	35.00	35.00	34.82	35.00	35.00	35.00	33.20	35.00	35.00	35.00	35.00	34.88	0.02
35.00	35.00	35.00	35.00	35.00	35.00	35.00	35.00	35.00	35.00	35.00	35.00	35.00	35.00	0.00
29.37	29.06	29.20	27.73	28.65	28.50	28.09	26.15	26.71	28.64	27.86	28.17	28.33	28.26	0.01
32.19	29.27	29.92	29.50	29.86	30.24	29.03	28.54	27.16	29.40	30.32	29.65	29.96	29.56	0.47
35.00	35.00	35.00	35.00	35.00	35.00	35.00	35.00	35.00	35.00	35.00	35.00	35.00	35.00	0.00
27.03	26.46	26.79	26.17	26.14	27.00	26.59	24.36	24.99	25.40	25.57	26.60	27.09	26.20	0.27

Supplementary Table 1

35.00	35.00	35.00	35.00	35.00	35.00	35.00	35.00	35.00	35.00	35.00	35.00	35.00	35.00	0.00
31.63	31.27	31.61	30.66	31.06	31.65	31.82	29.30	30.05	32.18	30.74	30.09	30.67	30.93	0.05
26.24	23.79	25.46	24.82	24.82	26.05	24.87	24.39	23.79	25.46	25.30	24.53	25.46	24.73	0.22
25.85	23.47	24.18	23.67	23.81	24.63	23.77	23.98	22.47	24.03	24.35	23.28	24.14	23.88	0.26
35.00	34.38	35.00	35.00	35.00	35.00	35.00	35.00	35.00	35.00	35.00	35.00	35.00	34.92	-0.02
30.90	30.41	32.46	31.11	30.01	32.66	30.99	29.61	29.44	30.79	30.12	31.12	31.14	30.85	0.06
28.17	27.05	27.49	26.90	26.74	27.36	26.61	26.36	25.82	27.04	27.03	26.52	26.65	26.95	0.07
25.56	23.59	23.67	23.32	23.71	23.98	23.07	22.85	21.63	23.18	23.51	22.75	23.53	23.43	0.21
28.33	26.22	26.96	26.60	26.62	27.23	26.50	26.42	25.25	26.48	26.76	26.37	26.94	26.63	0.23
26.52	25.17	25.67	25.09	25.30	25.64	25.47	24.24	24.21	25.34	25.41	24.67	25.36	25.29	0.27
35.00	33.62	35.00	35.00	35.00	35.00	35.00	35.00	35.00	35.00	35.00	35.00	35.00	34.92	0.08
33.64	29.80	32.91	34.54	32.00	32.53	35.00	28.19	29.97	31.45	31.30	33.41	31.96	32.12	0.34
28.47	27.04	27.83	27.85	27.78	27.67	27.24	26.62	26.79	28.11	28.33	27.29	28.03	27.50	0.18
26.60	26.31	26.64	25.83	25.76	26.59	26.42	23.61	24.63	25.81	25.27	25.90	25.73	25.84	0.04
34.66	31.45	32.14	33.51	31.84	31.78	32.60	32.51	31.12	32.52	33.86	32.11	31.21	32.15	0.09
35.00	32.41	34.59	34.63	33.04	35.00	34.34	31.20	32.94	35.00	34.65	33.88	34.42	33.90	0.40
32.68	31.64	33.89	31.75	32.49	33.89	32.15	32.29	31.90	32.20	32.85	31.71	31.87	32.27	-0.10
27.64	25.75	26.59	26.10	26.13	26.93	25.81	26.31	25.53	26.80	27.11	25.28	26.16	26.19	0.25
30.01	28.56	28.24	27.59	27.78	28.28	27.85	26.42	26.36	27.44	27.70	27.51	28.02	27.89	0.18
33.53	32.92	34.38	34.50	34.21	33.15	35.00	31.79	31.63	34.68	34.18	34.02	35.00	33.82	-0.41
29.82	26.43	26.74	26.32	26.17	27.05	26.75	25.77	25.48	26.68	26.80	25.95	26.65	26.63	0.14
35.00	30.94	33.31	32.76	32.74	33.55	31.27	31.27	30.94	34.83	35.00	32.62	32.85	32.96	0.62
33.67	32.20	32.14	33.00	34.99	35.00	32.31	35.00	33.71	32.71	31.86	32.66	32.84	32.90	0.71
34.31	33.25	33.53	32.01	33.67	35.00	32.42	32.80	30.86	34.92	32.16	32.05	33.46	33.31	0.14
29.79	29.79	29.79	29.79	29.79	29.79	29.79	29.79	29.79	29.79	29.79	29.79	29.79	29.79	0.00
28.93	26.28	27.39	27.12	26.57	27.14	26.97	25.50	25.85	27.19	27.32	26.80	27.34	26.92	0.19
35.00	34.26	35.00	32.35	32.17	35.00	33.48	35.00	32.67	34.32	35.00	35.00	33.38	34.06	0.05
26.59	26.10	26.10	25.40	25.89	26.05	26.61	25.11	24.79	25.74	25.92	25.33	25.47	25.73	0.20
27.11	25.89	26.69	26.00	26.22	26.81	25.67	24.50	25.14	26.23	26.97			26.14	0.21
25.31	22.87	22.30	21.54	22.09	22.71	24.26	22.59	20.98	21.74	22.37	22.19	22.32	22.54	0.24
24.92	22.28	22.37	21.92	21.94	22.83	22.11	20.97	20.30	22.12	22.59	21.58	22.00	22.16	0.16
32.17	29.06	29.97	29.96	29.80	30.23	28.93	29.47	28.68	30.56	30.60	28.69	29.76	29.72	0.31
31.37	27.84	28.85	28.66	28.20	29.51	29.20	28.22	27.30	28.97	29.31	27.65	28.87	28.68	0.26
28.76	26.38	26.16	25.45	25.53	26.24	26.73	25.83	24.33	25.54	26.28	25.39	26.52	26.06	0.39
29.66	28.03	28.40	27.41	27.56	27.98	28.24	27.13	25.79	27.47	27.95	26.82	27.92	27.79	0.21
30.34	27.20	28.52	28.14	27.67	29.53	27.90	28.70	26.64	28.34	29.29	26.68	28.27	27.99	0.08
25.58	24.73	24.53	23.86	23.68	24.36	24.66	23.27	22.59	24.06	24.08	23.74	23.98	24.13	0.21
31.55	28.73	29.21	29.43	29.49	29.89	30.02	28.79	26.63	28.47	29.69	28.61	28.56	29.25	0.36
30.43	27.79	28.97	28.57	28.11	29.46	28.00	27.74	26.88	28.50	29.27	28.12	28.45	28.42	0.24
22.95	22.10	21.74	21.04	21.40	21.99	22.04	19.76	19.63	21.29	21.43	21.35	21.75	21.47	0.18
35.00	35.00	35.00	35.00	34.15	35.00	35.00	35.00	35.00	35.00	35.00	35.00	35.00	34.95	0.05
34.57	33.27	35.00	34.43	35.00	35.00	35.00	35.00	35.00	35.00	35.00	35.00	33.66	34.59	-0.26
32.39	31.22	32.66	31.62	33.18	32.18	35.00	32.63	30.90	32.80	31.54	31.77	31.83	32.36	0.57
35.00	35.00	35.00	35.00	35.00	35.00	35.00	35.00	35.00	35.00	35.00	34.91	35.00	34.99	0.01
26.49	23.56	23.83	23.27	23.38	24.29	23.78	22.07	22.01	23.23	23.90	22.89	23.68	23.61	0.23
31.61	32.70	31.74	32.29	30.78	30.80	33.65	31.18	30.44	32.04	32.19	31.61	31.54	31.64	0.01
35.00	34.74	35.00	35.00	35.00	35.00	35.00	30.33	30.57	35.00	35.00	35.00	35.00	34.38	0.30
35.00	35.00	35.00	35.00	35.00	35.00	35.00	35.00	35.00	35.00	35.00	35.00	35.00	35.00	0.00
28.36	26.81	26.35	25.33	25.38	25.47	26.40	24.41	23.86	25.43	26.12	25.71	25.70	25.96	0.11
31.97	29.36	31.30	30.81	30.56	31.79	30.63	31.28	29.58	30.75	31.67	30.16	30.60	30.56	0.25
35.00	33.15	33.86	33.02	32.57	33.02	35.00	35.00	35.00	35.00	35.00	33.47	35.00	34.10	0.22
23.64	21.62	21.89	21.11	21.20	22.22	22.26	20.97	20.47	21.10	21.64	21.09	21.55	21.62	0.22
24.70	22.98	23.24	22.59	22.70	23.43	23.95	22.53	21.81	22.59	23.25	22.48	23.07	23.02	0.29
35.00	34.63	35.00	35.00	35.00	35.00	35.00	35.00	35.00	35.00	33.65	35.00	33.92	34.84	0.09
35.00	32.06	33.52	34.64	34.76	34.99	34.48	31.22	35.00	33.22	32.99	35.00	35.00	34.05	0.63

Supplementary Table 1

35.00	33.83	35.00	35.00	35.00	35.00	35.00	35.00	35.00	35.00	35.00	35.00	35.00	34.93	0.07
35.00	35.00	35.00	35.00	33.45	33.66	35.00	35.00	35.00	35.00	33.62	35.00	35.00	34.71	-0.08
35.00	33.35	35.00	33.58	32.29	32.63	35.00	35.00	32.13	33.49	34.01	31.62	33.55	33.23	0.31
33.71	33.76	32.67	32.04	32.40	33.44	35.00	35.00	31.70	35.00	33.56	32.50	32.74	33.07	0.70
31.68	30.14	31.31	30.65	29.45	30.63	31.03	29.86	29.54	30.71	31.32	30.96	31.31	30.64	0.31
34.35	32.71	33.72	31.94	30.88	33.54	31.88	29.93	32.09	33.25	34.25	30.87	31.27	32.29	0.69
30.82	27.46	28.00	27.84	27.70	28.19	28.13	27.25	26.22	28.54	28.48	27.45	28.19	28.11	0.17
35.00	35.00	33.73	33.54	35.00	34.23	35.00	35.00	35.00	35.00	35.00	35.00	35.00	34.79	0.17
33.94	29.57	31.09	30.96	30.64	31.50	31.89	29.72	28.59	31.27	31.07	31.40	31.41	30.99	0.25
29.28	26.75	26.52	26.04	26.18	26.50	26.24	26.27	24.35	26.33	26.49	25.70	26.35	26.45	0.21
35.00	34.69	35.00	35.00	35.00	35.00	34.74	32.64	35.00	35.00	35.00	34.76	34.17	34.70	0.26
35.00	35.00	34.64	35.00	35.00	35.00	35.00	35.00	34.87	34.44	35.00	33.71	35.00	34.76	-0.17
35.00	35.00	35.00	35.00	35.00	35.00	35.00	35.00	35.00	35.00	35.00	35.00	35.00	35.00	0.00
24.93	22.22	22.86	22.78	22.53	24.82	25.26	22.74	21.83	23.51	24.42	22.48	22.85	23.12	0.28
24.11	23.11	23.51	22.80	23.14	23.88	23.20	21.89	21.94	22.92	23.27	23.06	23.22	23.07	0.05
27.69	26.46	26.80	26.43	26.20	27.08	26.55	24.79	25.03	26.54	26.62	25.44	26.69	26.37	0.05
28.76	28.07	27.61	26.56	27.05	27.39	28.10	26.92	25.80	26.57	27.13	27.59	26.95	27.40	0.19
27.88	27.18	27.79	26.30	27.46	27.99	27.05	26.90	25.78	27.30	28.13	26.26	26.78	27.00	0.17
35.00	35.00	34.66	33.96	35.00	35.00	35.00	35.00	32.27	35.00	35.00	35.00	33.01	34.33	0.07
32.68	30.50	31.77	31.65	30.53	31.23	30.45	31.11	29.71	31.25	31.28	30.64	31.33	30.74	0.46
35.00	32.72	35.00	32.60	31.63	32.55	34.31	35.00	30.96	35.00	33.67	35.00	32.64	33.53	0.34
24.65	26.38	27.33	24.82	25.29	24.89	32.04	25.78	26.70	25.15	25.69	26.61	24.57	26.08	0.10
27.85	27.62	28.35	27.41	27.91	28.02	27.57	25.62	26.44	27.82	27.22	27.44	27.12	27.40	0.06
27.37	25.60	26.03	25.55	25.06	25.99	26.61	25.48	24.56	25.56	26.06	25.28	25.02	25.76	0.33
30.87	27.82	28.29	27.98	27.91	28.92	27.44	28.95	26.79	28.88	29.03	27.09	28.40	28.14	0.29
27.34	28.16	28.15	26.65	26.96	26.16	28.99	24.77	25.56	25.90	25.84	27.08	26.13	27.01	-0.10
35.00	35.00	35.00	35.00	35.00	35.00	35.00	35.00	32.27	35.00	35.00	35.00	35.00	34.84	0.14
35.00		35.00	35.00	34.76	35.00	35.00	35.00	35.00	35.00	35.00	35.00	35.00	34.98	0.02
35.00	35.00	35.00	35.00	35.00	35.00	35.00	35.00	35.00	35.00	35.00	35.00	35.00	35.00	0.00
29.14	26.40	26.37	26.49	26.09	27.03	26.21	25.76	24.75	26.75	26.92	25.70	26.36	26.41	0.15
32.61	32.41	31.66	32.89	31.18	31.52	31.70	35.00	30.61	32.02	32.86	32.13	31.68	31.94	0.73
35.00	34.58	35.00	34.85	33.25	34.68	35.00	35.00	35.00	35.00	35.00	34.75	35.00	34.83	0.08
35.00	34.42	35.00	35.00	35.00	35.00	35.00	35.00	35.00	35.00	35.00	35.00	35.00	34.97	0.03
29.69	27.82	27.39	26.99	27.80	27.51	28.12	27.44	25.18	28.70	29.46	27.10	26.75	27.52	0.28
35.00	35.00	35.00	35.00	35.00	35.00	35.00	35.00	33.11	34.33	35.00	35.00	35.00	34.72	0.23
34.92	33.64	33.04	32.87	31.29	35.00	31.68	34.98	35.00	33.53	33.46	32.99	33.26	33.24	0.08
35.00	33.45	33.92	33.71	31.33	35.00	32.64	32.51	30.46	35.00	35.00	34.16	35.00	33.80	0.78
29.46	26.24	27.16	26.73	27.18	27.97	26.50	27.47	25.77	27.54	27.94	25.89	27.94	26.98	0.03
30.30	28.26	27.76	26.97	28.53	28.51	27.67	27.60	25.20	28.23	29.29	27.79	27.41	27.86	0.14
34.24	30.21	30.82	30.39	31.22	31.47	30.78	35.00	29.64	32.05	32.16	30.51	31.82	31.40	-0.02
33.06	30.64	31.59	29.87	30.48	29.55	29.76	31.23	28.67	30.90	30.24	30.71	30.74	30.51	-0.06
35.00	34.69	35.00	35.00	35.00	35.00	35.00	35.00	35.00	35.00	35.00	35.00	35.00	34.98	0.02
35.00	35.00	35.00	35.00	35.00	35.00	35.00	35.00	35.00	35.00	35.00	35.00	35.00	34.93	0.07
35.00	34.43	33.32	33.44	33.80	33.17	32.93	35.00	30.34	35.00	35.00	35.00	34.29	34.13	0.36
33.75	29.43	32.29	32.94	31.31	33.04	31.37	31.44	30.40	32.95	33.70	30.22	31.36	31.66	0.09
35.00	33.73	35.00	35.00	35.00	35.00	35.00	35.00	35.00	35.00	35.00	35.00	35.00	34.92	0.06
35.00	35.00	35.00	35.00	35.00	35.00	35.00	35.00	35.00	35.00	35.00	35.00	35.00	35.00	0.00
35.00	35.00	35.00	35.00	35.00	35.00	35.00	35.00	35.00	35.00	35.00	35.00	35.00	35.00	0.00
35.00	35.00	35.00	35.00	35.00	35.00	35.00	35.00	35.00	35.00	35.00	35.00	35.00	35.00	0.00
35.00	35.00	35.00	35.00	35.00	35.00	35.00	35.00	35.00	35.00	35.00	35.00	35.00	35.00	0.00
35.00	35.00	35.00	35.00	35.00	35.00	35.00	35.00	35.00	35.00	35.00	35.00	35.00	35.00	0.00
35.00	35.00	35.00	34.58	35.00	35.00	35.00	35.00	35.00	35.00	35.00	35.00	35.00	34.98	0.02
35.00	34.95	35.00	35.00	35.00	35.00	35.00	35.00	35.00	35.00	35.00	35.00	35.00	35.00	-0.02
35.00	35.00	35.00	35.00	35.00	35.00	35.00	35.00	35.00	35.00	35.00	35.00	35.00	35.00	0.00
35.00	35.00	34.76	35.00	35.00	35.00	35.00	35.00	35.00	35.00	35.00	35.00	35.00	34.99	0.01

Supplementary Table 1

35.00	33.14	35.00	35.00	35.00	35.00	35.00	35.00	35.00	35.00	35.00	35.00	35.00	34.89	0.11
35.00	35.00	35.00	35.00	35.00	35.00	35.00	35.00	35.00	35.00	35.00	35.00	35.00	34.95	0.05
35.00	33.67	35.00	35.00	35.00	35.00	35.00	35.00	35.00	35.00	35.00	35.00	35.00	34.92	0.07
35.00	35.00	35.00	35.00	35.00	35.00	35.00	35.00	35.00	35.00	35.00	35.00	35.00	35.00	0.00
35.00	35.00	35.00	34.47	35.00	35.00	35.00	33.25	32.01	33.81	34.57	35.00	35.00	34.44	-0.16
35.00	35.00	35.00	35.00	35.00	35.00	35.00	35.00	35.00	35.00	35.00	35.00	35.00	35.00	0.00
35.00	35.00	35.00	35.00	35.00	33.71	35.00	35.00	35.00	33.82	35.00	35.00	35.00	34.85	-0.09
35.00	34.97	35.00	35.00	35.00	34.01	35.00	35.00	35.00	35.00	35.00	35.00	35.00	34.93	0.04
33.62	29.44	29.82	27.84	29.49	30.42	29.86	30.67	28.59	29.59	30.46	29.02	29.76	29.89	0.38
35.00	35.00	33.58	34.02	33.76	35.00	35.00	35.00	31.32	35.00	35.00	32.72	35.00	34.39	0.07
32.66	32.08	32.18	31.89	32.11	32.46	35.00	35.00	31.48	31.18	31.78	31.82	32.45	32.34	0.52
35.00	35.00	35.00	35.00	35.00	35.00	35.00	35.00	35.00	33.97	35.00	34.64	35.00	34.91	0.06
31.18	28.89	28.82	28.16	28.23	28.67	28.14	28.22	26.78	28.61	29.30	27.24	28.42	28.57	0.22
35.00	35.00	35.00	35.00	35.00	35.00	35.00	35.00	33.99	35.00	35.00	35.00	35.00	34.94	0.05
35.00	35.00	35.00	35.00	35.00	35.00	35.00	35.00	35.00	35.00	35.00	35.00	35.00	35.00	0.00
35.00	35.00	35.00	35.00	35.00	35.00	35.00	35.00	35.00	35.00	35.00	35.00	35.00	35.00	0.00
29.39	27.67	27.78	27.30	27.28	28.12	28.15	27.00	26.49	27.03	28.04	27.10	27.20	27.61	0.30
29.24	26.98	27.65	27.27	27.24	28.09	27.13	26.57	25.79	27.52	27.68	26.50	27.08	27.21	0.12
35.00	35.00	35.00	35.00	33.71	35.00	35.00	35.00	35.00	35.00	35.00	35.00	35.00	34.92	0.08
29.90	28.55	28.94	27.84	28.24	28.68	27.92	27.01	26.76	27.95	28.26	27.65	28.07	28.13	0.09
35.00	35.00	35.00	35.00	35.00	35.00	35.00	35.00	35.00	35.00	35.00	35.00	35.00	35.00	0.00
35.00	34.45	34.95	34.12	34.20	35.00	35.00	35.00	35.00	35.00	34.54	35.00	35.00	34.81	0.18
35.00	35.00	35.00	35.00	35.00	35.00	35.00	35.00	35.00	35.00	35.00	35.00	35.00	35.00	0.00
35.00	35.00	35.00	35.00	35.00	35.00	35.00	35.00	35.00	35.00	35.00	35.00	35.00	35.00	0.00
35.00	35.00	35.00	35.00	35.00	35.00	35.00	35.00	35.00	35.00	35.00	35.00	35.00	35.00	0.00
35.00	35.00	35.00	35.00	35.00	35.00	35.00	35.00	35.00	35.00	35.00	35.00	35.00	35.00	0.00
35.00	35.00	35.00	35.00	35.00	35.00	35.00	35.00	35.00	35.00	35.00	35.00	35.00	35.00	-0.03
35.00	33.60	34.41	35.00	34.68	35.00	35.00	31.04	31.40	35.00	33.82	35.00	35.00	34.22	-0.27
28.36	27.99	26.44	26.95	27.16	27.24	27.81	27.30	27.41	26.74	27.89	27.05	29.00	27.41	0.51
34.25	33.77	34.01	35.00	35.00	35.00	35.00	33.60	32.99	35.00	35.00	35.00	35.00	34.13	0.16
34.58	32.08	32.47	31.64	30.50	31.29	35.00	35.00	29.65	30.75	32.76	32.40	31.45	32.13	0.36
35.00	35.00	35.00	35.00	35.00	35.00	35.00	35.00	35.00	35.00	35.00	35.00	35.00	35.00	0.00
35.00	35.00	35.00	35.00	35.00	35.00	35.00	35.00	35.00	35.00	35.00	35.00	35.00	35.00	0.00
35.00	35.00	35.00	35.00	35.00	35.00	35.00	35.00	35.00	35.00	35.00	35.00	35.00	35.00	0.00
35.00	35.00	35.00	35.00	35.00	35.00	35.00	35.00	35.00	35.00	35.00	35.00	35.00	35.00	0.00
35.00	35.00	35.00	35.00	35.00	35.00	35.00	35.00	35.00	35.00	35.00	35.00	34.87	34.99	0.01
35.00	35.00	35.00	35.00	35.00	35.00	35.00	35.00	35.00	35.00	35.00	35.00	34.71	34.95	0.04
35.00	35.00	35.00	35.00	35.00	35.00	35.00	35.00	35.00	35.00	35.00	35.00	35.00	35.00	0.00
34.68	35.00	35.00	35.00	34.59	35.00	34.24	33.86	35.00	33.61	35.00	34.76	35.00	34.69	-0.02
32.56	30.15	30.68	30.19	30.10	31.43	30.34	29.29	28.14	30.16	30.19	29.78	31.04	30.46	0.21
35.00	35.00	35.00	35.00	35.00	35.00	35.00	35.00	35.00	35.00	35.00	34.18	35.00	34.88	0.11
35.00	35.00	35.00	35.00	35.00	35.00	35.00	35.00	35.00	35.00	35.00	35.00	35.00	35.00	0.00
28.31	27.18	24.55	25.26	24.72	26.17	24.32	27.19	25.73	24.37	27.00	25.15	28.90	26.26	0.56
35.00	35.00	35.00	35.00	35.00	35.00	35.00	33.60	34.24	35.00	35.00	35.00	35.00	34.81	0.18
32.22	31.20	31.70	30.77	30.50	32.54	31.55	29.83	29.41	30.70	30.60	30.75	30.83	30.95	0.67
35.00	35.00	35.00	35.00	35.00	35.00	35.00	34.87	35.00	35.00	35.00	35.00	35.00	34.91	0.05
35.00	35.00	35.00	35.00	35.00	35.00	35.00	35.00	35.00	35.00	35.00	35.00	35.00	35.00	0.00
35.00	35.00	35.00	35.00	35.00	35.00	35.00	35.00	34.28	35.00	35.00	35.00	35.00	34.95	0.05
35.00	35.00	35.00	35.00	35.00	35.00	35.00	35.00	35.00	35.00	35.00	35.00	35.00	34.99	-0.04
35.00	35.00	34.86	34.83	35.00	35.00	34.87	31.28	31.50	33.35	35.00	34.51	35.00	34.27	-0.17
35.00	35.00	35.00	35.00	35.00	35.00	35.00	35.00	35.00	35.00	35.00	35.00	35.00	35.00	-0.03
35.00	35.00	33.79	34.48	33.43	34.33	34.09	35.00	31.99	33.31	33.79	32.63	34.07	33.99	0.23
35.00	35.00	35.00	35.00	35.00	35.00	35.00	35.00	35.00	35.00	35.00	35.00	35.00	35.00	-0.09
33.76	35.00	32.68	32.20	35.00	31.87	35.00	35.00	33.67	35.00	32.17	35.00	33.23	33.84	-0.14
35.00	35.00	35.00	35.00	35.00	35.00	35.00	35.00	35.00	35.00	35.00	35.00	35.00	35.00	0.00

Supplementary Table 1

35.00	35.00	35.00	35.00	35.00	35.00	35.00	35.00	35.00	35.00	35.00	35.00	35.00	35.00	0.00
35.00	35.00	35.00	35.00	35.00	35.00	35.00	35.00	35.00	35.00	35.00	35.00	35.00	35.00	0.00
35.00	35.00	34.36	35.00	35.00	34.79	35.00	35.00	35.00	35.00	34.40	35.00	35.00	34.89	0.01
35.00	35.00	35.00	35.00	35.00	35.00	35.00	35.00	35.00	35.00	35.00	35.00	35.00	35.00	0.00
35.00	35.00	35.00	35.00	35.00	35.00	35.00	35.00	35.00	35.00	35.00	35.00	35.00	35.00	-0.15
30.39	32.33	33.21	31.28	31.08	32.48	32.64	28.28	32.96	30.74	29.86	31.91	30.96	31.61	-0.42
35.00	33.87	34.79	33.56	33.70	35.00	35.00	32.27	35.00	35.00	35.00	32.20	35.00	34.33	0.16
35.00	35.00	35.00	35.00	35.00	35.00	35.00	34.31	35.00	35.00	35.00	34.67	35.00	34.94	-0.04
34.84	34.30	34.25	33.27	33.58	34.17	34.50	31.47	30.69	34.61	31.96	34.32	34.05	33.67	-0.08
35.00	35.00	35.00	35.00	35.00	35.00	35.00	35.00	35.00	35.00	35.00	35.00	35.00	35.00	0.00
35.00	35.00	35.00	35.00	35.00	35.00	35.00	34.93	35.00	35.00	35.00	35.00	35.00	35.00	-0.07
31.32	30.99	30.51	29.82	30.50	31.20	30.17	27.57	28.51	30.06	29.85	31.07	31.34	30.30	-0.21
35.00	35.00	35.00	35.00	35.00	35.00	35.00	35.00	35.00	35.00	35.00	35.00	35.00	35.00	-0.01
35.00	35.00	35.00	35.00	35.00	35.00	35.00	35.00	35.00	35.00	35.00	35.00	35.00	35.00	0.00
35.00	33.45	33.72	33.92	33.37	35.00	34.58	32.73	33.50	35.00	34.48	32.39	35.00	34.19	-0.04
35.00	35.00	35.00	35.00	35.00	35.00	35.00	34.16	33.36	35.00	35.00	35.00	35.00	34.85	0.06
29.50	26.41	26.37	25.48	25.60	26.34	26.42	25.73	24.52	26.71	26.35	25.66	26.38	26.35	0.20
35.00	33.02	34.37	34.05	32.92	34.42	34.52	35.00	32.32	33.02	35.00	32.90	35.00	34.21	0.43
33.88	32.96	31.39	31.70	31.30	32.23	31.36	29.71	30.31	31.74	31.55	31.89	32.29	31.85	-0.06
35.00	35.00	35.00	35.00	35.00	35.00	35.00	35.00	35.00	35.00	35.00	35.00	35.00	35.00	0.00
35.00	35.00	35.00	35.00	35.00	35.00	34.59	34.65	32.11	35.00	34.20	35.00	34.43	34.63	-0.15
35.00	35.00	35.00	35.00	34.26	34.58	35.00	35.00	34.54	34.55	35.00	35.00	34.91	34.82	-0.17
35.00	35.00	34.84	34.60	35.00	35.00	35.00	35.00	35.00	35.00	35.00	35.00	35.00	34.97	-0.06
34.68	35.00	34.04	35.00	35.00	33.24	35.00	35.00	35.00	35.00	35.00	34.17	34.36	34.64	-0.08
35.00	35.00	35.00	35.00	35.00	35.00	35.00	35.00	35.00	35.00	35.00	35.00	35.00	35.00	0.00
35.00	35.00	35.00	35.00	35.00	35.00	35.00	35.00	35.00	35.00	35.00	35.00	35.00	35.00	0.00
34.57	33.45	35.00	33.98	35.00	34.81	33.44	35.00	32.05	34.91	35.00	33.73	35.00	34.30	0.28
26.34	24.89	25.19	24.49	24.80	25.36	25.27	24.56	24.02	24.84	24.88	24.57	25.18	24.93	0.23
35.00	34.50	35.00	35.00	35.00	35.00	35.00	35.00	35.00	35.00	35.00	35.00	35.00	34.97	-0.05
35.00	35.00	35.00	35.00	35.00	35.00	35.00	35.00	35.00	35.00	35.00	35.00	35.00	35.00	0.00
35.00	35.00	35.00	35.00	35.00	35.00	35.00	35.00	35.00	34.01	35.00	35.00	35.00	34.94	0.06
27.78	25.01	25.36	24.81	24.99	25.48	24.49	25.13	23.60	25.37	25.59	24.40	25.39	25.17	0.25
35.00	35.00	35.00	35.00	35.00	35.00	35.00	35.00	35.00	35.00	35.00	35.00	35.00	35.00	0.00
35.00	33.24	34.38	34.37	33.75	35.00	33.11	34.73	33.92	34.27	34.35	33.71	35.00	34.20	0.03
35.00	35.00	35.00	34.99	35.00	34.46	35.00	35.00	35.00	35.00	35.00	35.00	35.00	34.97	0.01
35.00	35.00	35.00	35.00	35.00	35.00	35.00	35.00	35.00	35.00	35.00	35.00	35.00	35.00	0.00
34.57	35.00	35.00	33.75	35.00	35.00	32.99	31.24	30.89	34.65	33.84	33.51	33.93	34.08	-0.06
35.00	35.00	34.43	35.00	35.00	32.45	35.00	35.00	30.30	32.24	33.92	35.00	35.00	34.15	0.21
35.00	35.00	35.00	35.00	35.00	35.00	35.00	35.00	35.00	35.00	35.00	35.00	35.00	35.00	0.00
35.00	35.00	35.00	35.00	35.00	35.00	35.00	35.00	35.00	35.00	35.00	35.00	35.00	35.00	0.00
35.00	35.00	35.00	35.00	35.00	35.00	35.00	35.00	35.00	35.00	35.00	35.00	35.00	35.00	0.00
35.00	35.00	35.00	35.00	35.00	35.00	35.00	35.00	35.00	35.00	35.00	35.00	35.00	35.00	0.00
35.00	35.00	35.00	35.00	35.00	35.00	35.00	35.00	35.00	35.00	35.00	35.00	35.00	35.00	0.00
35.00	35.00	35.00	35.00	35.00	35.00	35.00	35.00	35.00	35.00	35.00	35.00	35.00	35.00	0.00

Supplementary Table 1

p-value (paired: signed-rank)
0.1773
0.3812
0.2461
0.356
0.0759
0.3812
0.0395
1
0.2659
0.084
0.5862
0.1024
0.3088
0.0552
0.084
0.193
0.1359
0.0557
0.2553
0.356
0.0684
0.2461
0.7422
0.0495
0.0086
0.0129
0.0277
0.4074
0.4074
0.4348
0.1294
0.0615
0.2868
0.4074
0.0129
0.3812
0.4375
0.9434
0.193
0.0442
0.1359
0.0113
0.1477
0.7226
0.1488
0.5625
0.0245
0.4074
0.2274
0.1488

Supplementary Table 1

0.0352
0.1626
0.2274
0.3318
0.1773
0.5228
1
0.356
0.084
0.9434
1
1
0.8311
0.0129
0.5566
0.1359
0.5862
0.5862
0.4691
0.0395
0.7583
0.4691
0.625
0.3318
0.2188
0.058
1
0.2439
0.25
0.1773
0.5
0.5862
0.6875
0.946
0.0938
1
0.5
0.5
0.75
1
0.6875
0.8457
1
0.1626
0.2659
0.1359
0.1488
0.4348
0.2868
0.4074
0.0129
0.7226
0.2097
0.2097
0.2659

Supplementary Table 1

0.1024
0.1626
0.0684
0.0113
0.1626
0.0759
0.084
0.2659
0.4348
0.5016
0.2274
0.7354
0.2969
0.2461
0.0148
1
0.084
0.2868
0.193
0.25
0.0759
0.2659
0.2163
0.3812
0.2659
0.7946
0.2097
0.3318
0.5228
0.0113
0.0174
1
0.8984
1
0.1024
0.6192
0.4074
0.3088
0.4925
1
0.9811
0.1488
0.0552
0.2274
0.0277
0.9434
0.25
0.8203
0.4348
1
1
0.9434
0.0759
1
0.2461

Supplementary Table 1

1
0.9058
0.2868
0.0684
0.8125
0.554
0.6529
0.2868
0.1359
0.1024
1
0.8313
0.5862
0.6529
0.8684
0.3011
0.6874
0.3318
0.356
0.3011
0.1773
0.2274
0.1477
0.8313
0.5228
0.6192
0.8684
0.1024
0.5245
0.1773
0.4925
0.4074
0.2461
0.0352
0.1359
0.9058
0.4348
0.1239
0.2659
0.3318
1
0.3804
0.1089
1
0.2097
0.8684
0.6875
1
0.4348
0.3318
0.4548
0.1359
0.0615
0.25
0.0785

Supplementary Table 1

1
0.7422
0.352
0.0107
0.2868
0.0759
0.2659
0.25
0.193
0.0929
0.0781
0.3828
1
0.2461
0.7226
0.8313
0.1239
0.1488
0.5771
0.1024
0.4074
0.4631
0.8313
0.0552
0.356
0.7946
1
1
1
0.6192
0.1626
0.6523
1
0.2461
0.1563
0.6417
0.0342
0.9811
0.8684
0.8684
0.8313
1
1
0.2334
0.7946
0.75
1
1
1
1
1
1
1
1
1

Supplementary Table 1

1
0.5
1
1
0.3804
1
1
0.8125
0.0277
0.791
0.1337
0.25
0.5228
0.75
1
1
0.0277
0.5228
1
0.4631
1
0.0313
1
1
1
1
1
0.4263
0.1024
0.7334
0.3303
1
1
1
1
1
0.5
1
0.9102
0.4074
0.25
1
0.554
0.1563
0.0615
0.625
1
0.5
0.5
0.6221
0.5
0.3028
0.5
0.7197
1

Supplementary Table 1

1
1
0.875
1
0.25
0.4074
0.7354
0.75
0.554
1
0.25
0.3088
1
1
1
0.875
0.193
0.2036
0.7946
1
0.4258
0.3652
1
0.7422
1
1
0.3804
0.0552
0.75
1
1
0.1773
1
1
1
1
0.7646
0.6355
1
1
1
1
1
1
